# Supplementary material for: Impact of Plasma-Activated Water Treatment on Quality and Shelf-Life of Fresh Spinach Leaves Evaluated by Comprehensive Metabolomic Analysis
Source: Foods. 2021 Dec 9;10(12):3067. doi: 10.3390/foods10123067 (PMC8702185; doi:10.3390/foods10123067)
Supplement: Supplementary file 1 [file foods-10-03067-s001.zip › foods-1456290-supplementary.pdf]

foods

Supplementary Material

Table S1. Settings for HILIC- and RPLC-HRMS analysis in MS-DIAL (v.4.6) for positive and negative ionization mode

	HILIC-HRMS		RPLC-HRMS	
**Ionization mode**	**positive**	**negative**	**positive**	**negative**
#Project				
MS1 Data type	profile	profile	profile	profile
MS2 Data type	profile	profile	profile	profile
Ion mode	positive	negative	positive	negative
Target	Metabolomics	Metabolomics	Metabolomics	Metabolomics
Mode	ddMSMS	ddMSMS	ddMSMS	ddMSMS
#Data collection parameters				
Retention time begin	0	0	0	0
Retention time end	30	40	15	15
Mass range begin	0	0	0	0
Mass range end	900	1000	1000	1000
MS2 mass range begin	0	0	0	0
MS2 mass range end	1000	1000	1000	1000
#Centroid parameters				
MS1 tolerance	0.005	0.005	0.005	0.005
MS2 tolerance	0.05	0.025	0.01	0.01
#Isotope recognition				
Maximum charged number	2	2	2	2
#Data processing				
Number of threads	2	2	4	4
#Peak detection parameters				
Smoothing method	LinearWeighted-MovingAverage	LinearWeighted-MovingAverage	LinearWeighted-MovingAverage	LinearWeighted-MovingAverage
Smoothing level	3	3	3	3
Minimum peak width	5	5	5	5
Minimum peak height	100,000	100,000	100,000	100,000
#Peak spotting parameters				
Mass slice width	0.1	0.1	0.1	0.1
#Exclusion mass list	-	-	-	-
#Deconvolution parameters				
Sigma window value	0.5	0.5	0.5	0.5
MS2Dec amplitude cut off	10	10	10	10
Exclude after precursor	TRUE	TRUE	TRUE	TRUE
Keep isotope until	0.5	0.5	0.5	0.5
Keep original precursor isotopes	TRUE	TRUE	TRUE	TRUE
#MS/MS identification setting				
Retention time tolerance	100	100	100	100
Accurate mass tolerance (MS1)	0.01	0.01	0.01	0.01
Accurate mass tolerance (MS2)	0.05	0.05	0.05	0.05
Identification score cut-off	80	80	85	80
Using retention time for scoring	UNTRUE	UNTRUE	UNTRUE	UNTRUE
Using retention time for filtering	UNTRUE	UNTRUE	UNTRUE	UNTRUE
#Post identification (retention time and accurate mass based) setting				
Retention time tolerance	0.1	0.1	0.1	0.1

Accurate mass tolerance	0.01	0.01	0.01	0.01
Identification score cut-off	85	85	85	85
#Advanced setting for identification				
Relative abundance cut-off	0	0	0	0
Top candidate report	UNTRUE	UNTRUE	UNTRUE	UNTRUE
#Adduct ion setting				
	$[M+H]^+$<br>$[M+NH_4]^+$<br>$[M+Na]^+$<br>$[M+CH_3OH+H]^+$<br>$[M+K]^+$<br>$[M+H-H_2O]^+$<br>$[2M+H]^+$	$[M-H]^-$<br>$[M-H_2O-H]^-$<br>$[M+Na-2H]^-$<br>$[M+K-2H]^-$<br>$[M+Cl]^-$	$[M+H]^+$<br>$[M+NH_4]^+$<br>$[M+Na]^+$<br>$[M+CH_3OH+H]^+$<br>$[M+K]^+$<br>$[M+H-H_2O]^+$<br>$[2M+H]^+$	$[M-H]^-$<br>$[M-H_2O-H]^-$<br>$[M+Na-2H]^-$<br>$[M+K-2H]^-$<br>$[M+Cl]^-$
#Alignment parameters setting				
Retention time tolerance	0.01	0.01	0.05	0.05
MS1 tolerance	0.015	0.015	0.01	0.015
Retention time factor	0.5	0.5	0.5	0.5
MS1 factor	0.5	0.5	0.5	0.5
Peak count filter	0	0	0	0
N% detected in at least one group	80	80	80	80
Remove feature based on peak height fold-change	TRUE	TRUE	TRUE	TRUE
Sample max / blank average	5	5	5	5
Sample average / blank average	5	5	5	5
Keep identified and annotated metabolites	UNTRUE	UNTRUE	UNTRUE	UNTRUE
Keep removable features and assign the tag for checking	TRUE	TRUE	TRUE	TRUE
Gap filling by compulsion	TRUE	TRUE	TRUE	TRUE
#Tracking of isotope labels				
Tracking of isotopic labels	UNTRUE	UNTRUE	UNTRUE	UNTRUE
#Ion mobility				
Ion mobility data	UNTRUE	UNTRUE	UNTRUE	UNTRUE

Table S2a. List of features in both positive and negative ionizations mode corresponding to clusters and their association and correlation measured by HILIC-HRMS.

Source	Cluster 1	Cluster 2	Cluster MSdial 1	Cluster MSdial 2	Cpd ID 1	Cpd ID 2	Sample Nature	Adduct 1	Adduct 2	RT 1	RT 2	Mass 1	Mass 2	Correlation
MSDial	33	33	48	48	neg_1418	neg_2027	similar chromatogram in higher mz	$[M-H]^-$	$[M-2H]^{2-}$	10.253	10.215	285.0612	400.0901	1.000
MSDial	46	46	62	62	neg_1927	neg_2321	Pearson correlation	$[M-H]^-$	$[M-H_2O-H]^-$	34.346	34.31	374.7722	474.7280	0.928
MSDial	46	46	62	62	neg_1927	neg_2500	Pearson correlation	$[M-H]^-$	$[M-H_2O-H]^-$	34.346	34.343	374.7722	536.7306	0.951
MSDial	46	46	62	62	neg_2321	neg_2500	Pearson correlation	$[M-H_2O-H]^-$	$[M-H_2O-H]^-$	34.310	34.343	474.7280	536.7306	0.942
MSDial	49	49	66	66	neg_1684	neg_2360	similar chromatogram in higher mz	$[M-H]^-$	$[M-H]^-$	3.938	3.934	327.2172	485.2748	1.000
Adducts/Neutral losses	51	51	288	68	pos_2941	neg_2343	pol / adduct	$[M+H]^+$	$[M-H]^-$	4.849	4.844	481.3143	479.2999	0.943
Adducts/Neutral losses	51	51	288	68	pos_2941	neg_2343	pol / adduct	$[M+H]^+$	$[M-H]^-$	4.849	4.844	481.3143	479.2999	0.943
MSDial	51	51	68	68	neg_2343	neg_2395	pol / adduct	$[M-H]^-$	$[M+K-2H]^-$	4.844	4.852	479.2999	495.2950	1.000
MSDial	51	51	68	68	neg_2343	neg_2395	Pearson correlation	$[M-H]^-$	$[M+K-2H]^-$	4.844	4.850	479.2999	495.2950	0.944
MSDial	51	51	68	68	neg_2343	neg_2395	pol / adduct	$[M-H]^-$	$[M+K-2H]^-$	4.844	4.852	479.2999	495.2950	1.000
MSDial	53	53	71	71	neg_1371	neg_2417	found in higher mz's MsMs	$[M-H]^-$	$[M-H]^-$	3.456	3.460	277.2166	505.2580	1.000
Adducts/Neutral losses	61	61	315	82	pos_3372	neg_2625	pol / adduct	$[M+H]^+$	$[M-H]^-$	17.492	17.493	613.1593	611.1426	0.985
Adducts/Neutral losses	61	61	315	82	pos_3372	neg_2625	pol / adduct	$[M+H]^+$	$[M-H]^-$	17.492	17.493	613.1593	611.1426	0.985
MSDial	61	61	82	82	neg_1547	neg_2625	similar chromatogram in higher mz	$[M-H]^-$	$[M-H]^-$	17.495	17.493	305.0688	611.1426	1.000
MSDial	61	61	82	82	neg_1547	neg_2625	Pearson correlation	$[M-H]^-$	$[M-H]^-$	17.495	17.493	305.0688	611.1426	0.995
MSDial	75	75	108	108	neg_1357	neg_283	similar chromatogram in higher mz	$[M+Na-2H]^-$	$[M-H]^-$	16.599	16.599	275.0215	133.0139	1.000
MSDial	75	75	108	108	neg_17	neg_283	Pearson correlation	$[M-H_2O-H]^-$	$[M-H]^-$	16.626	16.599	71.01376	133.0139	0.932
MSDial	75	75	108	108	neg_1357	neg_1434	pol / adduct	$[M+Na-2H]^-$	$[M+Cl]^-$	16.599	16.616	275.0215	289.0172	1.000
MSDial	78	78	113	113	neg_1681	neg_308	found in higher mz's MsMs	[M-H]-	$[M-H]^-$	12.549	12.558	327.0560	135.0298	1.000
MSDial	79	79	114	114	neg_1680	neg_311	similar chromatogram in higher mz	[M-H]-	$[M-H]^-$	12.936	12.943	327.0557	135.0299	1.000

MSDial	79	79	114	114	neg_1680	neg_311	found in higher mz's MsMs	[M-H]-	$[M-H]^-$	12.936	12.943	327.0557	135.0299	1.000
Adducts/Neutral losses	86	86	122	122	neg_387	neg_244	neutral loss	H_2O	H_2O	14.732	14.742	146.0457	128.0352	0.990
Adducts/Neutral losses	86	86	348	122	pos_400	neg_244	pol / adduct	$[M+H]^+$	$[M-H_2O-H]^-$	14.730	14.742	148.0603	128.0352	0.959
Adducts/Neutral losses	86	86	348	122	pos_400	neg_387	pol / adduct	$[M+H]^+$	$[M-H]^-$	14.730	14.732	148.0603	146.0457	0.979
Adducts/Neutral losses	86	86	348	122	pos_400	neg_387	pol / adduct	$[M+H]^+$	$[M-H]^-$	14.730	14.732	148.0603	146.0457	0.979
MSDial	86	86	122	122	neg_244	neg_387	found in higher mz's MsMs	$[M-H]^-$	$[M-H]^-$	14.742	14.732	128.0352	146.0457	1.000
MSDial	86	86	122	122	neg_244	neg_387	Pearson correlation	$[M-H]^-$	$[M-H]^-$	14.742	14.732	128.0352	146.0457	0.990
MSDial	87	87	123	123	neg_1652	neg_396	similar chromatogram in higher mz	$[M-H]^-$	$[M-H]^-$	15.208	15.23	323.0275	147.0295	1.000
MSDial	87	87	123	123	neg_396	neg_487	similar chromatogram in higher mz	$[M-H]^-$	$[M-H]^-$	15.230	15.258	147.0295	159.0296	1.000
MSDial	99	99	137	137	neg_1539	neg_514	Pearson correlation	$[M-H_2O-H]^-$	$[M-H_2O-H]^-$	4.474	4.483	303.1449	161.0453	0.944
MSDial	101	101	141	141	neg_1477	neg_416	found in higher mz's MsMs	$[M+K-2H]^-$	$[M-H_2O-H]^-$	14.349	14.354	295.0457	149.0089	1.000
MSDial	101	101	141	141	neg_1477	neg_416	Pearson correlation	$[M+K-2H]^-$	$[M-H_2O-H]^-$	14.349	14.354	295.0457	149.0089	0.987
MSDial	101	101	141	141	neg_286	neg_416	similar chromatogram in higher mz	$[M-H]^-$	$[M-H_2O-H]^-$	14.329	14.354	133.0139	149.0089	1.000
MSDial	101	101	141	141	neg_286	neg_530	Pearson correlation	$[M-H]^-$	$[M-H]^-$	14.329	14.331	133.0139	163.0398	0.996
Adducts/Neutral losses	102	102	143	143	neg_535	neg_374	neutral loss	H_2O	H_2O	11.738	11.737	163.0398	145.0295	0.964
MSDial	102	102	143	143	neg_374	neg_535	pol / adduct	$[M-H_2O-H]^-$	$[M-H]^-$	11.737	11.738	145.0295	163.0398	1.000
MSDial	102	102	143	143	neg_2715	neg_535	found in higher mz's MsMs	$[2M-H]^-$	$[M-H]^-$	11.736	11.738	675.1209	163.0398	1.000
MSDial	102	102	143	143	neg_374	neg_535	Pearson correlation	$[M-H_2O-H]^-$	$[M-H]^-$	11.737	11.738	145.0295	163.0398	0.964
MSDial	102	102	143	143	neg_374	neg_535	pol / adduct	$[M-H_2O-H]^-$	$[M-H]^-$	11.737	11.738	145.0295	163.0398	1.000
MSDial	115	115	159	159	neg_2767	neg_668	similar chromatogram in higher mz	$[M-H]^-$	$[M-H]^-$	12.048	12.042	713.1193	179.0559	1.000
Adducts/Neutral losses	116	116	160	160	neg_67	neg_16	neutral loss	H_2O	H_2O	13.367	13.370	89.02444	71.01375	0.988
Adducts/Neutral losses	116	116	160	160	neg_513	neg_16	neutral loss	$C_3H_6O_3$	$C_3H_6O_3$	13.400	13.370	161.0452	71.01375	0.987

MSDial	116	116	160	160	neg_16	neg_67	pol / adduct	$[M-H_2O-H]^-$	$[M-H]^-$	13.370	13.367	71.01375	89.02444	1.000
MSDial	116	116	160	160	neg_513	neg_67	Pearson correlation	$[M-H_2O-H]^-$	$[M-H]^-$	13.400	13.367	161.0452	89.02444	0.991
MSDial	116	116	160	160	neg_16	neg_67	Pearson correlation	$[M-H_2O-H]^-$	$[M-H]^-$	13.370	13.367	71.01375	89.02444	0.988
MSDial	116	116	160	160	neg_16	neg_67	pol / adduct	$[M-H_2O-H]^-$	$[M-H]^-$	13.370	13.367	71.01375	89.02444	1.000
MSDial	116	116	160	160	neg_16	neg_67	found in higher mz's MsMs	$[M-H_2O-H]^-$	$[M-H]^-$	13.370	13.367	71.01375	89.02444	1.000
MSDial	116	116	160	160	neg_16	neg_513	Pearson correlation	$[M-H_2O-H]^-$	$[M-H_2O-H]^-$	13.370	13.400	71.01375	161.0452	0.987
MSDial	120	120	165	165	neg_641	neg_68	found in higher mz's MsMs	$[M-H]^-$	$[M-H]^-$	15.360	15.363	175.0471	89.03559	1.000
MSDial	120	120	165	165	neg_641	neg_68	Pearson correlation	$[M-H]^-$	$[M-H]^-$	15.360	15.363	175.0471	89.03559	0.989
Adducts/Neutral losses	120	120	381	165	pos_611	neg_641	pol / adduct	$[M+H]^+$	$[M-H]^-$	15.368	15.360	177.0615	175.0471	0.970
Adducts/Neutral losses	120	120	381	165	pos_611	neg_641	pol / adduct	$[M+H]^+$	$[M-H]^-$	15.368	15.360	177.0615	175.0471	0.970
MSDial	120	120	381	381	pos_1477	pos_611	Pearson correlation	$[M+H]^+$	$[M+H]^+$	15.331	15.368	280.1249	177.0615	0.915
MSDial	134	134	185	185	neg_721	neg_878	similar chromatogram in higher mz	$[M-H]^-$	$[M-H]^-$	11.118	11.147	187.0974	206.0823	1.000
MSDial	137	137	188	188	neg_179	neg_91	pol / adduct	$[M-H]^-$	$[M-H_2O-H]^-$	15.636	15.633	117.0192	99.00872	1.000
MSDial	137	137	188	188	neg_179	neg_91	pol / adduct	$[M-H]^-$	$[M-H^2O-H]^-$	15.636	15.633	117.0192	99.00872	1.000
MSDial	137	137	188	188	neg_179	neg_91	Pearson correlation	$[M-H]^-$	$[M-H_2O-H]^-$	15.636	15.633	117.0192	99.00872	0.990
Adducts/Neutral losses	137	137	188	188	neg_179	neg_91	neutral loss	H_2O	H_2O	15.636	15.633	117.0192	99.00872	0.990
MSDial	137	137	188	188	neg_179	neg_91	found in higher mz's MsMs	[M-H]-	$[M-H_2O-H]^-$	15.636	15.633	117.0192	99.00872	1.000
Adducts/Neutral losses	148	148	200	140	pos_103	neg_53	pol / adduct	$[M+H]^+$	$[M-H]^-$	14.669	14.693	90.05482	88.04023	0.961
Adducts/Neutral losses	148	148	200	140	pos_103	neg_53	pol / adduct	$[M+H]^+$	$[M-H]^-$	14.669	14.693	90.05482	88.04023	0.961
Adducts/Neutral losses	170	170	221	16	pos_1455	neg_1362	pol / adduct	$[M+H]^+$	$[M-H]^-$	15.523	15.522	277.1384	275.1241	0.988
Adducts/Neutral losses	170	170	221	16	pos_1455	neg_1362	pol / adduct	$[M+H]^+$	$[M-H]^-$	15.523	15.522	277.1384	275.1241	0.988
MSDial	171	171	309	309	pos_147	pos_33	found in higher mz's MsMs	$[M+H]^+$	$[M+H]^+$	15.264	15.261	104.0704	69.03349	1.000

MSDial	171	171	309	309	pos_147	pos_33	Pearson correlation	$[M+H]^+$	$[M+H]^+$	15.264	15.261	104.0704	69.03349	0.982
Adducts/Neutral losses	171	171	309	2	pos_147	neg_107	pol / adduct	$[M+H]^+$	$[M-H]^-$	15.264	15.277	104.0704	102.0559	0.908
Adducts/Neutral losses	171	171	309	2	pos_147	neg_107	pol / adduct	$[M+H]^+$	$[M-H]^-$	15.264	15.277	104.0704	102.0559	0.908
Adducts/Neutral losses	175	175	225	5	pos_154	neg_119	pol / adduct	$[M+H]^+$	$[M-H]^-$	15.914	15.915	106.0497	104.0353	0.923
Adducts/Neutral losses	175	175	225	5	pos_154	neg_119	pol / adduct	$[M+H]^+$	$[M-H]^-$	15.914	15.915	106.0497	104.0353	0.923
Adducts/Neutral losses	176	176	243	21	pos_1575	neg_1437	pol / adduct	$[M+H]^+$	$[M-H]^-$	13.738	13.742	291.0969	289.0826	0.982
Adducts/Neutral losses	176	176	243	21	pos_1575	neg_1437	pol / adduct	$[M+H]^+$	$[M-H]^-$	13.738	13.742	291.0969	289.0826	0.982
Adducts/Neutral losses	176	176	243	243	pos_1998	pos_1575	neutral loss	C_2H_7N	C_2H_7N	13.752	13.738	336.1543	291.0969	0.119
MSDial	176	176	243	243	pos_1575	pos_1998	found in higher mz's MsMs	$[M+H]^+$	$[M+H]^+$	13.738	13.752	291.0969	336.1543	1.000
Adducts/Neutral losses	179	179	228	228	pos_1603	pos_1456	neutral loss	O	O	3.523	3.519	293.2103	277.2159	0.950
Adducts/Neutral losses	179	179	228	22	pos_1603	neg_1453	pol / adduct	$[M+H]^+$	$[M-H]^-$	3.523	3.524	293.2103	291.1967	0.731
Adducts/Neutral losses	179	179	228	22	pos_1603	neg_1453	pol / adduct	$[M+H]^+$	$[M-H]^-$	3.523	3.524	293.2103	291.1967	0.731
Adducts/Neutral losses	179	179	219	22	pos_1440	neg_1453	pol / adduct	$[M+H-H_2O]^+$	$[M-H]^-$	3.568	3.524	275.2002	291.1967	0.883
Adducts/Neutral losses	179	179	219	22	pos_1440	neg_1453	pol / adduct	$[M+H-H_2O]^+$	$[M-H]^-$	3.568	3.524	275.2002	291.1967	0.883
MSDial	179	179	228	228	pos_1456	pos_1603	Pearson correlation	$[M+H-H_2O]^+$	$[M+H]^+$	3.519	3.523	277.2159	293.2103	0.950
MSDial	179	179	228	228	pos_1456	pos_1603	pol / adduct	$[M+H-H_2O]^+$	$[M+H]^+$	3.519	3.523	277.2159	293.2103	1.000
Adducts/Neutral losses	179	179	228	88	pos_1456	neg_1468	pol / adduct	$[M+H-H_2O]^+$	$[M-H]^-$	3.519	3.520	277.2159	293.2124	0.915
Adducts/Neutral losses	179	179	228	88	pos_1456	neg_1468	pol / adduct	$[M+H-H_2O]^+$	$[M-H]^-$	3.519	3.520	277.2159	293.2124	0.915

MSDial	179	179	88	88	neg_1468	neg_2672	similar chromatogram in higher mz	$[M-H]^-$	$[M-H]^-$	3.520	3.500	293.2124	641.2618	1.000
Adducts/Neutral losses	192	192	242	32	pos_199	neg_172	pol / adduct	$[M+H]^+$	$[M-H]^-$	12.224	12.235	118.0860	116.0714	0.926
Adducts/Neutral losses	192	192	242	32	pos_199	neg_172	pol / adduct	$[M+H]^+$	$[M-H]^-$	12.224	12.235	118.0860	116.0714	0.926
Adducts/Neutral losses	196	196	246	42	pos_204	neg_184	pol / adduct	$[M+H]^+$	$[M-H]^-$	14.468	14.488	120.0652	118.0507	0.982
Adducts/Neutral losses	196	196	246	42	pos_204	neg_184	pol / adduct	$[M+H]^+$	$[M-H]^-$	14.468	14.488	120.0652	118.0507	0.982
Adducts/Neutral losses	200	200	251	36	pos_2089	neg_1779	pol / adduct	$[M+H]^+$	$[M-H]^-$	12.695	12.697	346.0541	344.0398	0.994
Adducts/Neutral losses	200	200	251	36	pos_2089	neg_1779	pol / adduct	$[M+H]^+$	$[M-H]^-$	12.695	12.697	346.0541	344.0398	0.994
Adducts/Neutral losses	201	201	252	38	pos_2100	neg_1781	pol / adduct	$[M+H]^+$	$[M-H]^-$	12.078	12.082	347.0738	345.0601	0.958
Adducts/Neutral losses	201	201	252	38	pos_2100	neg_1781	pol / adduct	$[M+H]^+$	$[M-H]^-$	12.078	12.082	347.0738	345.0601	0.958
MSDial	203	203	142	142	neg_1787	neg_532	similar chromatogram in higher mz	$[M-H]^-$	$[M-H]^-$	13.589	13.590	346.0548	163.0398	1.000
Adducts/Neutral losses	203	203	254	142	pos_2107	neg_1787	pol / adduct	$[M+H]^+$	$[M-H]^-$	13.583	13.589	348.0698	346.0548	0.994
Adducts/Neutral losses	203	203	254	142	pos_2107	neg_1787	pol / adduct	$[M+H]^+$	$[M-H]^-$	13.583	13.589	348.0698	346.0548	0.994
Adducts/Neutral losses	204	204	255	40	pos_2108	neg_1788	pol / adduct	$[M+H]^+$	$[M-H]^-$	12.673	12.681	348.0701	346.0550	0.992
Adducts/Neutral losses	204	204	255	40	pos_2108	neg_1788	pol / adduct	$[M+H]^+$	$[M-H]^-$	12.673	12.681	348.0701	346.0550	0.992
Adducts/Neutral losses	206	206	261	35	pos_2234	neg_1762	pol / adduct	[M+Na]+	$[M-H]^-$	16.921	16.919	365.1049	341.1082	0.907
Adducts/Neutral losses	206	206	261	35	pos_2196	neg_1762	pol / adduct	$[M+NH_4]^+$	$[M-H]^-$	16.918	16.919	360.1487	341.1082	0.951
Adducts/Neutral losses	206	206	261	35	pos_2234	neg_1762	pol / adduct	$[M+Na]^+$	$[M-H]^-$	16.921	16.919	365.1049	341.1082	0.907

Adducts/Neutral losses	206	206	261	35	pos_2196	neg_1762	pol / adduct	$[M+NH_4]^+$	$[M-H]^-$	16.918	16.919	360.1487	341.1082	0.951
MSDial	206	206	261	261	pos_2196	pos_2234	pol / adduct	$[M+NH_4]^+$	$[M+Na]^+$	16.918	16.921	360.1487	365.1049	1.000
Adducts/Neutral losses	208	208	258	43	pos_2227	neg_1868	pol / adduct	$[M+H]^+$	$[M-H]^-$	15.844	15.846	364.0649	362.0503	0.995
Adducts/Neutral losses	208	208	258	43	pos_2227	neg_1868	pol / adduct	$[M+H]^+$	$[M-H]^-$	15.844	15.846	364.0649	362.0503	0.995
MSDial	210	210	260	260	pos_1738	pos_2233	Pearson correlation	$[M+Na]^+$	$[M+Na]^+$	14.183	14.216	307.0995	365.1048	0.939
Adducts/Neutral losses	217	217	269	73	pos_252	neg_246	pol / adduct	$[M+H]^+$	$[M-H]^-$	10.185	10.182	130.0496	128.0352	0.963
Adducts/Neutral losses	217	217	269	73	pos_252	neg_246	pol / adduct	$[M+H]^+$	$[M-H]^-$	10.185	10.182	130.0496	128.0352	0.963
MSDial	219	219	271	271	pos_102	pos_259	similar chromatogram in higher mz	$[M+H]^+$	$[M+H]^+$	6.682	6.752	90.0548	130.0861	1.000
Adducts/Neutral losses	219	219	271	125	pos_259	neg_425	pol / adduct	$[M+H]^+$	$[M+Na-2H]^-$	6.752	6.732	130.0861	150.0556	0.724
MSDial	219	219	125	125	neg_1914	neg_425	similar chromatogram in higher mz	$[M-H]^-$	$[M-H]^-$	6.751	6.732	371.0975	150.0556	1.000
MSDial	228	228	403	403	pos_274	pos_86	found in higher mz's MsMs	$[M+H]^+$	$[M+H]^+$	10.954	10.948	132.1016	86.09619	1.000
MSDial	228	228	403	403	pos_274	pos_86	Pearson correlation	$[M+H]^+$	$[M+H]^+$	10.954	10.948	132.1016	86.09619	0.990
Adducts/Neutral losses	228	228	403	87	pos_274	neg_267	pol / adduct	$[M+H]^+$	$[M-H]^-$	10.954	10.935	132.1016	130.0872	0.904
Adducts/Neutral losses	228	228	403	87	pos_274	neg_267	pol / adduct	$[M+H]^+$	$[M-H]^-$	10.954	10.935	132.1016	130.0872	0.904
MSDial	228	228	403	403	pos_274	pos_3292	found in higher mz's MsMs	$[M+H]^+$	$[M+H]^+$	10.954	10.935	132.1016	582.312	1.000
Adducts/Neutral losses	232	232	283	25	pos_279	neg_149	pol / adduct	$[M+H]^+$	$[M-H_2O-H]^-$	15.427	15.413	133.0605	113.0356	0.973
Adducts/Neutral losses	233	233	284	138	pos_287	neg_280	pol / adduct	$[M+H]^+$	$[M-H]^-$	15.132	15.107	134.0446	132.0300	0.926
Adducts/Neutral losses	233	233	284	138	pos_287	neg_280	pol / adduct	$[M+H]^+$	$[M-H]^-$	15.132	15.107	134.0446	132.0300	0.926
Adducts/Neutral losses	237	237	303	63	pos_2928	neg_2334	pol / adduct	$[M+H]^+$	$[M-H]^-$	4.135	4.137	478.2917	476.2766	0.991

Adducts/Neutral losses	237	237	303	63	pos_2928	neg_2334	pol / adduct	[M+H]$^+$	[M-H]$^-$	4.135	4.137	478.2917	476.2766	0.991
Adducts/Neutral losses	237	237	303	303	pos_3109	pos_2928	neutral loss	C_3H_6	C_3H_6	4.107	4.135	520.3387	478.2917	0.923
MSDial	237	237	303	303	pos_2928	pos_3109	Pearson correlation	[M+H]$^+$	[M+H]$^+$	4.135	4.107	478.2917	520.3387	0.923
Adducts/Neutral losses	239	239	290	59	pos_2967	neg_2296	pol / adduct	[M+NH_4]$^+$	[M-H]$^-$	7.620	7.624	488.1413	469.1001	0.984
Adducts/Neutral losses	239	239	290	59	pos_2967	neg_2296	pol / adduct	[M+NH_4]$^+$	[M-H]$^-$	7.620	7.624	488.1413	469.1001	0.984
Adducts/Neutral losses	240	240	291	111	pos_300	neg_302	pol / adduct	[M+H]$^+$	[M-H]$^-$	8.977	8.969	136.0616	134.0471	0.986
Adducts/Neutral losses	240	240	291	111	pos_300	neg_302	pol / adduct	[M+H]$^+$	[M-H]$^-$	8.977	8.969	136.0616	134.0471	0.986
MSDial	242	242	158	158	neg_2320	neg_655	similar chromatogram in higher mz	[M-H]$^-$	[M+H]$^+$	4.167	4.203	474.2618	177.0555	1.000
MSDial	242	242	158	158	neg_2238	neg_655	similar chromatogram in higher mz	[M-H]$^-$	[M+H]$^+$	4.205	4.203	452.2769	177.0555	1.000
Adducts/Neutral losses	242	242	293	158	pos_2783	neg_2238	pol / adduct	[M+H]$^+$	[M-H]$^-$	4.200	4.205	454.2927	452.2769	0.998
Adducts/Neutral losses	242	242	293	158	pos_2783	neg_2238	pol / adduct	[M+H]$^+$	[M-H]$^-$	4.200	4.205	454.2927	452.2769	0.998
Adducts/Neutral losses	242	242	293	293	pos_3014	pos_2783	neutral loss	C_3H_6	C_3H_6	4.154	4.200	496.3394	454.2927	0.950
Adducts/Neutral losses	242	242	293	158	pos_2783	neg_2320	pol / adduct	[M+H]$^+$	[M+Na-2H]-	4.200	4.167	454.2927	474.2618	0.692
MSDial	242	242	293	293	pos_2783	pos_3014	Pearson correlation	[M+H]$^+$	[M+H]$^+$	4.200	4.154	454.2927	496.3394	0.950
MSDial	243	243	110	110	neg_1304	neg_301	Pearson correlation	[M-H]-	[M-H]-	8.417	8.416	266.0894	134.0471	0.997
MSDial	243	243	110	110	neg_1304	neg_301	found in higher mz's MsMs	[M-H]-	[M-H]-	8.417	8.416	266.0894	134.0471	1.000
Adducts/Neutral losses	243	243	294	110	pos_302	neg_301	pol / adduct	[M+H]$^+$	[M-H]-	8.417	8.416	136.0618	134.0471	0.972
Adducts/Neutral losses	243	243	110	110	neg_1304	neg_301	neutral loss	D-ribose (-H_2O) (ribosylation)	D-ribose (-H_2O) (ribosylation)	8.417	8.416	266.0894	134.0471	0.997
MSDial	243	243	110	110	neg_1688	neg_301	Pearson correlation	[M-H]$^-$	[M-H]$^-$	8.445	8.416	328.0454	134.0471	0.910

Adducts/Neutral losses	243	243	294	110	pos_302	neg_301	pol / adduct	$[M+H]^+$	$[M-H]^-$	8.417	8.416	136.0618	134.0471	0.972
MSDial	243	243	110	110	neg_1688	neg_301	similar chromatogram in higher mz	$[M-H]^-$	$[M-H]^-$	8.445	8.416	328.0454	134.0471	1.000
MSDial	243	243	110	110	neg_1688	neg_301	found in higher mz's MsMs	$[M-H]^-$	$[M-H]^-$	8.445	8.416	328.0454	134.0471	1.000
MSDial	243	243	294	294	pos_1372	pos_302	Pearson correlation	$[M+H]^+$	$[M+H]^+$	8.420	8.417	268.1030	136.0618	0.998
Adducts/Neutral losses	243	243	294	294	pos_1372	pos_302	neutral loss	D-ribose (-H_2O) (ribosylation)	D-ribose (-H_2O) (ribosylation)	8.420	8.417	268.1030	136.0618	0.998
MSDial	243	243	294	294	pos_1372	pos_302	found in higher mz's MsMs	$[M+H]^+$	$[M+H]^+$	8.420	8.417	268.103	136.0618	1.000
Adducts/Neutral losses	243	243	294	110	pos_1372	neg_1304	pol / adduct	$[M+H]^+$	$[M-H]^-$	8.420	8.417	268.103	266.0894	0.982
Adducts/Neutral losses	243	243	294	110	pos_1372	neg_1304	pol / adduct	$[M+H]^+$	$[M-H]^-$	8.420	8.417	268.103	266.0894	0.982
Adducts/Neutral losses	243	243	294	110	pos_1939	neg_1688	pol / adduct	$[M+H]^+$	$[M-H]^-$	8.452	8.445	330.0585	328.0454	0.996
Adducts/Neutral losses	243	243	294	110	pos_1939	neg_1688	pol / adduct	$[M+H]^+$	$[M-H]^-$	8.452	8.445	330.0585	328.0454	0.996
MSDial	243	243	110	110	neg_1304	neg_1688	Pearson correlation	$[M-H]^-$	$[M-H]^-$	8.417	8.445	266.0894	328.0454	0.911
MSDial	243	243	294	294	pos_1372	pos_1939	Pearson correlation	$[M+H]^+$	$[M+H]^+$	8.420	8.452	268.1030	330.0585	0.910
MSDial	244	244	295	295	pos_3046	pos_631	similar chromatogram in higher mz	$[M-H]^-$	$[M+H]^+$	4.315	4.358	505.0955	180.1016	1.000
MSDial	244	244	295	295	pos_1048	pos_3046	similar chromatogram in higher mz	$[M+H]^+$	$[M-H]^-$	4.30	4.315	230.2471	505.0955	1.000
Adducts/Neutral losses	246	246	298	70	pos_3056	neg_2415	pol / adduct	$[M+H]^+$	$[M-H]^-$	9.857	9.863	507.1130	505.0989	0.903
Adducts/Neutral losses	246	246	298	70	pos_3056	neg_2415	pol / adduct	$[M+H]^+$	$[M-H]^-$	9.857	9.863	507.113	505.0989	0.903
Adducts/Neutral losses	252	252	305	116	pos_315	neg_316	pol / adduct	$[M+H]^+$	$[M-H]^-$	11.239	11.239	138.0548	136.0403	0.923
Adducts/Neutral losses	252	252	305	116	pos_315	neg_316	pol / adduct	$[M+H]^+$	$[M-H]^-$	11.239	11.239	138.0548	136.0403	0.923
Adducts/Neutral losses	257	257	318	78	pos_3397	neg_2593	pol / adduct	$[M+CH_3OH+H]^+$	$[M-H]^-$	4.599	4.577	624.2261	590.1887	0.627

Adducts/Neutral losses	257	257	318	78	pos_3397	neg_2593	pol / adduct	$[M+CH_3OH+H]^+$	$[M-H]^-$	4.599	4.577	624.2261	590.1887	0.627
Adducts/Neutral losses	257	257	311	78	pos_3320	neg_2593	pol / adduct	$[M+H]^+$	$[M-H]^-$	4.578	4.577	592.2014	590.1887	0.971
Adducts/Neutral losses	257	257	311	78	pos_3320	neg_2593	pol / adduct	$[M+H]^+$	$[M-H]^-$	4.578	4.577	592.2014	590.1887	0.971
Adducts/Neutral losses	259	259	313	80	pos_3363	neg_2597	pol / adduct	$[M+NH_4]^+$	$[M-H]^-$	7.965	7.957	610.2679	591.2273	0.940
Adducts/Neutral losses	259	259	313	80	pos_3363	neg_2597	pol / adduct	$[M+NH_4]^+$	$[M-H]^-$	7.965	7.957	610.2679	591.2273	0.940
MSDial	259	259	313	313	pos_312	pos_3363	Pearson correlation	$[M+H]^+$	$[M+NH_4]^+$	7.929	7.965	138.0547	610.2679	0.901
Adducts/Neutral losses	260	260	314	79	pos_3364	neg_2596	pol / adduct	$[M+NH_4]^+$	$[M-H]^-$	8.578	8.589	610.2681	591.2271	0.952
Adducts/Neutral losses	260	260	314	79	pos_3364	neg_2596	pol / adduct	$[M+NH_4]^+$	$[M-H]^-$	8.578	8.589	610.2681	591.2271	0.952
Adducts/Neutral losses	266	266	322	89	pos_3487	neg_2695	pol / adduct	$[M+H]^+$	$[M-H]^-$	13.963	13.96	657.1633	655.1492	0.979
Adducts/Neutral losses	266	266	322	89	pos_3487	neg_2695	pol / adduct	$[M+H]^+$	$[M-H]^-$	13.963	13.96	657.1633	655.1492	0.979
MSDial	266	266	322	322	pos_1279	pos_3487	similar chromatogram in higher mz	[M]+	$[M+H]^+$	13.972	13.963	258.1093	657.1633	1.000
MSDial	273	273	94	94	neg_1698	neg_2440	similar chromatogram in higher mz	$[M-H]^-$	$[M-H]^-$	3.857	3.862	329.2321	513.3070	1.000
Adducts/Neutral losses	273	273	328	94	pos_3548	neg_2722	pol / adduct	$[M+H]^+$	$[M-H]^-$	3.876	3.869	679.2970	677.2809	0.996
Adducts/Neutral losses	273	273	328	94	pos_3548	neg_2722	pol / adduct	$[M+H]^+$	$[M-H]^-$	3.876	3.869	679.2970	677.2809	0.996
MSDial	273	273	94	94	neg_2629	neg_2722	Pearson correlation	$[M-H]^-$	$[M-H]^-$	3.911	3.869	613.2654	677.2809	0.921
MSDial	273	273	94	94	neg_1698	neg_2722	similar chromatogram in higher mz	$[M-H]^-$	$[M-H]^-$	3.857	3.869	329.2321	677.2809	1.000
MSDial	274	274	329	329	pos_3523	pos_3571	pol / adduct	$[M+H]^+$	$[M+Na]^+$	10.885	10.888	671.1809	693.1622	1.000
MSDial	274	274	329	329	pos_3523	pos_3695	similar chromatogram in higher mz	$[M+H]^+$	$[M+H]^+$	10.885	10.895	671.1809	802.2761	1.000
Adducts/Neutral losses	275	275	330	97	pos_3572	neg_2737	pol / adduct	$[M+H]^+$	$[M-H]^-$	4.353	4.335	693.2744	691.2607	0.913

Adducts/Neutral losses	275	275	330	97	pos_3572	neg_2737	pol / adduct	$[M+H]^+$	$[M-H]^-$	4.353	4.335	693.2744	691.2607	0.913
Adducts/Neutral losses	276	276	331	98	pos_3589	neg_2742	pol / adduct	$[M+H]^+$	$[M-H]^-$	3.603	3.602	697.1386	695.1254	0.975
Adducts/Neutral losses	276	276	331	98	pos_3589	neg_2742	pol / adduct	$[M+H]^+$	$[M-H]^-$	3.603	3.602	697.1386	695.1254	0.975
Adducts/Neutral losses	278	278	333	99	pos_3595	neg_2746	pol / adduct	$[M+H]^+$	$[M-H]^-$	4.534	4.536	699.1549	697.1415	0.978
Adducts/Neutral losses	278	278	333	99	pos_3595	neg_2746	pol / adduct	$[M+H]^+$	$[M-H]^-$	4.534	4.536	699.1549	697.1415	0.978
MSDial	279	279	354	354	pos_189	pos_36	similar chromatogram in higher mz	$[M+H]^+$	$[M+H]^+$	12.446	12.447	116.0705	70.06505	1.000
MSDial	279	279	354	354	pos_189	pos_36	Pearson correlation	$[M+H]^+$	$[M+H]^+$	12.446	12.447	116.0705	70.06505	0.998
MSDial	279	279	354	354	pos_189	pos_36	found in higher mz's MsMs	$[M+H]^+$	$[M+H]^+$	12.446	12.447	116.0705	70.06505	1.000
Adducts/Neutral losses	279	279	354	29	pos_189	neg_156	pol / adduct	$[M+H]^+$	$[M-H]^-$	12.446	12.453	116.0705	114.0560	0.961
Adducts/Neutral losses	279	279	354	29	pos_189	neg_156	pol / adduct	$[M+H]^+$	$[M-H]^-$	12.446	12.453	116.0705	114.0560	0.961
MSDial	279	279	354	354	pos_1507	pos_189	similar chromatogram in higher mz	$[M+H]^+$	$[M+H]^+$	12.409	12.446	284.0987	116.0705	1.000
MSDial	279	279	354	354	pos_1507	pos_704	similar chromatogram in higher mz	$[M+H]^+$	$[M+H]^+$	12.409	12.412	284.0987	189.1228	1.000
Adducts/Neutral losses	279	279	354	18	pos_1507	neg_1402	pol / adduct	$[M+H]^+$	$[M-H]^-$	12.409	12.399	284.0987	282.0844	0.995
Adducts/Neutral losses	279	279	354	18	pos_1507	neg_1402	pol / adduct	$[M+H]^+$	$[M-H]^-$	12.409	12.399	284.0987	282.0844	0.995
Adducts/Neutral losses	279	279	248	29	pos_2070	neg_1668	pol / adduct	$[M+NH_4]^+$	$[M-H]^-$	12.468	12.463	344.1328	325.0927	0.975
MSDial	279	279	29	29	neg_156	neg_1668	similar chromatogram in higher mz	$[M-H]^-$	$[M-H]^-$	12.453	12.463	114.0560	325.0927	1.000
Adducts/Neutral losses	279	279	248	29	pos_2070	neg_1668	pol / adduct	$[M+NH_4]^+$	$[M-H]^-$	12.468	12.463	344.1328	325.0927	0.975
Adducts/Neutral losses	281	281	335	101	pos_3698	neg_2804	pol / adduct	$[M+H]^+$	$[M-H]^-$	11.002	10.987	803.2246	801.2103	0.645
Adducts/Neutral losses	281	281	335	101	pos_3698	neg_2804	pol / adduct	$[M+H]^+$	$[M-H]^-$	11.002	10.987	803.2246	801.2103	0.645

Adducts/Neutral losses	284	284	341	103	pos_3738	neg_2814	pol / adduct	$[M+Na]^+$	$[M-H]^-$	9.510	9.550	855.1955	831.1994	0.552
Adducts/Neutral losses	284	284	341	103	pos_3738	neg_2814	pol / adduct	$[M+Na]^+$	$[M-H]^-$	9.510	9.550	855.1955	831.1994	0.552
Adducts/Neutral losses	284	284	338	103	pos_3722	neg_2814	pol / adduct	$[M+H]^+$	$[M-H]^-$	9.563	9.550	833.2142	831.1994	0.947
Adducts/Neutral losses	284	284	338	103	pos_3722	neg_2814	pol / adduct	$[M+H]^+$	$[M-H]^-$	9.563	9.550	833.2142	831.1994	0.947
Adducts/Neutral losses	285	285	339	102	pos_3730	neg_2810	pol / adduct	$[M+NH_4]^+$	$[M-H]^-$	9.682	9.728	840.4016	821.3619	0.679
Adducts/Neutral losses	285	285	339	102	pos_3730	neg_2810	pol / adduct	$[M+NH_4]^+$	$[M-H]^-$	9.682	9.728	840.4016	821.3619	0.679
Adducts/Neutral losses	286	286	340	107	pos_3733	neg_2821	pol / adduct	$[M+H]^+$	$[M-H]^-$	7.971	7.950	847.2290	845.2169	0.816
Adducts/Neutral losses	286	286	340	107	pos_3733	neg_2821	pol / adduct	$[M+H]^+$	$[M-H]^-$	7.971	7.950	847.2290	845.2169	0.816
MSDial	287	287	400	400	pos_3734	pos_830	similar chromatogram in higher mz	$[M+H]^+$	$[M+H]^+$	6.960	6.926	847.2292	203.1385	1.000
Adducts/Neutral losses	287	287	400	106	pos_3734	neg_2820	pol / adduct	$[M+H]^+$	$[M-H]^-$	6.960	6.965	847.2292	845.2157	0.963
MSDial	287	287	106	106	neg_2124	neg_2820	Pearson correlation	$[M-2H]^{2-}$	$[M-H]^-$	6.958	6.965	422.1035	845.2157	0.953
Adducts/Neutral losses	287	287	400	106	pos_3734	neg_2820	pol / adduct	$[M+H]^+$	$[M-H]^-$	6.960	6.965	847.2292	845.2157	0.963
MSDial	295	295	351	351	pos_414	pos_418	found in higher mz's MsMs	$[M+H]^+$	$[M+H]^+$	9.238	9.241	150.1123	151.0961	1.000
Adducts/Neutral losses	306	306	363	134	pos_490	neg_501	pol / adduct	$[M+H]^+$	$[M-H]^-$	14.909	14.939	162.0757	160.0612	0.961
Adducts/Neutral losses	306	306	363	134	pos_490	neg_501	pol / adduct	$[M+H]^+$	$[M-H]^-$	14.909	14.939	162.0757	160.0612	0.961
MSDial	306	306	363	363	pos_2197	pos_490	similar chromatogram in higher mz	$[M+NH_4]^+$	$[M+H]^+$	14.919	14.909	360.1488	162.0757	1.000
Adducts/Neutral losses	306	306	262	34	pos_2236	neg_1761	pol / adduct	[M+Na]+	$[M-H]^-$	14.909	14.944	365.1050	341.1081	0.866
Adducts/Neutral losses	306	306	363	34	pos_2197	neg_1761	pol / adduct	$[M+NH_4]^+$	$[M-H]^-$	14.919	14.944	360.1488	341.1081	0.995

Adducts/Neutral losses	306	306	363	34	pos_2197	neg_1761	pol / adduct	$[M+NH_4]^+$	$[M-H]^-$	14.919	14.944	360.1488	341.1081	0.995
Adducts/Neutral losses	306	306	262	34	pos_2236	neg_1761	pol / adduct	[M+Na]+	$[M-H]^-$	14.909	14.944	365.1050	341.1081	0.866
MSDial	308	308	365	365	pos_339	pos_510	Pearson correlation	$[M+H]^+$	$[M+H]^+$	17.467	17.475	143.0812	164.1279	0.962
MSDial	309	309	366	366	pos_2071	pos_515	Pearson correlation	$[M+NH_4]^+$	$[M+H]^+$	6.868	6.856	344.1338	165.0541	0.979
MSDial	309	309	366	366	pos_434	pos_515	similar chromatogram in higher mz	$[M+H]^+$	$[M+H]^+$	6.875	6.856	154.0972	165.0541	1.000
MSDial	309	309	366	366	pos_2071	pos_515	found in higher mz's MsMs	$[M+NH_4]^+$	$[M+H]^+$	6.868	6.856	344.1338	165.0541	1.000
Adducts/Neutral losses	312	312	349	145	pos_521	neg_540	pol / adduct	$[M+H]^+$	$[M-H]^-$	9.858	9.858	166.0857	164.0714	0.972
Adducts/Neutral losses	312	312	349	145	pos_521	neg_540	pol / adduct	$[M+H]^+$	$[M-H]^-$	9.858	9.858	166.0857	164.0714	0.972
MSDial	312	312	349	349	pos_209	pos_521	Pearson correlation	$[M+H]^+$	$[M+H]^+$	9.858	9.858	120.0805	166.0857	0.999
MSDial	312	312	349	349	pos_209	pos_521	found in higher mz's MsMs	$[M+H]^+$	$[M+H]^+$	9.858	9.858	120.0805	166.0857	1.000
MSDial	314	314	370	370	pos_1233	pos_410	similar chromatogram in higher mz	$[M+H]^+$	$[M-H_2O+H]^+$	7.404	7.390	252.1089	150.0546	1.000
Adducts/Neutral losses	314	314	370	370	pos_527	pos_410	neutral loss	H_2O	H_2O	7.389	7.390	168.0652	150.0546	1.000
MSDial	314	314	370	370	pos_410	pos_527	found in higher mz's MsMs	$[M-H_2O+H]+$	$[M+H]^+$	7.390	7.389	150.0546	168.0652	1.000
MSDial	314	314	370	370	pos_410	pos_527	pol / adduct	$[M-H_2O+H]^+$	$[M+H]^+$	7.390	7.389	150.0546	168.0652	1.000
MSDial	314	314	370	370	pos_410	pos_527	Pearson correlation	$[M-H_2O+H]^+$	$[M+H]^+$	7.390	7.389	150.0546	168.0652	1.000
Adducts/Neutral losses	320	320	376	154	pos_589	neg_624	pol / adduct	$[M+H]^+$	$[M-H]^-$	27.812	27.805	175.1184	173.1040	0.974
Adducts/Neutral losses	320	320	376	154	pos_589	neg_624	pol / adduct	$[M+H]^+$	$[M-H]^-$	27.812	27.805	175.1184	173.1040	0.974
MSDial	323	323	407	407	pos_2320	pos_609	Pearson correlation	$[M+NH_4]^+$	$[M-H_2O+H]^+$	11.090	11.091	374.1438	177.0542	0.973
MSDial	323	323	407	407	pos_2320	pos_609	similar chromatogram in higher mz	$[M+NH_4]^+$	$[M-H_2O+H]^+$	11.090	11.091	374.1438	177.0542	1.000
MSDial	323	323	407	407	pos_2320	pos_609	found in higher mz's MsMs	$[M+NH_4]^+$	$[M-H_2O+H]^+$	11.090	11.091	374.1438	177.0542	1.000
Adducts/Neutral losses	323	323	407	407	pos_2320	pos_908	neutral loss	$C_6H_{10}O_5$	$C_6H_{10}O_5$	11.090	11.093	374.1438	212.0947	0.236

MSDial	323	323	407	407	pos_609	pos_908	pol / adduct	$[M-H_2O+H]^+$	$[M-H]^-$	11.091	11.093	177.0542	212.0947	1.000
MSDial	323	323	407	407	pos_140	pos_908	Pearson correlation	$[M+H]^+$	$[M-H]^-$	11.104	11.093	104.0704	212.0947	0.921
MSDial	323	323	407	407	pos_609	pos_908	pol / adduct	$[M-H_2O+H]^+$	$[M-H]^-$	11.091	11.093	177.0542	212.0947	1.000
Adducts/Neutral losses	323	323	265	74	pos_2352	neg_1823	pol / adduct	$[M+Na]^+$	$[M-H]^-$	11.110	11.096	379.0986	355.1031	0.763
Adducts/Neutral losses	323	323	265	74	pos_2352	neg_1823	pol / adduct	$[M+Na]^+$	$[M-H]^-$	11.110	11.096	379.0986	355.1031	0.763
Adducts/Neutral losses	323	323	407	74	pos_2320	neg_1823	pol / adduct	$[M+NH_4]^+$	$[M-H]^-$	11.090	11.096	374.1438	355.1031	0.885
Adducts/Neutral losses	323	323	407	74	pos_2320	neg_1823	pol / adduct	$[M+NH_4]^+$	$[M-H]^-$	11.090	11.096	374.1438	355.1031	0.885
MSDial	328	328	385	385	pos_218	pos_304	Pearson correlation	$[M+H]^+$	$[M-H_2O+H]^+$	12.984	12.985	123.0437	136.0756	0.998
Adducts/Neutral losses	328	328	385	164	pos_645	neg_679	pol / adduct	$[M+H]^+$	$[M-H]^-$	12.985	12.982	182.0808	180.0663	0.982
Adducts/Neutral losses	328	328	385	164	pos_645	neg_679	pol / adduct	$[M+H]^+$	$[M-H]^-$	12.985	12.982	182.0808	180.0663	0.982
MSDial	328	328	385	385	pos_304	pos_645	Pearson correlation	$[M-H_2O+H]^+$	$[M+H]^+$	12.985	12.985	136.0756	182.0808	0.999
MSDial	328	328	385	385	pos_218	pos_645	Pearson correlation	$[M+H]^+$	$[M+H]^+$	12.984	12.985	123.0437	182.0808	0.997
MSDial	328	328	385	385	pos_218	pos_645	found in higher mz's MsMs	$[M+H]^+$	$[M+H]^+$	12.984	12.985	123.0437	182.0808	1.000
MSDial	328	328	385	385	pos_304	pos_645	found in higher mz's MsMs	$[M-H_2O+H]^+$	$[M+H]^+$	12.985	12.985	136.0756	182.0808	1.000
Adducts/Neutral losses	330	330	387	167	pos_656	neg_691	pol / adduct	$[M+H]^+$	$[M-H]^-$	4.422	4.420	184.0600	182.0453	0.933
Adducts/Neutral losses	330	330	387	167	pos_656	neg_691	pol / adduct	$[M+H]^+$	$[M-H]^-$	4.422	4.420	184.0600	182.0453	0.933
MSDial	335	335	392	392	pos_3066	pos_715	Pearson correlation	$[M+H]^+$	$[M+H]^+$	6.565	6.518	509.1276	190.0493	0.902
Adducts/Neutral losses	336	336	393	169	pos_717	neg_729	pol / adduct	$[M+H]^+$	$[M-H]^-$	14.437	14.446	190.0706	188.0562	0.860
Adducts/Neutral losses	336	336	393	169	pos_717	neg_729	pol / adduct	$[M+H]^+$	$[M-H]^-$	14.437	14.446	190.0706	188.0562	0.860
MSDial	339	339	396	396	pos_2319	pos_604	Pearson correlation	$[M+NH_4]^+$	$[M-H_2O+H]^+$	6.583	6.584	374.1438	177.0541	0.979

MSDial	339	339	396	396	pos_2739	pos_604	found in higher mz's MsMs	[M+H]$^{+}$	[M-H$_2$O+H]$^{+}$	6.563	6.584	447.1280	177.0541	1.000
MSDial	339	339	396	396	pos_2319	pos_604	found in higher mz's MsMs	[M+NH$_4$]$^{+}$	[M-H$_2$O+H]$^{+}$	6.583	6.584	374.1438	177.0541	1.000
Adducts/Neutral losses	339	339	396	396	pos_755	pos_604	neutral loss	H$_2$O	H$_2$O	6.583	6.584	195.0649	177.0541	0.987
Adducts/Neutral losses	339	339	396	177	pos_755	neg_770	pol / adduct	[M+H]$^{+}$	[M-H]$^{-}$	6.583	6.574	195.0649	193.0502	0.948
Adducts/Neutral losses	339	339	396	177	pos_755	neg_770	pol / adduct	[M+H]$^{+}$	[M-H]$^{-}$	6.583	6.574	195.0649	193.0502	0.948
MSDial	339	339	396	396	pos_2319	pos_755	found in higher mz's MsMs	[M+NH$_4$]$^{+}$	[M+H]$^{+}$	6.583	6.583	374.1438	195.0649	1.000
MSDial	339	339	396	396	pos_604	pos_755	found in higher mz's MsMs	[M-H$_2$O+H]$^{+}$	[M+H]$^{+}$	6.584	6.583	177.0541	195.0649	1.000
MSDial	339	339	396	396	pos_2319	pos_755	Pearson correlation	[M+NH$_4$]$^{+}$	[M+H]$^{+}$	6.583	6.583	374.1438	195.0649	0.965
MSDial	339	339	396	396	pos_604	pos_755	Pearson correlation	[M-H$_2$O+H]$^{+}$	[M+H]$^{+}$	6.584	6.583	177.0541	195.0649	0.987
MSDial	339	339	396	396	pos_604	pos_755	pol / adduct	[M-H$_2$O+H]$^{+}$	[M+H]$^{+}$	6.584	6.583	177.0541	195.0649	1.000
Adducts/Neutral losses	340	340	397	179	pos_756	neg_774	pol / adduct	[M+H]$^{+}$	[M-H]$^{-}$	5.285	5.300	195.0649	193.0503	0.879
Adducts/Neutral losses	340	340	397	179	pos_756	neg_774	pol / adduct	[M+H]$^{+}$	[M-H]$^{-}$	5.285	5.300	195.0649	193.0503	0.879
Adducts/Neutral losses	341	341	398	178	pos_757	neg_771	pol / adduct	[M+H]$^{+}$	[M-H]$^{-}$	6.909	6.909	195.0649	193.0503	0.949
Adducts/Neutral losses	341	341	398	178	pos_757	neg_771	pol / adduct	[M+H]$^{+}$	[M-H]$^{-}$	6.909	6.909	195.0649	193.0503	0.949
MSDial	343	343	345	345	pos_392	pos_80	found in higher mz's MsMs	[M+H]$^{+}$	[M+H]$^{+}$	15.027	15.028	147.0763	84.0443	1.000
MSDial	343	343	345	345	pos_392	pos_80	Pearson correlation	[M+H]$^{+}$	[M+H]$^{+}$	15.027	15.028	147.0763	84.0443	0.989
Adducts/Neutral losses	345	345	402	184	pos_849	neg_854	pol / adduct	[M+H]$^{+}$	[M-H]$^{-}$	11.447	11.439	205.0968	203.0822	0.991
Adducts/Neutral losses	345	345	402	184	pos_849	neg_854	pol / adduct	[M+H]$^{+}$	[M-H]$^{-}$	11.447	11.439	205.0968	203.0822	0.991
MSDial	347	347	405	405	pos_148	pos_875	found in higher mz's MsMs	[M]$^{+}$	[2M+H]$^{+}$	21.606	21.597	104.1067	207.2059	1.000
MSDial	347	347	405	405	pos_148	pos_875	pol / adduct	[M]$^{+}$	[2M+H]$^{+}$	21.606	21.597	104.1067	207.2059	1.000
MSDial	348	348	406	406	pos_275	pos_88	found in higher mz's MsMs	[M+H]$^{+}$	[M+H]$^{+}$	10.383	10.386	132.1016	86.0963	1.000

MSDial	348	348	406	406	pos_275	pos_88	similar chromato-gram in higher mz	$[M+H]^+$	$[M+H]^+$	10.383	10.386	132.1016	86.0963	1.000
MSDial	348	348	406	406	pos_275	pos_88	Pearson correlation	$[M+H]^+$	$[M+H]^+$	10.383	10.386	132.1016	86.0963	0.993
Adducts/Neutral losses	348	348	406	86	pos_275	neg_266	pol / adduct	$[M+H]^+$	$[M-H]^-$	10.383	10.393	132.1016	130.0872	0.960
Adducts/Neutral losses	348	348	406	86	pos_275	neg_266	pol / adduct	$[M+H]^+$	$[M-H]^-$	10.383	10.393	132.1016	130.0872	0.960
Adducts/Neutral losses	356	356	415	195	pos_976	neg_982	pol / adduct	$[M+H]^+$	$[M-H]^-$	8.253	8.262	220.1177	218.1028	0.943
Adducts/Neutral losses	356	356	415	195	pos_976	neg_982	pol / adduct	$[M+H]^+$	$[M-H]^-$	8.253	8.262	220.1177	218.1028	0.943

Table S2.b. List of features in both positive and negative ionizations mode corresponding to clusters and their association and correlation measured by RPLC-HRMS.

Source	Cluster 1	Cluster 2	Cluster MSdial 1	Cluster MSdial 2	Cpd ID 1	Cpd ID 2	Simple Nature	Adduct 1	Adduct 2	RT 1	RT 2	Mass 1	Mass 2	Correlation
MSDial	25	25	28	28	neg_1086	neg_1214	found in higher mz's MSMS	$[M-H]^-$	$[M+Cl]^-$	3.361	3.366	373.1397	409.1162	1.000
MSDial	25	25	28	28	neg_1086	neg_1214	pol / adduct	$[M-H]^-$	$[M+Cl]^-$	3.361	3.366	373.1397	409.1162	1.000
MSDial	25	25	28	28	neg_1214	neg_580	similar chromatogram in higher mz	$[M+Cl]^-$	$[M-H]^-$	3.366	3.356	409.1162	262.0714	1.000
Adducts/Neutral losses	35	35	409	38	pos_2838	neg_1170	pol / adduct	$[M+Na]^+$	$[M-H]^-$	3.320	3.321	417.1711	393.1762	0.924
Adducts/Neutral losses	35	35	38	38	neg_1316	neg_1170	neutral loss	CH_2O_2	CH_2O_2	3.319	3.321	439.1810	393.1762	0.960
MSDial	35	35	38	38	neg_1170	neg_1316	found in higher mz's MSMS	$[M-H]^-$	$[M-H]^-$	3.321	3.319	393.1762	439.1810	1.000
Adducts/Neutral losses	35	35	409	38	pos_2838	neg_1316	pol / adduct	$[M+Na]^+$	$[M+FA-H]^-$	3.320	3.319	417.1711	439.1810	0.956
MSDial	35	35	38	38	neg_1170	neg_1316	Pearson correlation	$[M-H]^-$	$[M-H]^-$	3.321	3.319	393.1762	439.1810	0.960
MSDial	35	35	38	38	neg_1170	neg_1316	similar chromatogram in higher mz	$[M-H]^-$	$[M-H]^-$	3.321	3.319	393.1762	439.1810	1.000
MSDial	35	35	38	38	neg_1316	neg_1364	similar chromatogram in higher mz	$[M-H]^-$	$[M-H]^-$	3.319	3.321	439.1810	456.1714	1.000
MSDial	35	35	38	38	neg_1170	neg_1364	similar chromatogram in higher mz	$[M-H]^-$	$[M-H]^-$	3.321	3.321	393.1762	456.1714	1.000
MSDial	35	35	38	38	neg_1170	neg_1451	similar chromatogram in higher mz	$[M-H]^-$	$[M-H]^-$	3.321	3.319	393.1762	491.1758	1.000
Adducts/Neutral losses	39	39	402	42	pos_2738	neg_1288	pol / adduct	$[M+NH_4]^+$	$[M+FA-H]^-$	3.011	2.985	404.2268	431.1911	0.931
MSDial	39	39	42	42	neg_1288	neg_1344	Pearson correlation	$[M-H]^-$	$[M-H]^-$	2.985	2.983	431.1911	448.1816	0.929
MSDial	47	47	51	51	neg_1354	neg_1423	Pearson correlation	$[M-H]^-$	$[M-H]^-$	5.566	5.586	452.2775	478.2930	0.942
MSDial	47	47	51	51	neg_1354	neg_1573	similar chromatogram in higher mz	$[M-H]^-$	$[M-H]^-$	5.566	5.568	452.2775	537.3269	1.000
Adducts/Neutral losses	61	61	433	68	pos_3190	neg_1335	pol / adduct	$[M+NH_4]^+$	$[M-H]^-$	5.226	5.231	464.2765	445.2351	0.981
MSDial	61	61	68	68	neg_1335	neg_1518	Pearson correlation	$[M-H]^-$	$[M-H]^-$	5.231	5.224	445.2351	513.2222	0.971
Adducts/Neutral losses	63	63	445	71	pos_3398	neg_1459	pol / adduct	$[M+H]^+$	$[M-H]^-$	3.422	3.418	495.1121	493.0980	0.980

Adducts/Neutral losses	63	63	445	71	pos_3398	neg_1546	pol / adduct	$[M+H]^+$	$[M+Cl]^-$	3.422	3.419	495.1121	529.0745	0.967
MSDial	63	63	71	71	neg_1459	neg_1546	pol / adduct	$[M-H]^-$	$[M+Cl]^-$	3.418	3.419	493.0980	529.0745	1.000
MSDial	63	63	71	71	neg_1459	neg_1546	found in higher mz's MSMS	$[M-H]^-$	$[M+Cl]^-$	3.418	3.419	493.0980	529.0745	1.000
MSDial	63	63	71	71	neg_1459	neg_1546	Pearson correlation	$[M-H]^-$	$[M+Cl]^-$	3.418	3.419	493.0980	529.0745	0.976
MSDial	76	76	90	90	neg_1221	neg_1389	Pearson correlation	$[M-H]^-$	$[M-H]^-$	3.041	3.080	411.1321	465.1422	0.932
Adducts/Neutral losses	76	76	90	90	neg_1640	neg_1635	neutral loss	hydrog./dehydr.	hydrog./dehydro.	3.035	3.058	563.2328	561.2175	0.857
MSDial	76	76	90	90	neg_1635	neg_1640	pol / adduct	$[M+Cl]^-$	$[M+K-2H]^-$	3.058	3.035	561.2175	563.2328	1.000
MSDial	76	76	90	90	neg_1389	neg_1640	Pearson correlation	$[M-H]^-$	$[M+K-2H]^-$	3.080	3.035	465.1422	563.2328	0.941
MSDial	76	76	90	90	neg_1635	neg_223	similar chromatogram in higher mz	$[M+Cl]^-$	$[M-H]^-$	3.058	3.055	561.2175	167.0345	1.000
Adducts/Neutral losses	81	81	482	60	pos_3736	neg_1591	pol / adduct	$[M+Na]^+$	$[M-H]^-$	4.318	4.319	569.1260	545.1292	0.767
MSDial	81	81	60	60	neg_1591	neg_1673	Pearson correlation	$[M-H]^-$	$[M+Cl]^-$	4.319	4.322	545.1292	581.1059	0.910
MSDial	81	81	60	60	neg_1591	neg_1673	pol / adduct	$[M-H]^-$	$[M+Cl]^-$	4.319	4.322	545.1292	581.1059	1.000
Adducts/Neutral losses	81	81	482	60	pos_3736	neg_1673	pol / adduct	[M+Na]+	$[M+Cl]^-$	4.318	4.322	569.1260	581.1059	0.816
MSDial	90	90	110	110	neg_1416	neg_1757	Pearson correlation	$[M+K-2H]^-$	$[M-H]^-$	3.395	3.442	475.2174	631.2231	0.932
MSDial	91	91	111	111	neg_1371	neg_1419	similar chromatogram in higher mz	$[M-H]^-$	$[M-H]^-$	5.469	5.467	458.4209	476.2773	1.000
Adducts/Neutral losses	91	91	456	111	pos_3285	neg_1419	pol / adduct	$[M+H]^+$	$[M-H]^-$	5.463	5.467	478.2916	476.2773	0.992
MSDial	91	91	111	111	neg_1419	neg_1575	found in higher mz's MSMS	$[M-H]^-$	$[M-H]^-$	5.467	5.447	476.2773	538.3138	1.000
MSDial	91	91	111	111	neg_1419	neg_1575	similar chromatogram in higher mz	$[M-H]^-$	$[M-H]^-$	5.467	5.447	476.2773	538.3138	1.000
MSDial	91	91	111	111	neg_1419	neg_1590	found in higher mz's MSMS	$[M-H]^-$	$[M-H]^-$	5.467	5.467	476.2773	544.2648	1.000
MSDial	91	91	111	111	neg_1563	neg_1590	Pearson correlation	$[M-H]^-$	$[M-H]^-$	5.467	5.467	534.2360	544.2648	0.955
MSDial	91	91	111	111	neg_1371	neg_1590	similar chromatogram in higher mz	$[M-H]^-$	$[M-H]^-$	5.469	5.467	458.4209	544.2648	1.000
MSDial	91	91	111	111	neg_1419	neg_1590	Pearson correlation	$[M-H]^-$	$[M-H]^-$	5.467	5.467	476.2773	544.2648	0.969
MSDial	91	91	111	111	neg_1563	neg_1618	Pearson correlation	$[M-H]^-$	$[M-H_2O-H]^-$	5.467	5.505	534.2360	554.3007	0.924

Adducts/Neutral losses	91	91	456	111	pos_3526	neg_1618	pol / adduct	$[M+H]^+$	$[M+Cl]^-$	5.502	5.505	520.3386	554.3007	0.964
MSDial	91	91	111	111	neg_1590	neg_1618	Pearson correlation	$[M-H]^-$	$[M-H_2O-H]^-$	5.467	5.505	544.2648	554.3007	0.904
MSDial	91	91	111	111	neg_1563	neg_1644	Pearson correlation	$[M-H]^-$	$[M-H]^-$	5.467	5.506	534.2360	564.3295	0.910
MSDial	91	91	111	111	neg_1618	neg_1644	Pearson correlation	$[M-H_2O-H]^-$	$[M-H]^-$	5.505	5.506	554.3007	564.3295	0.985
Adducts/Neutral losses	91	91	456	111	pos_3526	neg_1644	pol / adduct	$[M+H]^+$	$[M+FA-H]^-$	5.502	5.506	520.3386	564.3295	0.968
MSDial	91	91	111	111	neg_1419	neg_1644	Pearson correlation	$[M-H]^-$	$[M-H]^-$	5.467	5.506	476.2773	564.3295	0.903
MSDial	91	91	111	111	neg_1590	neg_1644	Pearson correlation	$[M-H]^-$	$[M-H]^-$	5.467	5.506	544.2648	564.3295	0.914
MSDial	91	91	111	111	neg_1644	neg_1739	Pearson correlation	$[M-H]^-$	$[M-H]^-$	5.506	5.506	564.3295	622.2879	0.946
MSDial	91	91	111	111	neg_1618	neg_1739	Pearson correlation	$[M-H_2O-H]^-$	$[M-H]^-$	5.505	5.506	554.3007	622.2879	0.952
MSDial	91	91	111	111	neg_1618	neg_1759	Pearson correlation	$[M-H_2O-H]^-$	$[M-H]^-$	5.505	5.506	554.3007	632.3168	0.985
MSDial	91	91	111	111	neg_1563	neg_1759	Pearson correlation	$[M-H]^-$	$[M-H]^-$	5.467	5.506	534.2360	632.3168	0.911
MSDial	91	91	111	111	neg_1644	neg_1759	similar chromatogram in higher mz	$[M-H]^-$	$[M-H]^-$	5.506	5.506	564.3295	632.3168	1.000
MSDial	91	91	111	111	neg_1739	neg_1759	Pearson correlation	$[M-H]^-$	$[M-H]^-$	5.506	5.506	622.2879	632.3168	0.967
MSDial	91	91	111	111	neg_1644	neg_1759	Pearson correlation	$[M-H]^-$	$[M-H]^-$	5.506	5.506	564.3295	632.3168	0.984
Adducts/Neutral losses	91	91	456	456	pos_3526	pos_3285	neutral loss	C_3H_6	C_3H_6	5.502	5.463	520.3386	478.2916	0.912
MSDial	91	91	456	456	pos_3285	pos_3526	Pearson correlation	$[M+H]^+$	$[[M+H]^+$	5.463	5.502	478.2916	520.3386	0.912
MSDial	94	94	114	114	neg_1351	neg_1765	Pearson correlation	$[M-H]^-$	$[M-H]^-$	3.425	3.428	451.1633	639.1555	0.925
MSDial	94	94	114	114	neg_1351	neg_1765	similar chromatogram in higher mz	$[M-H]^-$	$[M-H]^-$	3.425	3.428	451.1633	639.1555	1.000
Adducts/Neutral losses	99	99	498	122	pos_3936	neg_1772	pol / adduct	$[M+Na]^+$	[M+Cl]-	4.161	4.170	631.3428	643.3239	0.872
Adducts/Neutral losses	99	99	498	122	pos_3796	neg_1772	pol / adduct	$[M+H-H_2O]^+$	[M+Cl]-	4.166	4.170	591.3522	643.3239	0.876
Adducts/Neutral losses	99	99	498	122	pos_3796	neg_1785	pol / adduct	$[M+H-H_2O]^+$	$[M+FA-H]^-$	4.166	4.168	591.3522	653.3526	0.952
MSDial	99	99	122	122	neg_1772	neg_1785	Pearson correlation	$[M-H]^-$	$[M-H_2O-H]^-$	4.170	4.168	643.3239	653.3526	0.926

Adducts/Neutral losses	99	99	498	122	pos_3936	neg_1785	pol / adduct	[M+Na]$^{+}$	[M+FA-H]-	4.161	4.168	631.3428	653.3526	0.871
MSDial	99	99	498	498	pos_3796	pos_3936	pol / adduct	[M+H-H_2O]$^{+}$	[M+Na]$^{+}$	4.166	4.161	591.3522	631.3428	1.000
MSDial	100	100	123	123	neg_1779	neg_1786	similar chromatogram in higher mz	[M-2H]$^{2-}$	[M-2H]$^{2-}$	4.566	4.574	647.2724	654.2805	1.000
MSDial	101	101	124	124	neg_1775	neg_1790	Pearson correlation	[M-H]$^{-}$	[M-H]$^{-}$	4.098	4.099	645.3398	655.3685	0.929
Adducts/Neutral losses	102	102	489	126	pos_3857	neg_1691	pol / adduct	[M+NH_4]$^{+}$	[M-H]$^{-}$	3.338	3.341	610.2682	591.2281	0.876
MSDial	102	102	126	126	neg_1691	neg_1792	found in higher mz's MSMS	[M-H]$^{-}$	[M-H]$^{-}$	3.341	3.333	591.2281	659.2154	1.000
MSDial	105	105	129	129	neg_1267	neg_1802	similar chromatogram in higher mz	[M-H]$^{-}$	[M-H]$^{-}$	5.421	5.432	423.2509	664.3817	1.000
Adducts/Neutral losses	106	106	488	131	pos_3854	neg_1798	pol / adduct	[M+H-H_2O]$^{+}$	[M+Cl]-	3.780	3.782	609.3611	661.3354	0.931
Adducts/Neutral losses	106	106	488	131	pos_3854	neg_1806	pol / adduct	[M+H-H_2O]$^{+}$	[M+FA-H]-	3.780	3.778	609.3611	671.3637	0.941
MSDial	106	106	131	131	neg_1798	neg_1806	Pearson correlation	[M-H]$^{-}$	[M-H]$^{-}$	3.782	3.778	661.3354	671.3637	0.941
Adducts/Neutral losses	108	108	138	138	neg_1846	neg_1787	neutral loss	CH_2O_2	CH_2O_2	2.770	2.767	701.1556	655.1501	0.909
Adducts/Neutral losses	108	108	515	138	pos_4026	neg_1787	pol / adduct	[M+H]$^{+}$	[M-H]$^{-}$	2.767	2.767	657.1633	655.1501	0.962
Adducts/Neutral losses	108	108	515	138	pos_4093	neg_1787	pol / adduct	[M+Na]$^{+}$	[M-H]$^{-}$	2.768	2.767	679.1463	655.1501	0.864
MSDial	108	108	138	138	neg_1787	neg_1829	pol / adduct	[M-H]$^{-}$	[M+Cl]-	2.767	2.770	655.1501	691.1266	1.000
Adducts/Neutral losses	108	108	515	138	pos_4093	neg_1829	pol / adduct	[M+Na]$^{+}$	[M+Cl]-	2.768	2.770	679.1463	691.1266	0.876
MSDial	108	108	138	138	neg_1787	neg_1829	Pearson correlation	[M-H]$^{-}$	[M+Cl]-	2.767	2.770	655.1501	691.1266	0.932
Adducts/Neutral losses	108	108	515	138	pos_4026	neg_1829	pol / adduct	[M+H]$^{+}$	[M+Cl]-	2.767	2.770	657.1633	691.1266	0.960
MSDial	108	108	138	138	neg_1787	neg_1829	found in higher mz's MSMS	[M-H]-	[M+Cl]-	2.767	2.770	655.1501	691.1266	1.000
Adducts/Neutral losses	108	108	515	138	pos_4093	neg_1846	pol / adduct	[M+Na]$^{+}$	[M+FA-H]$^{-}$	2.768	2.770	679.1463	701.1556	0.726
Adducts/Neutral losses	108	108	515	138	pos_4026	neg_1846	pol / adduct	[M+H]$^{+}$	[M+FA-H]$^{-}$	2.767	2.770	657.1633	701.1556	0.862

MSDial	108	108	138	138	neg_1787	neg_1846	found in higher mz's MSMS	[M-H]$^-$	[M-H]$^-$	2.767	2.770	655.1501	701.1556	1.000
MSDial	108	108	138	138	neg_1787	neg_1846	Pearson correlation	[M-H]$^-$	[M-H]$^-$	2.767	2.770	655.1501	701.1556	0.909
Adducts/Neutral losses	108	108	138	138	neg_1888	neg_1846	neutral loss	CO_2	CO_2	2.766	2.770	745.1456	701.1556	0.765
MSDial	108	108	138	138	neg_1829	neg_1888	Pearson correlation	[M+Cl]$^-$	[M-H]$^-$	2.770	2.766	691.1266	745.1456	0.927
MSDial	108	108	138	138	neg_1787	neg_1888	found in higher mz's MSMS	[M-H]$^-$	[M-H]$^-$	2.767	2.766	655.1501	745.1456	1.000
MSDial	108	108	515	515	pos_4026	pos_4093	pol / adduct	[M+H]$^+$	[M+Na]$^+$	2.767	2.768	657.1633	679.1463	1.000
MSDial	108	108	515	515	pos_4026	pos_4093	Pearson correlation	[M+H]$^+$	[M+Na]$^+$	2.767	2.768	657.1633	679.1463	0.903
Adducts/Neutral losses	110	110	508	140	pos_4060	neg_1826	pol / adduct	[M+NH$_4$]$^+$	[M+Cl]-	5.054	5.051	666.3666	683.3033	0.979
MSDial	110	110	140	140	neg_1826	neg_1836	Pearson correlation	[M-H]$^-$	[M-H]$^-$	5.051	5.052	683.3033	693.3324	0.993
Adducts/Neutral losses	110	110	508	140	pos_4060	neg_1836	pol / adduct	[M+NH$_4$]$^+$	[M+FA-H]-	5.054	5.052	666.3666	693.3324	0.981
MSDial	112	112	144	144	neg_1722	neg_1843	Pearson correlation	[M-H]$^-$	[M-H]$^-$	3.284	3.245	611.2543	699.2705	0.909
Adducts/Neutral losses	113	113	513	145	pos_4090	neg_1828	pol / adduct	[M+Na]$^+$	[M+Cl]$^-$	5.492	5.489	677.3709	689.3510	0.958
Adducts/Neutral losses	113	113	513	145	pos_4074	neg_1828	pol / adduct	[M+NH$_4$]$^+$	[M+Cl]$^-$	5.494	5.489	672.4153	689.3510	0.965
Adducts/Neutral losses	113	113	513	145	pos_4074	neg_1844	pol / adduct	[M+NH$_4$]$^+$	[M+FA-H]$^-$	5.494	5.489	672.4153	699.3792	0.990
MSDial	113	113	145	145	neg_1828	neg_1844	Pearson correlation	[M-H]$^-$	[M-H]$^-$	5.489	5.489	689.3510	699.3792	0.985
Adducts/Neutral losses	113	113	513	145	pos_4090	neg_1844	pol / adduct	[M+Na]$^+$	[M+FA-H]$^-$	5.492	5.489	677.3709	699.3792	0.985
MSDial	113	113	513	513	pos_4074	pos_4090	Pearson correlation	[M+H]$^+$	[M+H]$^+$	5.494	5.492	672.4153	677.3709	0.994
MSDial	114	114	136	136	neg_1845	neg_1849	Pearson correlation	[M-H]$^-$	[M-2H]$^{2-}$	4.567	4.566	700.2820	705.2961	0.922
MSDial	114	114	136	136	neg_1823	neg_1849	Pearson correlation	[M-2H]$^{2-}$	[M-2H]$^{2-}$	4.567	4.566	682.2891	705.2961	0.916
Adducts/Neutral losses	116	116	148	148	neg_1893	neg_1809	neutral loss	malonyl-H_2O	malonyl-H_2O	4.167	4.147	759.2867	673.2862	0.908
MSDial	116	116	148	148	neg_1809	neg_1854	Pearson correlation	[M-H]$^-$	[M+Cl]$^-$	4.147	4.146	673.2862	709.2632	0.945

MSDial	116	116	148	148	neg_1809	neg_1854	pol / adduct	[M-H]$^-$	[M+Cl]$^-$	4.147	4.146	673.2862	709.2632	1.000
MSDial	116	116	148	148	neg_1809	neg_1893	Pearson correlation	[M-H]$^-$	[M-H]$^-$	4.147	4.167	673.2862	759.2867	0.908
MSDial	116	116	148	148	neg_1854	neg_1893	Pearson correlation	[M+Cl]$^-$	[M-H]$^-$	4.146	4.167	709.2632	759.2867	0.932
MSDial	119	119	146	146	neg_1847	neg_1873	Pearson correlation	[M-H]$^-$	[M-2H]$^{2-}$	4.766	4.766	703.2950	726.3012	0.949
MSDial	121	121	157	157	neg_1866	neg_1887	Pearson correlation	[M-H]$^-$	[M-2H]$^{2-}$	4.615	4.617	718.3001	741.3069	0.972
Adducts/Neutral losses	124	124	514	161	pos_4091	neg_1859	pol / adduct	[M+H]$^+$	[M+Cl]$^-$	5.285	5.284	677.3749	711.3356	0.961
MSDial	124	124	161	161	neg_1859	neg_1868	pol / adduct	[M+Cl]$^-$	[M+FA-H]$^-$	5.284	5.284	711.3356	721.3637	1.000
MSDial	124	124	161	161	neg_1859	neg_1868	Pearson correlation	[M+Cl]$^-$	[M+FA-H]$^-$	5.284	5.284	711.3356	721.3637	0.988
Adducts/Neutral losses	124	124	514	161	pos_4091	neg_1868	pol / adduct	[M+H]$^+$	[M+FA-H]$^-$	5.285	5.284	677.3749	721.3637	0.984
Adducts/Neutral losses	124	124	161	161	neg_1895	neg_1868	neutral loss	CO_2	CO_2	5.285	5.284	765.3526	721.3637	0.990
MSDial	124	124	161	161	neg_1868	neg_1885	Pearson correlation	[M+FA-H]$^-$	[M-H]$^-$	5.284	5.278	721.3637	738.3546	0.963
MSDial	124	124	161	161	neg_1859	neg_1885	Pearson correlation	[M+Cl]$^-$	[M-H]$^-$	5.284	5.278	711.3356	738.3546	0.967
MSDial	124	124	161	161	neg_1868	neg_1895	Pearson correlation	[M+FA-H]$^-$	[M+Cl]$^-$	5.284	5.285	721.3637	765.3526	0.990
MSDial	124	124	161	161	neg_1859	neg_1895	Pearson correlation	[M+Cl]$^-$	[M+Cl]$^-$	5.284	5.285	711.3356	765.3526	0.987
MSDial	124	124	161	161	neg_1859	neg_1895	pol / adduct	[M+Cl]$^-$	[M+Cl]$^-$	5.284	5.285	711.3356	765.3526	1.000
MSDial	124	124	161	161	neg_1885	neg_1895	Pearson correlation	[M-H]$^-$	[M+Cl]$^-$	5.278	5.285	738.3546	765.3526	0.955
MSDial	124	124	161	161	neg_1868	neg_1901	pol / adduct	[M+FA-H]$^-$	[M+Cl]$^-$	5.284	5.280	721.3637	775.2818	1.000
MSDial	124	124	161	161	neg_1885	neg_1909	Pearson correlation	[M-H]-	[M-H]$^-$	5.278	5.283	738.3546	789.3508	0.961
MSDial	124	124	161	161	neg_1868	neg_1909	Pearson correlation	[M+FA-H]$^-$	[M-H]$^-$	5.284	5.283	721.3637	789.3508	0.975
MSDial	124	124	161	161	neg_1859	neg_1909	Pearson correlation	[M+Cl]$^-$	[M-H]$^-$	5.284	5.283	711.3356	789.3508	0.990
MSDial	124	124	161	161	neg_1895	neg_1909	Pearson correlation	[M+Cl]$^-$	[M-H]$^-$	5.285	5.283	765.3526	789.3508	0.972
MSDial	127	127	166	166	neg_1855	neg_1905	similar chromatogram in higher mz	[M-H]$^-$	[M-H]$^-$	3.888	3.876	709.3430	785.3949	1.000
MSDial	127	127	166	166	neg_1605	neg_1905	similar chromatogram in higher mz	[M-H]$^-$	[M-H]$^-$	3.872	3.876	551.2335	785.3949	1.000
MSDial	127	127	166	166	neg_1039	neg_1905	similar chromatogram in higher mz	[M-H]$^-$	[M-H]$^-$	3.873	3.876	361.1863	785.3949	1.000

Adducts/Neutral losses	131	131	221	221	neg_1373	neg_1194	neutral loss	$C_2H_2O_2$	$C_2H_2O_2$	2.885	2.857	459.1502	401.1444	0.905
Adducts/Neutral losses	131	131	413	221	pos_2731	neg_1194	pol / adduct	$[M+H]^+$	$[M-H]^-$	2.840	2.857	403.1576	401.1444	0.895
Adducts/Neutral losses	131	131	221	221	neg_1340	neg_1194	neutral loss	CH_2O_2	CH_2O_2	2.857	2.857	447.1499	401.1444	0.967
Adducts/Neutral losses	131	131	413	221	pos_2692	neg_1272	pol / adduct	$[M+NH_4]^+$	$[M+FA-H]^-$	2.838	2.842	398.2018	425.1657	0.940
MSDial	131	131	221	221	neg_1194	neg_1272	Pearson correlation	$[M-H]^-$	$[M+FA-H]^-$	2.857	2.842	401.1444	425.1657	0.913
Adducts/Neutral losses	131	131	221	221	neg_1363	neg_1272	neutral loss	CH_2O	CH_2O	2.830	2.842	455.1764	425.1657	0.921
MSDial	131	131	221	221	neg_1194	neg_1307	Pearson correlation	$[M-H]^-$	$[M+Cl]^-$	2.857	2.857	401.1444	437.1210	0.922
Adducts/Neutral losses	131	131	413	221	pos_2731	neg_1307	pol / adduct	$[M+H]^+$	$[M+Cl]^-$	2.840	2.857	403.1576	437.1210	0.861
MSDial	131	131	221	221	neg_1194	neg_1307	found in higher mz's MSMS	$[M-H]^-$	$[M+Cl]^-$	2.857	2.857	401.1444	437.1210	1.000
MSDial	131	131	221	221	neg_1272	neg_1340	Pearson correlation	$[M+FA-H]^-$	$[M+FA-H]^-$	2.842	2.857	425.1657	447.1499	0.927
Adducts/Neutral losses	131	131	413	221	pos_2731	neg_1340	pol / adduct	$[M+H]^+$	$[M+FA-H]^-$	2.840	2.857	403.1576	447.1499	0.905
MSDial	131	131	221	221	neg_1194	neg_1340	Pearson correlation	[M-H]-	$[M+FA-H]^-$	2.857	2.857	401.1444	447.1499	0.967
MSDial	131	131	221	221	neg_1307	neg_1340	Pearson correlation	$[M+Cl]^-$	$[M+FA-H]^-$	2.857	2.857	437.1210	447.1499	0.932
MSDial	131	131	221	221	neg_1307	neg_1340	pol / adduct	$[M+Cl]^-$	$[M+FA-H]^-$	2.857	2.857	437.1210	447.1499	1.000
MSDial	131	131	221	221	neg_1194	neg_1340	found in higher mz's MSMS	[M-H]-	$[M+FA-H]^-$	2.857	2.857	401.1444	447.1499	1.000
MSDial	131	131	221	221	neg_1272	neg_1363	Pearson correlation	$[M+FA-H]^-$	$[M-H]^-$	2.842	2.830	425.1657	455.1764	0.921
MSDial	131	131	221	221	neg_1194	neg_1373	Pearson correlation	$[M-H]^-$	$[M-H]^-$	2.857	2.885	401.1444	459.1502	0.905
MSDial	131	131	221	221	neg_1272	neg_1373	Pearson correlation	$[M+FA-H]^-$	$[M-H]^-$	2.842	2.885	425.1657	459.1502	0.957
MSDial	131	131	221	221	neg_1340	neg_1373	Pearson correlation	$[M+FA-H]^-$	$[M-H]^-$	2.857	2.885	447.1499	459.1502	0.922
MSDial	131	131	221	221	neg_1229	neg_1392	Pearson correlation	$[M+Na-2H]^-$	$[M-H]^-$	2.792	2.787	413.0613	467.0797	0.908
MSDial	131	131	221	221	neg_1392	neg_1438	Pearson correlation	$[M-H]^-$	$[M+Na-2H]^-$	2.787	2.794	467.0797	485.0601	0.921
MSDial	131	131	221	221	neg_1229	neg_1438	Pearson correlation	[M+Na-2H]-	$[M+Na-2H]^-$	2.792	2.794	413.0613	485.0601	0.944

MSDial	131	131	221	221	neg_1194	neg_1753	similar chromatogram in higher mz	[M-H]-	[M+K-2H]-	2.857	2.861	401.1444	629.2109	1.000
Adducts/Neutral losses	131	131	531	221	pos_4206	neg_1882	pol / adduct	$[M+NH_4]^+$	[M+Na-2H]-	2.786	2.783	730.2524	733.1951	0.628
MSDial	131	131	221	221	neg_1780	neg_192	similar chromatogram in higher mz	[M-H]-	[M-H]-	2.795	2.801	649.1873	159.0659	1.000
MSDial	131	131	221	221	neg_1392	neg_312	found in higher mz's MSMS	[M-H]-	[M-H]-	2.787	2.789	467.0797	193.0502	1.000
MSDial	131	131	221	221	neg_1438	neg_312	found in higher mz's MSMS	[M+Na-2H]-	[M-H]-	2.794	2.789	485.0601	193.0502	1.000
MSDial	131	131	221	221	neg_1780	neg_312	found in higher mz's MSMS	[M-H]-	[M-H]-	2.795	2.789	649.1873	193.0502	1.000
MSDial	131	131	221	221	neg_1882	neg_312	found in higher mz's MSMS	[M+Na-2H]-	[M-H]-	2.783	2.789	733.1951	193.0502	1.000
MSDial	131	131	221	221	neg_1272	neg_538	Pearson correlation	[M+FA-H]-	[M-H]-	2.842	2.802	425.1657	249.0875	0.921
MSDial	131	131	221	221	neg_1363	neg_538	Pearson correlation	[M-H]-	[M-H]-	2.830	2.802	455.1764	249.0875	0.920
MSDial	131	131	221	221	neg_312	neg_538	Pearson correlation	[M-H]-	[M-H]-	2.789	2.802	193.0502	249.0875	0.907
MSDial	131	131	413	413	pos_1257	pos_2478	Pearson correlation	[M+H]+	$[M+NH_4]^+$	2.818	2.795	263.1017	374.1438	0.902
MSDial	131	131	413	413	pos_2478	pos_2519	pol / adduct	$[M+NH_4]^+$	[M+Na]+	2.795	2.784	374.1438	379.0985	1.000
MSDial	131	131	413	413	pos_2478	pos_2519	Pearson correlation	$[M+NH_4]^+$	[M+Na]+	2.795	2.784	374.1438	379.0985	0.968
MSDial	131	131	413	413	pos_2519	pos_2664	pol / adduct	[M+Na]+	[M+K]+	2.784	2.788	379.0985	395.0724	1.000
MSDial	131	131	413	413	pos_2478	pos_2664	pol / adduct	$[M+NH_4]^+$	[M+K]+	2.795	2.788	374.1438	395.0724	1.000
MSDial	131	131	413	413	pos_1331	pos_2664	Pearson correlation	$[M+H]^{2+}$	[M+K]+	2.789	2.788	269.5735	395.0724	0.921
MSDial	131	131	413	413	pos_2478	pos_2692	Pearson correlation	$[M+NH_4]^+$	[M+H]+	2.795	2.838	374.1438	398.2018	0.903
MSDial	131	131	413	413	pos_2692	pos_2731	Pearson correlation	[M+H]+	[M+H]+	2.838	2.840	398.2018	403.1576	0.929
MSDial	131	131	413	413	pos_2731	pos_2896	pol / adduct	[M+H]+	[M+H]+	2.840	2.857	403.1576	425.1409	1.000
MSDial	133	133	174	174	neg_1216	neg_1235	Pearson correlation	[M-H]-	[M-H]-	3.600	3.598	410.1296	415.1214	0.923
MSDial	133	133	174	174	neg_1216	neg_1308	Pearson correlation	[M-H]-	[M+Na-2H]-	3.600	3.601	410.1296	437.1293	0.955
MSDial	133	133	174	174	neg_1235	neg_1308	pol / adduct	[M-H]-	[M+Na-2H]-	3.598	3.601	415.1214	437.1293	1.000
MSDial	133	133	174	174	neg_1925	neg_1931	Pearson correlation	[M-H]-	[M-H]-	3.589	3.595	817.3634	827.4426	0.917
MSDial	133	133	174	174	neg_1216	neg_394	similar chromatogram in higher mz	[M-H]-	[M-H]-	3.600	3.606	410.1296	213.0149	1.000
MSDial	133	133	174	174	neg_1925	neg_394	similar chromatogram in higher mz	[M-H]-	[M-H]-	3.589	3.606	817.3634	213.0149	1.000

MSDial	133	133	174	174	neg_394	neg_989	similar chromatogram in higher mz	[M-H]$^-$	[M-H]$^-$	3.606	3.600	213.0149	349.1496	1.000
MSDial	133	133	174	174	neg_1235	neg_989	Pearson correlation	[M-H]$^-$	[M-H]$^-$	3.598	3.600	415.1214	349.1496	0.954
MSDial	133	133	174	174	neg_1216	neg_989	Pearson correlation	[M-H]$^-$	[M-H]$^-$	3.600	3.600	410.1296	349.1496	0.932
MSDial	133	133	174	174	neg_1308	neg_989	Pearson correlation	[M+Na-2H]$^-$	[M-H]$^-$	3.601	3.600	437.1293	349.1496	0.904
Adducts/Neutral losses	134	134	541	175	pos_4325	neg_1916	pol / adduct	[M+Na]$^+$	[M-H]$^-$	3.269	3.269	825.2028	801.2080	0.789
MSDial	134	134	175	175	neg_1222	neg_1916	Pearson correlation	[M-H]$^-$	[M-H]$^-$	3.233	3.269	411.1322	801.2080	0.919
Adducts/Neutral losses	134	134	541	175	pos_4300	neg_1916	pol / adduct	[M+H]$^+$	[M-H]$^-$	3.268	3.269	803.2244	801.2080	0.864
MSDial	134	134	175	175	neg_1916	neg_1933	Pearson correlation	[M-H]$^-$	[M+Cl]$^-$	3.269	3.267	801.2080	837.1851	0.920
Adducts/Neutral losses	134	134	541	175	pos_4300	neg_1933	pol / adduct	[M+H]$^+$	[M+Cl]$^-$	3.268	3.267	803.2244	837.1851	0.845
Adducts/Neutral losses	134	134	541	175	pos_4325	neg_1933	pol / adduct	[M+Na]$^+$	[M+Cl]$^-$	3.269	3.267	825.2028	837.1851	0.805
MSDial	134	134	175	175	neg_1916	neg_1933	pol / adduct	[M-H]-	[M+Cl]$^-$	3.269	3.267	801.2080	837.1851	1.000
MSDial	134	134	541	541	pos_2181	pos_4300	Pearson correlation	[M+H]$^+$	[M+H]$^+$	3.272	3.268	347.0756	803.2244	0.927
MSDial	134	134	541	541	pos_2181	pos_4300	found in higher mz's MSMS	[M+H]$^+$	[M+H]$^+$	3.272	3.268	347.0756	803.2244	1.000
MSDial	134	134	541	541	pos_4300	pos_4325	pol / adduct	[M+H]$^+$	[M+Na]$^+$	3.268	3.269	803.2244	825.2028	1.000
MSDial	135	135	176	176	neg_1929	neg_1934	Pearson correlation	[M-H]$^-$	[M-H]$^-$	5.000	4.950	821.3590	837.3532	0.952
Adducts/Neutral losses	138	138	481	262	pos_372	neg_124	pol / adduct	[M+Na]$^+$	[M-H]$^-$	0.740	0.712	157.0127	133.0139	0.886
MSDial	138	138	262	262	neg_124	neg_20	found in higher mz's MSMS	[M-H]$^-$	[M+Cl]$^-$	0.712	0.712	133.0139	71.0137	1.000
MSDial	138	138	262	262	neg_124	neg_20	Pearson correlation	[M-H]$^-$	[M+Cl]$^-$	0.712	0.712	133.0139	71.0137	0.926
MSDial	138	138	262	262	neg_124	neg_814	found in higher mz's MSMS	[M-H]$^-$	[M-H]$^-$	0.712	0.701	133.0139	313.0404	1.000
MSDial	139	139	182	182	neg_125	neg_201	Pearson correlation	[M-H]$^-$	[M-H]$^-$	1.970	1.969	133.0140	161.0451	0.915
MSDial	140	140	183	183	neg_1689	neg_214	found in higher mz's MSMS	[M-H]$^-$	[M-H]$^-$	2.718	2.747	591.0977	163.0398	1.000
MSDial	140	140	183	183	neg_1689	neg_214	Pearson correlation	[M-H]$^-$	[M-H]$^-$	2.718	2.747	591.0977	163.0398	0.905
MSDial	140	140	183	183	neg_214	neg_724	Pearson correlation	[M-H]$^-$	[M-H]$^-$	2.747	2.742	163.0398	295.0453	0.980

MSDial	140	140	183	183	neg_1689	neg_724	Pearson correlation	$[M-H]^-$	$[M-H]^-$	2.718	2.742	591.0977	295.0453	0.924
MSDial	142	142	185	185	neg_1326	neg_235	similar chromatogram in higher mz	$[M-H]^-$	$[M-H]^-$	2.986	2.983	443.1185	172.0975	1.000
MSDial	142	142	185	185	neg_235	neg_495	similar chromatogram in higher mz	$[M-H]^-$	$[M-H]^-$	2.983	2.996	172.0975	239.0920	1.000
MSDial	142	142	185	185	neg_235	neg_994	similar chromatogram in higher mz	$[M-H]^-$	$[M-H]^-$	2.983	2.972	172.0975	351.1288	1.000
MSDial	146	146	190	190	neg_234	neg_263	Pearson correlation	$[M-H]^-$	$[M-H]^-$	1.599	1.589	172.0611	182.0454	0.943
MSDial	151	151	248	248	neg_1517	neg_341	similar chromatogram in higher mz	$[M-H]^-$	$[M-H]^-$	1.465	1.465	513.1450	200.0559	1.000
MSDial	151	151	248	248	neg_1517	neg_725	Pearson correlation	$[M-H]^-$	$[M-H]^-$	1.465	1.471	513.1450	295.1026	0.929
Adducts/Neutral losses	152	152	470	198	pos_3643	neg_1530	pol / adduct	$[M+Na]^+$	$[M-H]^-$	3.980	3.974	543.0720	519.0767	0.889
MSDial	152	152	198	198	neg_1358	neg_1530	Pearson correlation	$[M-H]^-$	$[M-H]^-$	3.971	3.974	453.1790	519.0767	0.905
MSDial	152	152	198	198	neg_1530	neg_1582	Pearson correlation	$[M-H]^-$	$[M+Cl]^-$	3.974	3.980	519.0767	541.0584	0.963
Adducts/Neutral losses	152	152	470	198	pos_3643	neg_1582	pol / adduct	$[M+Na]^+$	$[M+Na-2H]^-$	3.980	3.980	543.0720	541.0584	0.931
Adducts/Neutral losses	152	152	198	198	neg_1684	neg_1582	neutral loss	CH_2O_2	CH_2O_2	3.976	3.980	587.0642	541.0584	0.961
MSDial	152	152	198	198	neg_1530	neg_1619	Pearson correlation	$[M-H]^-$	$[M+Cl]^-$	3.974	3.974	519.0767	555.0537	0.901
MSDial	152	152	198	198	neg_1582	neg_1619	Pearson correlation	$[M+Cl]^-$	$[M+Cl]^-$	3.980	3.974	541.0584	555.0537	0.932
MSDial	152	152	198	198	neg_1530	neg_1619	pol / adduct	$[M-H]^-$	$[M+Cl]^-$	3.974	3.974	519.0767	555.0537	1.000
Adducts/Neutral losses	152	152	470	198	pos_3643	neg_1619	pol / adduct	$[M+Na]^+$	$[M+Cl]^-$	3.980	3.974	543.0720	555.0537	0.967
MSDial	152	152	198	198	neg_1530	neg_1619	found in higher mz's MSMS	$[M-H]^-$	$[M+Cl]^-$	3.974	3.974	519.0767	555.0537	1.000
MSDial	152	152	198	198	neg_1530	neg_1677	found in higher mz's MSMS	$[M-H]^-$	$[M-H]^-$	3.974	3.977	519.0767	582.0722	1.000
MSDial	152	152	198	198	neg_1530	neg_1677	Pearson correlation	$[M-H]^-$	$[M-H]^-$	3.974	3.977	519.0767	582.0722	0.923
MSDial	152	152	198	198	neg_1619	neg_1677	Pearson correlation	$[M+Cl]^-$	$[M-H]^-$	3.974	3.977	555.0537	582.0722	0.960
MSDial	152	152	198	198	neg_1582	neg_1677	Pearson correlation	$[M+Cl]^-$	$[M-H]^-$	3.980	3.977	541.0584	582.0722	0.947
MSDial	152	152	198	198	neg_1530	neg_1684	Pearson correlation	$[M-H]^-$	$[M-H]^-$	3.974	3.976	519.0767	587.0642	0.924
MSDial	152	152	198	198	neg_1619	neg_1684	Pearson correlation	$[M+Cl]^-$	$[M-H]^-$	3.974	3.976	555.0537	587.0642	0.915

Adducts/Neutral losses	152	152	198	198	neg_1756	neg_1684	neutral loss	CO_2	CO_2	3.951	3.976	631.0549	587.0642	0.841
MSDial	152	152	198	198	neg_1677	neg_1684	Pearson correlation	$[M-H]^-$	$[M-H]^-$	3.977	3.976	582.0722	587.0642	0.919
MSDial	152	152	198	198	neg_1582	neg_1684	found in higher mz's MSMS	$[M+Cl]^-$	$[M-H]^-$	3.980	3.976	541.0584	587.0642	1.000
MSDial	152	152	198	198	neg_1582	neg_1684	Pearson correlation	$[M+Cl]^-$	$[M-H]^-$	3.980	3.976	541.0584	587.0642	0.961
MSDial	152	152	198	198	neg_1684	neg_1714	pol / adduct	$[M-H]^-$	$[M+Na-2H]^-$	3.976	3.969	587.0642	609.0720	1.000
MSDial	152	152	198	198	neg_1530	neg_1714	found in higher mz's MSMS	$[M-H]^-$	$[M+Na-2H]^-$	3.974	3.969	519.0767	609.0720	1.000
MSDial	152	152	198	198	neg_1619	neg_1714	Pearson correlation	$[M+Cl]^-$	$[M+Na-2H]^-$	3.974	3.969	555.0537	609.0720	0.906
MSDial	152	152	198	198	neg_1695	neg_1714	Pearson correlation	$[M-H]^-$	$[M+Na-2H]^-$	4.016	3.969	593.0775	609.0720	0.945
MSDial	152	152	198	198	neg_1582	neg_1756	found in higher mz's MSMS	$[M+Cl]^-$	$[M-H]^-$	3.980	3.951	541.0584	631.0549	1.000
MSDial	152	152	198	198	neg_1530	neg_1942	found in higher mz's MSMS	$[M-H]^-$	$[M-H]^-$	3.974	3.962	519.0767	857.1403	1.000
MSDial	152	152	198	198	neg_1695	neg_345	similar chromatogram in higher mz	$[M-H]^-$	$[M-H]^-$	4.016	4.009	593.0775	201.1128	1.000
MSDial	152	152	198	198	neg_1714	neg_951	found in higher mz's MSMS	$[M+Na-2H]^-$	$[M-H]^-$	3.969	3.972	609.0720	343.0427	1.000
MSDial	152	152	198	198	neg_1582	neg_951	Pearson correlation	$[M+Cl]^-$	$[M-H]^-$	3.980	3.972	541.0584	343.0427	0.925
MSDial	152	152	198	198	neg_1684	neg_951	Pearson correlation	$[M-H]^-$	$[M-H]^-$	3.976	3.972	587.0642	343.0427	0.904
MSDial	152	152	198	198	neg_1677	neg_951	found in higher mz's MSMS	$[M-H]^-$	$[M-H]^-$	3.977	3.972	582.0722	343.0427	1.000
MSDial	152	152	198	198	neg_1619	neg_951	found in higher mz's MSMS	$[M+Cl]^-$	$[M-H]^-$	3.974	3.972	555.0537	343.0427	1.000
MSDial	152	152	198	198	neg_1530	neg_951	Pearson correlation	$[M-H]^-$	$[M-H]^-$	3.974	3.972	519.0767	343.0427	0.965
MSDial	152	152	198	198	neg_1942	neg_951	found in higher mz's MSMS	$[M-H]^-$	$[M-H]^-$	3.962	3.972	857.1403	343.0427	1.000
MSDial	152	152	198	198	neg_1530	neg_951	found in higher mz's MSMS	$[M-H]^-$	$[M-H]^-$	3.974	3.972	519.0767	343.0427	1.000
MSDial	152	152	198	198	neg_1756	neg_951	found in higher mz's MSMS	$[M-H]^-$	$[M-H]^-$	3.951	3.972	631.0549	343.0427	1.000
MSDial	157	157	204	204	neg_1359	neg_408	similar chromatogram in higher mz	$[M-H]^-$	$[M-H]^-$	3.479	3.491	453.1792	217.1076	1.000
MSDial	163	163	213	213	neg_1407	neg_482	Pearson correlation	$[M+Cl]^-$	$[M+Cl]^-$	1.985	2.005	473.1501	237.0432	0.932
MSDial	172	172	227	227	neg_248	neg_590	Pearson correlation	$[M-H]^-$	$[M-H]^-$	0.647	0.657	175.0468	265.0418	0.974
MSDial	177	177	234	234	neg_1018	neg_649	Pearson correlation	$[M-H]^-$	$[M-H]^-$	5.006	5.028	357.1086	277.1438	0.969
MSDial	180	180	237	237	neg_1700	neg_677	similar chromatogram in higher mz	$[M-H]^-$	$[M-H]^-$	5.216	5.212	595.2876	283.1908	1.000

MSDial	180	180	237	237	neg_1700	neg_727	similar chromatogram in higher mz	$[M-H]^-$	$[M-H]^-$	5.216	5.220	595.2876	295.2272	1.000
MSDial	183	183	240	240	neg_1207	neg_685	similar chromatogram in higher mz	$[M-H]^-$	$[M-H]^-$	2.014	2.018	405.0520	285.1086	1.000
MSDial	189	189	249	249	neg_671	neg_729	pol / adduct	$[M+Na-2H]^-$	$[M+Cl]^-$	0.653	0.658	282.0822	296.0614	1.000
MSDial	194	194	257	257	neg_183	neg_782	similar chromatogram in higher mz	$[M-H]^-$	$[M-H]^-$	2.902	2.910	157.0502	307.1214	1.000
MSDial	196	196	259	259	neg_1874	neg_800	Pearson correlation	$[M-H]^-$	$[M-H]^-$	2.352	2.389	727.1925	310.1402	0.910
Adducts/Neutral losses	196	196	355	259	pos_2048	neg_800	pol / adduct	$[M+Na]^+$	$[M-H]^-$	2.393	2.389	334.1366	310.1402	0.881
Adducts/Neutral losses	197	197	251	251	neg_804	neg_731	neutral loss	CH_2	CH_2	2.224	2.204	311.0798	297.0608	0.093
MSDial	197	197	251	251	neg_279	neg_731	similar chromatogram in higher mz	$[M-H]^-$	$[M+Na-2H]^-$	2.229	2.204	187.0607	297.0608	1.000
MSDial	197	197	251	251	neg_731	neg_804	pol / adduct	$[M+Na-2H]^-$	$[M+Cl]^-$	2.204	2.224	297.0608	311.0798	1.000
Adducts/Neutral losses	200	200	442	254	pos_1312	neg_598	pol / adduct	$[M+H]^+$	[M-H]-	1.560	1.558	268.1030	266.0889	0.968
Adducts/Neutral losses	200	200	254	254	neg_813	neg_598	neutral loss	CH_2O_2	CH_2O_2	1.559	1.558	312.0943	266.0889	0.981
Adducts/Neutral losses	200	200	442	254	pos_1556	neg_598	pol / adduct	$[M+Na]^+$	$[M-H]^-$	1.561	1.558	290.0856	266.0889	0.767
Adducts/Neutral losses	200	200	254	254	neg_813	neg_672	neutral loss	CH_2O	CH_2O	1.559	1.549	312.0943	282.0838	0.927
Adducts/Neutral losses	200	200	324	254	pos_1484	neg_672	pol / adduct	$[M+H]^+$	$[M-H]^-$	1.545	1.549	284.0986	282.0838	0.986
MSDial	200	200	254	254	neg_598	neg_672	Pearson correlation	$[M-H]^-$	$[M-H]^-$	1.558	1.549	266.0889	282.0838	0.902
Adducts/Neutral losses	200	200	254	254	neg_887	neg_672	neutral loss	CH_2O_2	CH_2O_2	1.550	1.549	328.0891	282.0838	0.994
MSDial	200	200	254	254	neg_598	neg_757	Pearson correlation	$[M-H]^-$	$[M+Cl]^-$	1.558	1.559	266.0889	302.0655	0.975
Adducts/Neutral losses	200	200	442	254	pos_1556	neg_757	pol / adduct	$[M+Na]^+$	$[M+Cl]^-$	1.561	1.559	290.0856	302.0655	0.798
MSDial	200	200	254	254	neg_598	neg_757	pol / adduct	$[M-H]^-$	$[M+Cl]^-$	1.558	1.559	266.0889	302.0655	1.000
Adducts/Neutral losses	200	200	442	254	pos_1312	neg_757	pol / adduct	[M+H]+	$[M+Cl]^-$	1.560	1.559	268.1030	302.0655	0.982

MSDial	200	200	254	254	neg_672	neg_813	Pearson correlation	$[M-H]^-$	$[M-H]^-$	1.549	1.559	282.0838	312.0943	0.927
MSDial	200	200	254	254	neg_598	neg_813	found in higher mz's MSMS	$[M-H]^-$	$[M-H]^-$	1.558	1.559	266.0889	312.0943	1.000
Adducts/Neutral losses	200	200	442	254	pos_1556	neg_813	pol / adduct	$[M+Na]^+$	$[M+FA-H]^-$	1.561	1.559	290.0856	312.0943	0.772
MSDial	200	200	254	254	neg_757	neg_813	Pearson correlation	$[M+Cl]^-$	$[M-H]^-$	1.559	1.559	302.0655	312.0943	0.985
MSDial	200	200	254	254	neg_598	neg_813	Pearson correlation	$[M-H]^-$	$[M-H]^-$	1.558	1.559	266.0889	312.0943	0.981
Adducts/Neutral losses	200	200	442	254	pos_1312	neg_813	pol / adduct	$[M+H]^+$	$[M+FA-H]^-$	1.560	1.559	268.1030	312.0943	0.981
MSDial	200	200	254	254	neg_813	neg_887	Pearson correlation	$[M-H]^-$	$[M-H]^-$	1.559	1.550	312.0943	328.0891	0.923
Adducts/Neutral losses	200	200	324	254	pos_1484	neg_887	pol / adduct	$[M+H]^+$	$[M+FA-H]^-$	1.545	1.550	284.0986	328.0891	0.970
MSDial	200	200	254	254	neg_672	neg_887	Pearson correlation	$[M-H]^-$	$[M-H]^-$	1.549	1.550	282.0838	328.0891	0.994
MSDial	200	200	442	442	pos_1312	pos_1556	pol / adduct	$[M+H]^+$	$[M+Na]^+$	1.560	1.561	268.1030	290.0856	1.000
MSDial	200	200	442	442	pos_1556	pos_2240	Pearson correlation	$[M+Na]^+$	$[M+H]^+$	1.561	1.565	290.0856	352.0551	0.925
MSDial	200	200	442	442	pos_1312	pos_3309	found in higher mz's MSMS	$[M+H]^+$	$[M+Na]^+$	1.560	1.571	268.1030	481.1847	1.000
Adducts/Neutral losses	205	205	268	268	neg_827	neg_620	neutral loss	C_2H_4O	C_2H_4O	3.500	3.548	315.1803	271.1546	0.965
Adducts/Neutral losses	205	205	333	268	pos_1561	neg_620	pol / adduct	$[M+NH_4]^+$	$[M-H]^-$	3.551	3.548	290.1957	271.1546	0.980
MSDial	205	205	268	268	neg_620	neg_827	Pearson correlation	$[M-H]^-$	$[M-H]^-$	3.548	3.500	271.1546	315.1803	0.965
MSDial	206	206	269	269	neg_530	neg_835	Pearson correlation	$[M-H]^-$	$[M-H]^-$	3.712	3.737	247.1545	317.1962	0.942
MSDial	214	214	280	280	neg_1133	neg_1153	Pearson correlation	$[M-H]^-$	$[M-H]^-$	4.370	4.371	385.1755	390.2126	0.929
MSDial	214	214	280	280	neg_1133	neg_885	Pearson correlation	$[M-H]^-$	$[M-H]^-$	4.370	4.370	385.1755	327.2170	0.926
MSDial	214	214	280	280	neg_1153	neg_885	similar chromatogram in higher mz	$[M-H]^-$	$[M-H]^-$	4.371	4.370	390.2126	327.2170	1.000
MSDial	214	214	280	280	neg_1153	neg_885	Pearson correlation	$[M-H]^-$	$[M-H]^-$	4.371	4.370	390.2126	327.2170	0.942
Adducts/Neutral losses	214	214	335	280	pos_1793	neg_885	pol / adduct	$[M+H-H_2O]^+$	$[M-H]^-$	4.371	4.370	311.2214	327.2170	0.975
MSDial	214	214	280	280	neg_1698	neg_885	similar chromatogram in higher mz	$[M-H]^-$	$[M-H]^-$	4.372	4.370	593.3530	327.2170	1.000

MSDial	214	214	280	280	neg_1133	neg_885	similar chromatogram in higher mz	$[M-H]^-$	$[M-H]^-$	4.370	4.370	385.1755	327.2170	1.000
MSDial	217	217	285	285	neg_1090	neg_1182	pol / adduct	$[M-H]^-$	$[M+Na-2H]^-$	2.336	2.333	375.0560	397.0379	1.000
MSDial	217	217	285	285	neg_1090	neg_1182	Pearson correlation	$[M-H]^-$	$[M+Na-2H]^-$	2.336	2.333	375.0560	397.0379	0.936
Adducts/Neutral losses	217	217	581	285	pos_1053	neg_356	pol / adduct	[M+K]+	$[M-H]^-$	2.340	2.345	243.0518	203.0821	0.712
Adducts/Neutral losses	217	217	581	285	pos_732	neg_356	pol / adduct	[M+H]+	$[M-H]^-$	2.347	2.345	205.0968	203.0821	0.950
Adducts/Neutral losses	217	217	581	285	pos_905	neg_356	pol / adduct	$[M+Na]^+$	$[M-H]^-$	2.349	2.345	227.0783	203.0821	0.559
MSDial	217	217	285	285	neg_1608	neg_356	similar chromatogram in higher mz	$[M-H]^-$	$[M-H]^-$	2.346	2.345	552.1348	203.0821	1.000
Adducts/Neutral losses	217	217	285	285	neg_597	neg_423	neutral loss	CHO_2	CHO_2	2.349	2.327	266.0664	221.0675	0.622
MSDial	217	217	285	285	neg_356	neg_575	similar chromatogram in higher mz	$[M-H]^-$	$[M+Na-2H]^-$	2.345	2.345	203.0821	261.0406	1.000
MSDial	217	217	285	285	neg_423	neg_575	pol / adduct	$[M-H_2O-H]^-$	$[M+Na-2H]^-$	2.327	2.345	221.0675	261.0406	1.000
MSDial	217	217	285	285	neg_356	neg_619	found in higher mz's MSMS	$[M-H]^-$	$[M+Na-2H]^-$	2.345	2.346	203.0821	271.0695	1.000
MSDial	217	217	285	285	neg_1608	neg_684	found in higher mz's MSMS	$[M-H]^-$	$[M-H]^-$	2.346	2.337	552.1348	285.0610	1.000
MSDial	217	217	285	285	neg_1182	neg_684	found in higher mz's MSMS	$[M+Na-2H]^-$	$[M-H]^-$	2.333	2.337	397.0379	285.0610	1.000
MSDial	217	217	285	285	neg_1090	neg_684	found in higher mz's MSMS	$[M-H]^-$	$[M-H]^-$	2.336	2.337	375.0560	285.0610	1.000
MSDial	217	217	285	285	neg_356	neg_910	found in higher mz's MSMS	$[M-H]^-$	$[M+Na-2H]^-$	2.345	2.348	203.0821	333.0399	1.000
Adducts/Neutral losses	217	217	581	285	pos_1541	neg_910	pol / adduct	[M+H]+	$[M+FA-H]^-$	2.351	2.348	289.0494	333.0399	0.840
MSDial	217	217	285	285	neg_619	neg_910	Pearson correlation	$[M+Na-2H]^-$	[M+Na-2H]-	2.346	2.348	271.0695	333.0399	0.934
MSDial	217	217	285	285	neg_684	neg_984	found in higher mz's MSMS	$[M-H]^-$	$[M-H]^-$	2.337	2.334	285.0610	348.0563	1.000
MSDial	217	217	285	285	neg_356	neg_984	similar chromatogram in higher mz	$[M-H]^-$	$[M-H]^-$	2.345	2.334	203.0821	348.0563	1.000
MSDial	217	217	581	581	pos_1053	pos_1541	found in higher mz's MSMS	[M+K]+	$[M+H]^+$	2.340	2.351	243.0518	289.0494	1.000
MSDial	217	217	581	581	pos_389	pos_732	Pearson correlation	[M+H]+	$[M+H]^+$	2.346	2.347	159.0911	205.0968	0.976
MSDial	217	217	581	581	pos_1053	pos_732	pol / adduct	[M+K]+	$[M+H]^+$	2.340	2.347	243.0518	205.0968	1.000
MSDial	217	217	581	581	pos_732	pos_905	pol / adduct	$[M+H]^+$	$[M+Na]^+$	2.347	2.349	205.0968	227.0783	1.000

MSDial	218	218	286	286	neg_1084	neg_163	similar chromatogram in higher mz	[M-H]$^-$	[M-H]$^-$	1.764	1.758	373.0627	147.0659	1.000
MSDial	218	218	286	286	neg_1189	neg_163	similar chromatogram in higher mz	[M-H]$^-$	[M-H]$^-$	1.758	1.758	399.1134	147.0659	1.000
Adducts/Neutral losses	218	218	326	286	pos_1496	neg_674	pol / adduct	[M+H]$^+$	[M-H]$^-$	1.762	1.764	285.0822	283.0678	0.980
MSDial	218	218	286	286	neg_1084	neg_674	found in higher mz's MSMS	[M-H]$^-$	[M-H]$^-$	1.764	1.764	373.0627	283.0678	1.000
MSDial	218	218	286	286	neg_1084	neg_674	Pearson correlation	[M-H]$^-$	[M-H]$^-$	1.764	1.764	373.0627	283.0678	0.944
MSDial	218	218	286	286	neg_163	neg_674	similar chromatogram in higher mz	[M-H]$^-$	[M-H]$^-$	1.758	1.764	147.0659	283.0678	1.000
MSDial	218	218	286	286	neg_1189	neg_911	Pearson correlation	[M-H]$^-$	[M-H]$^-$	1.758	1.725	399.1134	333.0454	0.941
MSDial	220	220	289	289	neg_1434	neg_935	similar chromatogram in higher mz	[M-H]$^-$	[M+K-2H]$^-$	5.278	5.276	481.2560	339.1990	1.000
Adducts/Neutral losses	225	225	310	281	pos_1071	neg_658	pol / adduct	[M+H]$^+$	[M+Cl]-	1.139	1.166	245.0764	279.0383	0.974
MSDial	225	225	281	281	neg_658	neg_886	Pearson correlation	[M-H]$^-$	[M-H]$^-$	1.166	1.134	279.0383	328.0443	0.935
MSDial	225	225	281	281	neg_886	neg_964	Pearson correlation	[M-H]$^-$	[M-H]$^-$	1.134	1.157	328.0443	344.0393	0.989
MSDial	225	225	281	281	neg_658	neg_964	Pearson correlation	[M-H]$^-$	[M-H]$^-$	1.166	1.157	279.0383	344.0393	0.920
MSDial	226	226	295	295	neg_1198	neg_298	Pearson correlation	[M-H]$^-$	[M-H]$^-$	2.434	2.423	403.0906	190.0539	0.901
MSDial	226	226	295	295	neg_294	neg_723	Pearson correlation	[M-H]$^-$	[M-H]$^-$	2.394	2.427	189.0764	295.0451	0.905
MSDial	226	226	295	295	neg_1198	neg_723	Pearson correlation	[M-H]$^-$	[M-H]$^-$	2.434	2.427	403.0906	295.0451	0.907
MSDial	226	226	295	295	neg_298	neg_723	similar chromatogram in higher mz	[M-H]$^-$	[M-H]$^-$	2.423	2.427	190.0539	295.0451	1.000
MSDial	226	226	295	295	neg_1482	neg_723	Pearson correlation	[M-H]$^-$	[M-H]$^-$	2.421	2.427	501.1240	295.0451	0.906
MSDial	226	226	295	295	neg_298	neg_965	similar chromatogram in higher mz	[M-H]$^-$	[M-H]$^-$	2.423	2.423	190.0539	344.0979	1.000
MSDial	227	227	298	298	neg_202	neg_975	similar chromatogram in higher mz	[M-H]$^-$	[M-H]$^-$	1.401	1.423	161.0451	346.0548	1.000
Adducts/Neutral losses	227	227	369	298	pos_2193	neg_975	pol / adduct	[M+H]$^+$	[M-H]$^-$	1.421	1.423	348.0701	346.0548	0.992
Adducts/Neutral losses	228	228	300	222	pos_1008	neg_541	pol / adduct	[M+Na]+	[M+Cl]$^-$	3.891	3.897	238.1403	250.1212	0.524
Adducts/Neutral losses	241	241	590	218	pos_1291	neg_506	pol / adduct	[M+Na]+	[M-H]$^-$	4.422	4.421	266.1720	242.1758	0.900

Adducts/Neutral losses	241	241	590	218	pos_901	neg_506	pol / adduct	$[M+H-H_2O]^+$	$[M-H]^-$	4.423	4.421	226.1796	242.1758	0.941
MSDial	241	241	590	590	pos_1291	pos_901	Pearson correlation	$[M+NH_4]^+$	$[M+H-H_2O]^+$	4.422	4.423	266.1720	226.1796	0.920
MSDial	242	242	229	229	neg_389	neg_395	found in higher mz's MSMS	$[M-H]^-$	[M-H2O-H]$^-$	1.950	1.943	212.0557	213.0876	1.000
Adducts/Neutral losses	242	242	314	229	pos_1300	neg_591	pol / adduct	$[M+H]^+$	$[M-H]^-$	1.916	1.917	267.0973	265.0823	0.905
MSDial	242	242	229	229	neg_395	neg_591	Pearson correlation	$[M-H_2O-H]^-$	$[M-H]^-$	1.943	1.917	213.0876	265.0823	0.900
MSDial	242	242	229	229	neg_389	neg_605	similar chromatogram in higher mz	$[M-H]^-$	$[M+Cl]^-$	1.950	1.949	212.0557	267.1080	1.000
MSDial	242	242	229	229	neg_395	neg_605	pol / adduct	$[M-H_2O-H]^-$	$[M+Cl]^-$	1.943	1.949	213.0876	267.1080	1.000
Adducts/Neutral losses	247	247	317	231	pos_1361	neg_617	pol / adduct	$[M+H]^+$	$[M-H]^-$	4.819	4.806	272.2218	270.2067	0.843
Adducts/Neutral losses	252	252	322	214	pos_1456	neg_493	pol / adduct	$[M+Na]^+$	$[M-H_2O-H]^-$	4.623	4.643	281.0799	239.0669	0.494
Adducts/Neutral losses	255	255	327	230	pos_1512	neg_606	pol / adduct	$[M+NH_4]^+$	$[M-H]^-$	3.914	3.910	286.1638	267.1232	0.920
MSDial	272	272	345	345	pos_1631	pos_1861	Pearson correlation	$[M+NH_4]^+$	$[M+K]^+$	5.607	5.599	296.2577	317.1785	0.936
MSDial	272	272	345	345	pos_1631	pos_1861	pol / adduct	$[M+NH_4]^+$	$[M+K]^+$	5.607	5.599	296.2577	317.1785	1.000
Adducts/Neutral losses	273	273	583	583	pos_1864	pos_1660	neutral loss	H_2O	H_2O	0.552	0.553	318.0845	300.0740	0.965
MSDial	273	273	583	583	pos_1660	pos_1864	pol / adduct	$[M+H-H_2O]^+$	$[M+H]^+$	0.553	0.552	300.0740	318.0845	1.000
MSDial	273	273	583	583	pos_1660	pos_1864	Pearson correlation	$[M+H-H_2O]^+$	$[M+H]^+$	0.553	0.552	300.0740	318.0845	0.965
MSDial	273	273	583	583	pos_1660	pos_330	Pearson correlation	$[M+H-H_2O]^+$	$[M+H]^+$	0.553	0.553	300.0740	151.0085	0.964
MSDial	273	273	583	583	pos_1864	pos_330	Pearson correlation	$[M+H]^+$	$[M+H]^+$	0.552	0.553	318.0845	151.0085	0.969
Adducts/Neutral losses	278	278	350	216	pos_824	neg_486	pol / adduct	$[M+H]^+$	$[M+Na-2H]^-$	4.767	4.817	217.1795	237.1492	0.436
Adducts/Neutral losses	278	278	350	216	pos_687	neg_486	pol / adduct	$[M+H-H_2O]^+$	$[M+Na-2H]^-$	4.770	4.817	199.1686	237.1492	0.358
MSDial	278	278	216	216	neg_486	neg_503	similar chromatogram in higher mz	$[M-H]^-$	$[M-H]^-$	4.817	4.812	237.1492	241.1440	1.000
MSDial	278	278	350	350	pos_1016	pos_114	Pearson correlation	$[M+Na]^+$	$[M+H-H_2O]^+$	4.766	4.766	239.1614	111.1165	0.945

Adducts/Neutral losses	278	278	350	350	pos_198	pos_114	neutral loss	H_2O	H_2O	4.762	4.766	129.1271	111.1165	0.964
MSDial	278	278	350	350	pos_114	pos_198	Pearson correlation	$[M+H-H_2O]^+$	$[M+H]^+$	4.766	4.762	111.1165	129.1271	0.964
MSDial	278	278	350	350	pos_1016	pos_198	Pearson correlation	$[M+Na]^+$	$[M+H]^+$	4.766	4.762	239.1614	129.1271	0.972
MSDial	278	278	350	350	pos_114	pos_198	found in higher mz's MSMS	$[M+H-H_2O]^+$	$[M+H]^+$	4.766	4.762	111.1165	129.1271	1.000
MSDial	278	278	350	350	pos_114	pos_198	pol / adduct	$[M+H-H_2O]^+$	$[M+H]^+$	4.766	4.762	111.1165	129.1271	1.000
MSDial	278	278	350	350	pos_1016	pos_687	Pearson correlation	$[M+Na]^+$	$[M+H-H_2O]^+$	4.766	4.770	239.1614	199.1686	0.958
MSDial	278	278	350	350	pos_114	pos_687	found in higher mz's MSMS	$[M+H-H_2O]^+$	$[M+H-H_2O]^+$	4.766	4.770	111.1165	199.1686	1.000
Adducts/Neutral losses	278	278	350	350	pos_824	pos_687	neutral loss	H_2O	H_2O	4.767	4.770	217.1795	199.1686	0.974
MSDial	278	278	350	350	pos_1016	pos_687	pol / adduct	$[M+Na]^+$	$[M+H-H_2O]^+$	4.766	4.770	239.1614	199.1686	1.000
MSDial	278	278	350	350	pos_114	pos_687	Pearson correlation	$[M+H-H_2O]^+$	$[M+H-H_2O]^+$	4.766	4.770	111.1165	199.1686	0.963
MSDial	278	278	350	350	pos_198	pos_687	Pearson correlation	$[M+H]^+$	$[M+H-H_2O]^+$	4.762	4.770	129.1271	199.1686	0.970
MSDial	278	278	350	350	pos_687	pos_824	Pearson correlation	$[M+H-H_2O]^+$	$[M+H]^+$	4.770	4.767	199.1686	217.1795	0.974
MSDial	278	278	350	350	pos_1016	pos_824	Pearson correlation	$[M+Na]^+$	$[M+H]^+$	4.766	4.767	239.1614	217.1795	0.967
MSDial	278	278	350	350	pos_198	pos_824	Pearson correlation	$[M+H]^+$	$[M+H]^+$	4.762	4.767	129.1271	217.1795	0.978
MSDial	278	278	350	350	pos_687	pos_824	pol / adduct	$[M+H-H_2O]^+$	$[M+H]^+$	4.770	4.767	199.1686	217.1795	1.000
MSDial	278	278	350	350	pos_198	pos_824	found in higher mz's MSMS	$[M+H]^+$	$[M+H]^+$	4.762	4.767	129.1271	217.1795	1.000
MSDial	278	278	350	350	pos_114	pos_824	found in higher mz's MSMS	$[M+H-H_2O]^+$	$[M+H]^+$	4.766	4.767	111.1165	217.1795	1.000
MSDial	278	278	350	350	pos_114	pos_824	Pearson correlation	$[M+H-H_2O]^+$	$[M+H]^+$	4.766	4.767	111.1165	217.1795	0.950
Adducts/Neutral losses	283	283	356	2	pos_2058	neg_1016	pol / adduct	$[M+Na]^+$	$[M+FA-H]^-$	0.696	0.671	335.0628	357.0667	0.226
Adducts/Neutral losses	284	284	358	14	pos_2115	neg_1088	pol / adduct	$[M+H]^+$	$[M+Cl]^-$	3.043	3.039	340.2588	374.2209	0.993
Adducts/Neutral losses	287	287	361	290	pos_2136	neg_938	pol / adduct	$[M+H]^+$	$[M-H]^-$	3.840	3.834	342.2272	340.2118	0.976
Adducts/Neutral losses	288	288	362	362	pos_2137	pos_1943	neutral loss	H_3N	H_3N	5.288	5.286	342.2631	325.2364	0.996

MSDial	288	288	362	362	pos_1943	pos_2137	pol / adduct	$[M+H]^+$	$[M+NH_4]^+$	5.286	5.288	325.2364	342.2631	1.000
MSDial	288	288	362	362	pos_1943	pos_2137	Pearson correlation	$[M+H]^+$	$[M+NH_4]^+$	5.286	5.288	325.2364	342.2631	0.996
MSDial	288	288	362	362	pos_1943	pos_2185	pol / adduct	$[M+H]^+$	$[M+Na]^+$	5.286	5.288	325.2364	347.2184	1.000
MSDial	288	288	362	362	pos_1943	pos_2185	Pearson correlation	$[M+H]^+$	$[M+Na]^+$	5.286	5.288	325.2364	347.2184	0.985
MSDial	288	288	362	362	pos_2137	pos_2185	Pearson correlation	$[M+NH_4]^+$	$[M+Na]^+$	5.288	5.288	342.2631	347.2184	0.988
Adducts/Neutral losses	290	290	364	234	pos_2151	neg_1144	pol / adduct	$[M+H]^+$	$[M+FA-H]^-$	5.017	5.023	343.2941	387.2855	0.984
MSDial	291	291	366	366	pos_1441	pos_2174	Pearson correlation	$[M+H]^+$	$[M+NH_4]^+$	2.492	2.510	280.0803	346.1487	0.924
MSDial	292	292	367	367	pos_1165	pos_1396	pol / adduct	$[M+H]^+$	$[M+Na]^+$	5.521	5.521	254.2476	276.2294	1.000
MSDial	292	292	367	367	pos_1578	pos_2077	Pearson correlation	$[M+H]^+$	$[M+NH_4]^+$	5.540	5.537	292.2838	336.3107	0.905
MSDial	292	292	367	367	pos_1165	pos_2077	Pearson correlation	$[M+H]^+$	$[M+NH_4]^+$	5.521	5.537	254.2476	336.3107	0.912
MSDial	292	292	367	367	pos_1396	pos_2177	Pearson correlation	$[M+Na]^+$	$[M+H]^+$	5.521	5.557	276.2294	346.3303	0.904
MSDial	292	292	367	367	pos_1165	pos_3826	Pearson correlation	$[M+H]^+$	$[M+H]^+$	5.521	5.479	254.2476	600.4679	0.938
MSDial	292	292	367	367	pos_2077	pos_3873	Pearson correlation	$[M+NH_4]^+$	$[M+H]^+$	5.537	5.564	336.3107	614.4827	0.938
MSDial	292	292	367	367	pos_1165	pos_3873	Pearson correlation	$[M+H]^+$	$[M+H]^+$	5.521	5.564	254.2476	614.4827	0.947
MSDial	292	292	367	367	pos_1578	pos_3873	Pearson correlation	$[M+H]^+$	$[M+H]^+$	5.540	5.564	292.2838	614.4827	0.909
MSDial	292	292	367	367	pos_3873	pos_4030	Pearson correlation	$[M+H]^+$	$[M+NH_4]^+$	5.564	5.563	614.4827	658.5077	0.983
MSDial	292	292	367	367	pos_1578	pos_4030	Pearson correlation	$[M+H]^+$	$[M+NH_4]^+$	5.540	5.563	292.2838	658.5077	0.905
MSDial	292	292	367	367	pos_1165	pos_4030	Pearson correlation	$[M+H]^+$	$[M+NH_4]^+$	5.521	5.563	254.2476	658.5077	0.938
MSDial	292	292	367	367	pos_2077	pos_4030	Pearson correlation	$[M+NH_4]^+$	$[M+NH_4]^+$	5.537	5.563	336.3107	658.5077	0.915
Adducts/Neutral losses	293	293	76	76	neg_1557	neg_1490	neutral loss	CH_2O	CH_2O	4.057	4.030	533.0925	503.0824	0.959
Adducts/Neutral losses	293	293	458	76	pos_3557	neg_1490	pol / adduct	$[M+Na]^+$	$[M-H]^-$	4.015	4.030	527.0791	503.0824	0.878
MSDial	293	293	76	76	neg_1490	neg_1557	Pearson correlation	$[M-H]^-$	$[M-H]^-$	4.030	4.057	503.0824	533.0925	0.959
MSDial	293	293	76	76	neg_1557	neg_1655	pol / adduct	$[M-H]^-$	$[M+K-2H]^-$	4.057	4.015	533.0925	571.0665	1.000

Adducts/Neutral losses	293	293	458	296	pos_2182	neg_968	pol / adduct	$[M+H]^+$	$[M-H]^-$	4.044	4.048	347.0758	345.0608	0.977
MSDial	293	293	458	458	pos_2182	pos_3557	found in higher mz's MSMS	$[M+H]^+$	$[M+Na]^+$	4.044	4.015	347.0758	527.0791	1.000
Adducts/Neutral losses	294	294	368	73	pos_3666	neg_1552	pol / adduct	$[M+NH_4]^+$	$[M-H]^-$	2.549	2.547	550.1747	531.1343	0.936
Adducts/Neutral losses	294	294	368	276	pos_754	neg_293	pol / adduct	$[M+NH_4]^+$	$[M-H]^-$	2.571	2.576	208.1176	189.0764	0.909
Adducts/Neutral losses	294	294	368	276	pos_2132	neg_865	pol / adduct	$[M+NH_4]^+$	$[M-H]^-$	2.555	2.557	342.1747	323.1340	0.958
MSDial	294	294	276	276	neg_293	neg_865	Pearson correlation	$[M-H]^-$	$[M-H_2O-H]^-$	2.576	2.557	189.0764	323.1340	0.905
MSDial	294	294	276	276	neg_596	neg_865	similar chromatogram in higher mz	$[M-H]^-$	$[M-H_2O-H]^-$	2.572	2.557	266.0299	323.1340	1.000
Adducts/Neutral losses	294	294	368	276	pos_2183	neg_865	pol / adduct	[M+Na]+	$[M-H]^-$	2.554	2.557	347.1311	323.1340	0.934
MSDial	294	294	368	368	pos_2132	pos_2183	Pearson correlation	$[M+CH_3OH+H]^+$	$[M+H]^+$	2.555	2.554	342.1747	347.1311	0.964
MSDial	294	294	368	368	pos_2132	pos_3666	Pearson correlation	$[M+CH_3OH+H]^+$	$[M+NH_4]^+$	2.555	2.549	342.1747	550.1747	0.904
MSDial	294	294	368	368	pos_2183	pos_3666	Pearson correlation	$[M+H]^+$	$[M+NH_4]^+$	2.554	2.549	347.1311	550.1747	0.911
MSDial	294	294	368	368	pos_2183	pos_754	Pearson correlation	$[M+H]^+$	$[M+NH_4]^+$	2.554	2.571	347.1311	208.1176	0.912
MSDial	294	294	368	368	pos_2132	pos_754	Pearson correlation	$[M+CH_3OH+H]^+$	$[M+NH_4]^+$	2.555	2.571	342.1747	208.1176	0.949
MSDial	294	294	368	368	pos_3666	pos_754	Pearson correlation	$[M+NH_4]^+$	$[M+NH_4]^+$	2.549	2.571	550.1747	208.1176	0.914
MSDial	295	295	90	90	neg_1067	neg_126	Pearson correlation	$[M+Na-2H]^-$	$[M-H]^-$	3.125	3.128	369.0452	133.0140	0.911
MSDial	295	295	90	90	neg_1067	neg_126	found in higher mz's MSMS	$[M+Na-2H]^-$	$[M-H]^-$	3.125	3.128	369.0452	133.0140	1.000
MSDial	295	295	90	90	neg_126	neg_1549	found in higher mz's MSMS	$[M-H]^-$	$[M-H]^-$	3.128	3.087	133.0140	530.1289	1.000
MSDial	295	295	90	90	neg_126	neg_1672	found in higher mz's MSMS	$[M-H]^-$	$[M+Na-2H]^-$	3.128	3.127	133.0140	581.0903	1.000
Adducts/Neutral losses	295	295	370	90	pos_3760	neg_1672	pol / adduct	$[M+NH_4]^+$	$[M+Na-2H]^-$	3.124	3.127	578.1494	581.0903	0.923
MSDial	295	295	90	90	neg_126	neg_1672	similar chromatogram in higher mz	$[M-H]^-$	$[M+Na-2H]^-$	3.128	3.127	133.0140	581.0903	1.000
MSDial	295	295	90	90	neg_1067	neg_213	found in higher mz's MSMS	$[M+Na-2H]^-$	$[M-H]^-$	3.125	3.121	369.0452	163.0397	1.000
MSDial	295	295	90	90	neg_126	neg_213	Pearson correlation	$[M-H]^-$	$[M-H]^-$	3.128	3.121	133.0140	163.0397	0.982

MSDial	295	295	90	90	neg_1672	neg_213	found in higher mz's MSMS	$[M+Na-2H]^-$	$[M-H]^-$	3.127	3.121	581.0903	163.0397	1.000
MSDial	295	295	90	90	neg_1549	neg_213	found in higher mz's MSMS	$[M-H]^-$	$[M-H]^-$	3.087	3.121	530.1289	163.0397	1.000
MSDial	295	295	90	90	neg_1549	neg_367	found in higher mz's MSMS	$[M-H]^-$	$[M-H]^-$	3.087	3.071	530.1289	206.0817	1.000
MSDial	295	295	90	90	neg_126	neg_434	similar chromatogram in higher mz	$[M-H]^-$	$[M-H]^-$	3.128	3.133	133.0140	224.0561	1.000
MSDial	295	295	90	90	neg_213	neg_659	Pearson correlation	$[M-H]^-$	$[M-H]^-$	3.121	3.121	163.0397	279.0504	0.992
MSDial	295	295	90	90	neg_213	neg_659	found in higher mz's MSMS	$[M-H]^-$	$[M-H]^-$	3.121	3.121	163.0397	279.0504	1.000
MSDial	295	295	90	90	neg_1067	neg_659	found in higher mz's MSMS	$[M+Na-2H]^-$	$[M-H]^-$	3.125	3.121	369.0452	279.0504	1.000
MSDial	295	295	90	90	neg_1067	neg_659	Pearson correlation	$[M+Na-2H]^-$	$[M-H]^-$	3.125	3.121	369.0452	279.0504	0.908
MSDial	295	295	90	90	neg_1549	neg_659	found in higher mz's MSMS	$[M-H]^-$	$[M-H]^-$	3.087	3.121	530.1289	279.0504	1.000
Adducts/Neutral losses	295	295	370	90	pos_1647	neg_659	pol / adduct	$[M+NH_4]^+$	$[M-H]^-$	3.125	3.121	298.0920	279.0504	0.974
MSDial	295	295	90	90	neg_126	neg_659	Pearson correlation	$[M-H]^-$	$[M-H]^-$	3.128	3.121	133.0140	279.0504	0.992
MSDial	295	295	90	90	neg_659	neg_979	Pearson correlation	$[M-H]^-$	$[M-H]^-$	3.121	3.128	279.0504	347.0374	0.944
MSDial	295	295	90	90	neg_1067	neg_979	pol / adduct	$[M+Na-2H]^-$	$[M-H]^-$	3.125	3.128	369.0452	347.0374	1.000
MSDial	295	295	90	90	neg_1067	neg_979	Pearson correlation	$[M+Na-2H]^-$	$[M-H]^-$	3.125	3.128	369.0452	347.0374	0.904
MSDial	295	295	90	90	neg_659	neg_979	found in higher mz's MSMS	$[M-H]^-$	$[M-H]^-$	3.121	3.128	279.0504	347.0374	1.000
MSDial	295	295	90	90	neg_213	neg_979	found in higher mz's MSMS	$[M-H]^-$	$[M-H]^-$	3.121	3.128	163.0397	347.0374	1.000
MSDial	295	295	90	90	neg_126	neg_979	found in higher mz's MSMS	$[M-H]^-$	$[M-H]^-$	3.128	3.128	133.0140	347.0374	1.000
MSDial	295	295	90	90	neg_213	neg_979	Pearson correlation	$[M-H]^-$	$[M-H]^-$	3.121	3.128	163.0397	347.0374	0.922
MSDial	295	295	90	90	neg_126	neg_979	Pearson correlation	$[M-H]^-$	$[M-H]^-$	3.128	3.128	133.0140	347.0374	0.940
MSDial	295	295	370	370	pos_1647	pos_2225	Pearson correlation	$[M+NH_4]^+$	$[M+H]^+$	3.125	3.128	298.0920	351.0375	0.910
MSDial	295	295	370	370	pos_1647	pos_3760	Pearson correlation	$[M+NH_4]^+$	$[M+NH_4]^+$	3.125	3.124	298.0920	578.1494	0.931
Adducts/Neutral losses	296	296	371	27	pos_225	neg_120	pol / adduct	$[M+H]^+$	$[M-H]^-$	0.658	0.620	134.0445	132.0299	0.963
MSDial	305	305	381	381	pos_2192	pos_239	similar chromatogram in higher mz	$[M+H]^+$	$[M+H]^+$	0.852	0.869	348.0700	136.0616	1.000
MSDial	305	305	381	381	pos_2192	pos_239	found in higher mz's MSMS	$[M+H]^+$	$[M+H]^+$	0.852	0.869	348.0700	136.0616	1.000

Adducts/Neutral losses	308	308	384	9	pos_2422	neg_1059	pol / adduct	$[M+H]^+$	[M-H]-	2.607	2.601	369.1647	367.1499	0.851
MSDial	316	316	391	391	pos_2231	pos_254	found in higher mz's MSMS	$[M+H]^+$	$[M+H]^+$	1.364	1.383	351.1732	138.0913	1.000
Adducts/Neutral losses	318	318	393	297	pos_2600	neg_971	pol / adduct	$[M+Na]^+$	$[M-H_2O-H]^-$	4.862	4.872	387.1926	345.1827	0.066
Adducts/Neutral losses	322	322	396	396	pos_2619	pos_2451	neutral loss	H_3N	H_3N	5.904	5.905	388.3407	371.3149	0.972
MSDial	322	322	396	396	pos_2451	pos_2619	Pearson correlation	$[M+H]^+$	$[M+NH_4]^+$	5.905	5.904	371.3149	388.3407	0.972
MSDial	322	322	396	396	pos_2451	pos_2619	pol / adduct	$[M+H]^+$	$[M+NH_4]^+$	5.905	5.904	371.3149	388.3407	1.000
MSDial	322	322	396	396	pos_2619	pos_2871	similar chromatogram in higher mz	$[M+NH_4]^+$	$[M+H]^+$	5.904	5.913	388.3407	421.3603	1.000
MSDial	322	322	396	396	pos_2451	pos_3219	similar chromatogram in higher mz	$[M+H]^+$	$[M+2H]^{2+}$	5.905	5.916	371.3149	469.3609	1.000
Adducts/Neutral losses	323	323	397	196	pos_2631	neg_1074	pol / adduct	$[M+NH_4]^+$	$[M-H]^-$	3.018	3.020	390.1379	371.0974	0.982
MSDial	323	323	196	196	neg_1379	neg_317	similar chromatogram in higher mz	$[M-H]^-$	$[M-H]^-$	3.016	3.020	461.0928	195.0293	1.000
MSDial	323	323	196	196	neg_1074	neg_317	similar chromatogram in higher mz	$[M-H]^-$	$[M-H]^-$	3.020	3.020	371.0974	195.0293	1.000
MSDial	325	325	400	400	pos_2503	pos_2686	similar chromatogram in higher mz	$[M+H]^+$	$[M+H]^+$	6.228	6.233	376.2963	397.4136	1.000
Adducts/Neutral losses	329	329	380	278	pos_2160	neg_1033	pol / adduct	$[M+NH_4]^+$	$[M+Cl]^-$	2.637	2.634	344.1327	361.0689	0.854
Adducts/Neutral losses	329	329	380	278	pos_2380	neg_1033	pol / adduct	$[M+K]^+$	$[M+Cl]^-$	2.640	2.634	365.0620	361.0689	0.801
MSDial	329	329	278	278	neg_1033	neg_1314	Pearson correlation	$[M+Cl]^-$	$[M+Na-2H]^-$	2.634	2.679	361.0689	439.0850	0.945
Adducts/Neutral losses	329	329	380	278	pos_2790	neg_1361	pol / adduct	$[M+H]^+$	$[M+FA-H]^-$	2.637	2.637	411.0591	455.0500	0.882
Adducts/Neutral losses	329	329	398	278	pos_4082	neg_1784	pol / adduct	$[M+Na]^+$	$[M-H]^-$	2.677	2.634	675.1902	651.1919	0.816
Adducts/Neutral losses	329	329	398	278	pos_4082	neg_1808	pol / adduct	$[M+Na]^+$	$[M+Na-2H]^-$	2.677	2.629	675.1902	673.1735	0.757
MSDial	329	329	278	278	neg_1784	neg_1808	pol / adduct	$[M-H]^-$	$[M+Na-2H]^-$	2.634	2.629	651.1919	673.1735	1.000
MSDial	329	329	278	278	neg_1784	neg_1808	Pearson correlation	$[M-H]^-$	$[M+Na-2H]^-$	2.634	2.629	651.1919	673.1735	0.940
MSDial	329	329	278	278	neg_710	neg_872	Pearson correlation	$[M+Cl]^-$	$[M-H]^-$	2.615	2.634	292.0819	325.0922	0.912

MSDial	329	329	278	278	neg_1033	neg_872	pol / adduct	[M+Cl]-	[M-H]-	2.634	2.634	361.0689	325.0922	1.000
MSDial	329	329	278	278	neg_1784	neg_872	Pearson correlation	[M-H]-	[M-H]-	2.634	2.634	651.1919	325.0922	0.908
Adducts/Neutral losses	329	329	380	278	pos_2380	neg_872	pol / adduct	[M+K]+	[M-H]-	2.640	2.634	365.0620	325.0922	0.911
MSDial	329	329	278	278	neg_1361	neg_872	Pearson correlation	[M-H]-	[M-H]-	2.637	2.634	455.0500	325.0922	0.903
Adducts/Neutral losses	329	329	380	278	pos_2160	neg_872	pol / adduct	$[M+NH_4]^+$	[M-H]-	2.637	2.634	344.1327	325.0922	0.943
MSDial	329	329	380	380	pos_2160	pos_2380	pol / adduct	$[M+NH_4]^+$	[M+K]+	2.637	2.640	344.1327	365.0620	1.000
MSDial	329	329	380	380	pos_2160	pos_2380	Pearson correlation	$[M+NH_4]^+$	[M+K]+	2.637	2.640	344.1327	365.0620	0.933
MSDial	329	329	398	398	pos_208	pos_2633	similar chromatogram in higher mz	[M+H]+	$[M+NH_4]^+$	2.683	2.687	130.1587	390.1745	1.000
MSDial	329	329	380	380	pos_2160	pos_2790	Pearson correlation	$[M+NH_4]^+$	[M+Na]+	2.637	2.637	344.1327	411.0591	0.928
MSDial	329	329	398	398	pos_208	pos_4082	similar chromatogram in higher mz	[M+H]+	[M+Na]+	2.683	2.677	130.1587	675.1902	1.000
Adducts/Neutral losses	332	332	407	188	pos_2804	neg_1220	pol / adduct	[M+H]+	[M-H]-	2.548	2.553	413.1397	411.1261	0.916
Adducts/Neutral losses	332	332	188	188	neg_1452	neg_1341	neutral loss	CO_2	CO_2	2.506	2.504	491.1762	447.1862	0.928
Adducts/Neutral losses	332	332	415	188	pos_2898	neg_1341	pol / adduct	[M+Na]+	[M+FA-H]-	2.508	2.504	425.1763	447.1862	0.904
MSDial	332	332	188	188	neg_1341	neg_1452	Pearson correlation	[M-H]-	[M-H]-	2.504	2.506	447.1862	491.1762	0.928
MSDial	332	332	188	188	neg_1220	neg_1708	Pearson correlation	[M-H]-	[M-H]-	2.553	2.518	411.1261	605.1713	0.911
MSDial	332	332	188	188	neg_1452	neg_1708	Pearson correlation	[M-H]-	[M-H]-	2.506	2.518	491.1762	605.1713	0.910
MSDial	332	332	188	188	neg_1341	neg_1708	Pearson correlation	[M-H]-	[M-H]-	2.504	2.518	447.1862	605.1713	0.945
MSDial	332	332	407	407	pos_2329	pos_2804	similar chromatogram in higher mz	$[M+NH_4]^+$	[M+H]+	2.548	2.548	360.1647	413.1397	1.000
Adducts/Neutral losses	337	337	154	154	neg_1345	neg_1206	neutral loss	CO_2	CO_2	3.207	3.206	449.0330	405.0427	0.904
Adducts/Neutral losses	337	337	412	154	pos_2338	neg_1206	pol / adduct	[M+H]+	[M+FA-H]-	3.198	3.206	361.0512	405.0427	0.921
Adducts/Neutral losses	337	337	154	154	neg_1889	neg_1206	neutral loss	disacch.-H_2O	disacch.-H_2O	3.243	3.206	745.1458	405.0427	0.770

Adducts/Neutral losses	337	337	403	154	pos_2739	neg_1287	pol / adduct	$[M+NH_4]^+$	$[M+FA-H]^-$	3.204	3.200	404.2272	431.1911	0.938
MSDial	337	337	154	154	neg_1206	neg_1345	Pearson correlation	$[M+Na-2H]^-$	$[M+Cl]^-$	3.206	3.207	405.0427	449.0330	0.904
Adducts/Neutral losses	337	337	516	154	pos_4094	neg_1788	pol / adduct	$[M+Na]^+$	$[M-H]^-$	3.240	3.242	679.1466	655.1503	0.925
Adducts/Neutral losses	337	337	154	154	neg_1886	neg_1788	neutral loss	malonyl-H_2O	malonyl-H_2O	3.260	3.242	741.1509	655.1503	0.679
Adducts/Neutral losses	337	337	523	154	pos_4126	neg_1810	pol / adduct	$[M+NH_4]^+$	$[M-H]^-$	3.197	3.197	694.1591	675.1189	0.966
MSDial	337	337	154	154	neg_1788	neg_1830	found in higher mz's MSMS	$[M-H]^-$	$[M+Cl]^-$	3.242	3.242	655.1503	691.1270	1.000
MSDial	337	337	154	154	neg_1788	neg_1830	pol / adduct	$[M-H]^-$	$[M+Cl]^-$	3.242	3.242	655.1503	691.1270	1.000
Adducts/Neutral losses	337	337	516	154	pos_4094	neg_1830	pol / adduct	$[M+Na]^+$	$[M+Cl]^-$	3.240	3.242	679.1466	691.1270	0.904
MSDial	337	337	154	154	neg_1788	neg_1830	Pearson correlation	$[M-H]^-$	$[M+Cl]^-$	3.242	3.242	655.1503	691.1270	0.978
MSDial	337	337	154	154	neg_1810	neg_1840	pol / adduct	$[M-H]^-$	$[M+Na-2H]^-$	3.197	3.197	675.1189	697.1003	1.000
MSDial	337	337	154	154	neg_1810	neg_1840	Pearson correlation	$[M-H]^-$	$[M+Na-2H]^-$	3.197	3.197	675.1189	697.1003	0.935
Adducts/Neutral losses	337	337	523	154	pos_4126	neg_1840	pol / adduct	$[M+NH_4]^+$	$[M+Na-2H]^-$	3.197	3.197	694.1591	697.1003	0.909
MSDial	337	337	154	154	neg_1830	neg_1865	Pearson correlation	$[M+Cl]^-$	$[M-H]^-$	3.242	3.244	691.1270	718.1454	0.926
MSDial	337	337	154	154	neg_1788	neg_1865	Pearson correlation	$[M-H]^-$	$[M-H]^-$	3.242	3.244	655.1503	718.1454	0.917
MSDial	337	337	154	154	neg_1830	neg_1869	Pearson correlation	$[M+Cl]^-$	$[M-H]^-$	3.242	3.242	691.1270	723.1379	0.932
MSDial	337	337	154	154	neg_1788	neg_1869	found in higher mz's MSMS	$[M-H]^-$	$[M-H]^-$	3.242	3.242	655.1503	723.1379	1.000
MSDial	337	337	154	154	neg_1788	neg_1869	Pearson correlation	$[M-H]^-$	$[M-H]^-$	3.242	3.242	655.1503	723.1379	0.909
Adducts/Neutral losses	337	337	154	154	neg_1886	neg_1869	neutral loss	H_2O	H_2O	3.260	3.242	741.1509	723.1379	0.631
MSDial	337	337	154	154	neg_1206	neg_1869	Pearson correlation	$[M+Na-2H]^-$	$[M-H]^-$	3.206	3.242	405.0427	723.1379	0.910
MSDial	337	337	154	154	neg_1287	neg_1870	Pearson correlation	$[M-H]^-$	$[M-H]^-$	3.200	3.200	431.1911	723.2496	0.915
MSDial	337	337	154	154	neg_1788	neg_1870	found in higher mz's MSMS	$[M-H]^-$	$[M-H]^-$	3.242	3.200	655.1503	723.2496	1.000
MSDial	337	337	154	154	neg_1869	neg_1886	pol / adduct	$[M-H]^-$	$[M-H]^-$	3.242	3.260	723.1379	741.1509	1.000

MSDial	337	337	154	154	neg_1788	neg_1889	Pearson correlation	[M-H]$^-$	[M+Na-2H]$^-$	3.242	3.243	655.1503	745.1458	0.936
MSDial	337	337	154	154	neg_1788	neg_1889	found in higher mz's MSMS	[M-H]$^-$	[M+Na-2H]$^-$	3.242	3.243	655.1503	745.1458	1.000
MSDial	337	337	154	154	neg_1869	neg_1889	pol / adduct	[M-H]$^-$	[M+Na-2H]$^-$	3.242	3.243	723.1379	745.1458	1.000
MSDial	337	337	154	154	neg_1830	neg_1889	Pearson correlation	[M+Cl]$^-$	[M+Na-2H]$^-$	3.242	3.243	691.1270	745.1458	0.928
MSDial	337	337	154	154	neg_1019	neg_3	found in higher mz's MSMS	[M-H]$^-$	[M-H]$^-$	3.166	3.203	357.1185	59.0115	1.000
MSDial	337	337	154	154	neg_1345	neg_3	found in higher mz's MSMS	[M+Cl]$^-$	[M-H]$^-$	3.207	3.203	449.0330	59.0115	1.000
MSDial	337	337	154	154	neg_1788	neg_311	similar chromatogram in higher mz	[M-H]-	[M-H]$^-$	3.242	3.233	655.1503	193.0501	1.000
Adducts/Neutral losses	337	337	573	154	pos_659	neg_311	pol / adduct	[M+H]$^+$	[M-H]$^-$	3.226	3.233	195.0649	193.0501	0.896
MSDial	337	337	154	154	neg_3	neg_646	similar chromatogram in higher mz	[M-H]$^-$	[M-H]$^-$	3.203	3.204	59.0115	277.0348	1.000
MSDial	337	337	154	154	neg_3	neg_646	Pearson correlation	[M-H]$^-$	[M-H]$^-$	3.203	3.204	59.0115	277.0348	0.967
MSDial	337	337	154	154	neg_1206	neg_646	found in higher mz's MSMS	[M+Na-2H]$^-$	[M-H]$^-$	3.206	3.204	405.0427	277.0348	1.000
MSDial	337	337	154	154	neg_1840	neg_646	Pearson correlation	[M+Na-2H]$^-$	[M-H]$^-$	3.197	3.204	697.1003	277.0348	0.929
MSDial	337	337	154	154	neg_1345	neg_646	found in higher mz's MSMS	[M+Cl]$^-$	[M-H]$^-$	3.207	3.204	449.0330	277.0348	1.000
MSDial	337	337	154	154	neg_1810	neg_646	Pearson correlation	[M-H]$^-$	[M-H]$^-$	3.197	3.204	675.1189	277.0348	0.928
MSDial	337	337	154	154	neg_1840	neg_646	found in higher mz's MSMS	[M+Na-2H]$^-$	[M-H]$^-$	3.197	3.204	697.1003	277.0348	1.000
MSDial	337	337	412	412	pos_2338	pos_2507	Pearson correlation	[M+Na]$^+$	[M+K]+	3.198	3.198	361.0512	377.0261	0.943
MSDial	337	337	412	412	pos_2338	pos_2882	Pearson correlation	[M+Na]$^+$	[M+H]$^+$	3.198	3.202	361.0512	423.0227	0.908
MSDial	337	337	412	412	pos_2777	pos_2882	Pearson correlation	[M+H]$^+$	[M+H]$^+$	3.205	3.202	409.0430	423.0227	0.926
MSDial	337	337	516	516	pos_2190	pos_4094	pol / adduct	$[M+NH_4]^{2+}$	[M+Na]$^+$	3.242	3.240	348.0591	679.1466	1.000
Adducts/Neutral losses	338	338	414	288	pos_1925	neg_922	pol / adduct	[M+Na]$^+$	[M+Cl]$^-$	2.745	2.745	324.1059	336.0848	0.887
Adducts/Neutral losses	338	338	414	299	pos_1925	neg_976	pol / adduct	[M+Na]$^+$	[M+FA-H]$^-$	2.745	2.737	324.1059	346.1136	0.725
MSDial	338	338	414	414	pos_1925	pos_2861	Pearson correlation	[M+Na]$^+$	$[M+NH_4]^+$	2.745	2.738	324.1059	420.2223	0.909
MSDial	338	338	414	414	pos_2861	pos_2897	Pearson correlation	$[M+NH_4]^+$	[M+Na]$^+$	2.738	2.742	420.2223	425.1762	0.912
MSDial	338	338	414	414	pos_2861	pos_2897	pol / adduct	$[M+NH_4]^+$	[M+Na]$^+$	2.738	2.742	420.2223	425.1762	1.000

MSDial	340	340	419	419	pos_2643	pos_2931	pol / adduct	$[M+H]^+$	$[M+K]^+$	5.899	5.897	391.2841	429.2398	1.000
MSDial	340	340	419	419	pos_2643	pos_3203	Pearson correlation	$[M+H]^+$	$[M+CH_3OH+H]^+$	5.899	5.901	391.2841	466.3518	0.903
Adducts/Neutral losses	343	343	420	420	pos_301	pos_200	neutral loss	H_3N	H_3N	0.547	0.551	146.1649	129.1383	0.976
MSDial	343	343	420	420	pos_200	pos_301	Pearson correlation	$[M+H]^+$	$[M+H]^+$	0.551	0.547	129.1383	146.1649	0.976
MSDial	343	343	420	420	pos_200	pos_301	pol / adduct	$[M+H]^+$	$[M+H]^+$	0.551	0.547	129.1383	146.1649	1.000
MSDial	343	343	420	420	pos_294	pos_301	found in higher mz's MSMS	$[M+H]^+$	$[M+H]^+$	0.553	0.547	146.0297	146.1649	1.000
MSDial	343	343	420	420	pos_200	pos_301	found in higher mz's MSMS	$[M+H]^+$	$[M+H]^+$	0.551	0.547	129.1383	146.1649	1.000
Adducts/Neutral losses	347	347	571	200	pos_307	neg_153	pol / adduct	$[M+H]^+$	$[M-H]^-$	0.647	0.618	147.0763	145.0615	0.915
Adducts/Neutral losses	347	347	571	200	pos_203	neg_156	pol / adduct	$[M+H-H_2O]^+$	$[M-H]^-$	0.648	0.634	130.0496	146.0455	0.871
Adducts/Neutral losses	347	347	200	200	neg_366	neg_156	neutral loss	$C_2H_4O_2$	$C_2H_4O_2$	0.656	0.634	206.0664	146.0455	0.900
Adducts/Neutral losses	347	347	571	200	pos_588	neg_156	pol / adduct	$[M+K]^+$	$[M-H]^-$	0.630	0.634	186.0157	146.0455	0.844
MSDial	347	347	200	200	neg_153	neg_156	found in higher mz's MSMS	$[M-H]^-$	$[M-H]^-$	0.618	0.634	145.0615	146.0455	1.000
Adducts/Neutral losses	347	347	571	200	pos_308	neg_156	pol / adduct	$[M+H]^+$	$[M-H]^-$	0.652	0.634	148.0598	146.0455	0.922
Adducts/Neutral losses	347	347	200	200	neg_740	neg_156	neutral loss	$C_7H_5O_4$	$C_7H_5O_4$	0.667	0.634	299.0611	146.0455	0.865
Adducts/Neutral losses	347	347	556	200	pos_411	neg_366	pol / adduct	$[M+H]^+$	$[M+FA-H]^-$	0.687	0.656	162.0757	206.0664	0.626
MSDial	347	347	200	200	neg_156	neg_366	Pearson correlation	$[M-H]^-$	$[M-H]^-$	0.634	0.656	146.0455	206.0664	0.900
MSDial	347	347	200	200	neg_156	neg_377	found in higher mz's MSMS	$[M-H]^-$	$[M-H]^-$	0.634	0.633	146.0455	208.0568	1.000
MSDial	347	347	200	200	neg_366	neg_740	Pearson correlation	$[M-H]^-$	$[M+Cl]^-$	0.656	0.667	206.0664	299.0611	0.929
Adducts/Neutral losses	347	347	571	571	pos_307	pos_203	neutral loss	H_3N	H_3N	0.647	0.648	147.0763	130.0496	0.997
Adducts/Neutral losses	347	347	571	571	pos_308	pos_203	neutral loss	H_2O	H_2O	0.652	0.648	148.0598	130.0496	0.878

MSDial	347	347	571	571	pos_1518	pos_253	Pearson correlation	$[M+CH_3OH+H]^+$	$[M+H]^+$	0.703	0.677	287.1223	138.0548	0.931
MSDial	347	347	571	571	pos_203	pos_307	Pearson correlation	$[M+H]^+$	$[M+H]^+$	0.648	0.647	130.0496	147.0763	0.997
MSDial	347	347	571	571	pos_203	pos_307	found in higher mz's MSMS	$[M+H]^+$	$[M+H]^+$	0.648	0.647	130.0496	147.0763	1.000
MSDial	347	347	571	571	pos_203	pos_308	found in higher mz's MSMS	$[M+H]^+$	$[M+H]^+$	0.648	0.652	130.0496	148.0598	1.000
MSDial	347	347	571	571	pos_1135	pos_308	Pearson correlation	$[M+K]^+$	$[M+H]^+$	0.607	0.652	252.0453	148.0598	0.932
MSDial	347	347	556	556	pos_224	pos_411	Pearson correlation	$[M+H]^+$	$[M+H]^+$	0.647	0.687	133.0604	162.0757	0.906
MSDial	347	347	556	556	pos_224	pos_473	Pearson correlation	$[M+H]^+$	$[M+K]^+$	0.647	0.628	133.0604	171.0163	0.925
MSDial	347	347	556	556	pos_224	pos_473	pol / adduct	$[M+H]^+$	$[M+K]^+$	0.647	0.628	133.0604	171.0163	1.000
MSDial	347	347	571	571	pos_308	pos_588	Pearson correlation	$[M+H]^+$	$[M+K]^+$	0.652	0.630	148.0598	186.0157	0.951
MSDial	347	347	571	571	pos_308	pos_588	pol / adduct	$[M+H]^+$	$[M+K]^+$	0.652	0.630	148.0598	186.0157	1.000
MSDial	347	347	571	571	pos_143	pos_855	Pearson correlation	$[M+H]^+$	$[M+H-H_2O]^+$	0.647	0.647	118.0860	221.1857	0.949
MSDial	347	347	571	571	pos_308	pos_855	Pearson correlation	$[M+H]^+$	$[M+H-H_2O]^+$	0.652	0.647	148.0598	221.1857	0.903
MSDial	347	347	571	571	pos_253	pos_855	Pearson correlation	$[M+H]^+$	$[M+H-H_2O]^+$	0.677	0.647	138.0548	221.1857	0.943
MSDial	347	347	571	571	pos_1135	pos_94	Pearson correlation	$[M+K]^+$	$[M]^+$	0.607	0.647	252.0453	104.1066	0.915
MSDial	347	347	571	571	pos_588	pos_94	Pearson correlation	$[M+K]^+$	$[M]^+$	0.630	0.647	186.0157	104.1066	0.905
MSDial	347	347	571	571	pos_855	pos_94	found in higher mz's MSMS	$[M+H-H_2O]^+$	$[M]^+$	0.647	0.647	221.1857	104.1066	1.000
MSDial	347	347	571	571	pos_308	pos_94	Pearson correlation	$[M+H]^+$	$[M]^+$	0.652	0.647	148.0598	104.1066	0.949
MSDial	347	347	571	571	pos_855	pos_94	Pearson correlation	$[M+H-H_2O]^+$	$[M]^+$	0.647	0.647	221.1857	104.1066	0.932
Adducts/Neutral losses	351	351	431	153	pos_3101	neg_1290	pol / adduct	$[M+NH_4]^+$	$[M-H]^-$	4.430	4.431	450.2675	431.2275	0.994
Adducts/Neutral losses	351	351	431	153	pos_3135	neg_1290	pol / adduct	$[M+Na]^+$	$[M-H]^-$	4.428	4.431	455.2241	431.2275	0.996
Adducts/Neutral losses	351	351	544	153	pos_4187	neg_1842	pol / adduct	$[M+NH_4]^+$	$[M-H]^-$	4.433	4.434	716.3375	697.2957	0.625
MSDial	351	351	153	153	neg_1842	neg_1864	similar chromatogram in higher mz	$[M-2H]^{2-}$	$[M-2H]^{2-}$	4.434	4.436	697.2957	715.2867	1.000
MSDial	351	351	153	153	neg_1290	neg_1864	similar chromatogram in higher mz	$[M-H]^-$	$[M-2H]^{2-}$	4.431	4.436	431.2275	715.2867	1.000
MSDial	351	351	153	153	neg_1842	neg_1864	Pearson correlation	$[M-2H]^{2-}$	$[M-2H]^{2-}$	4.434	4.436	697.2957	715.2867	0.903

MSDial	351	351	153	153	neg_1842	neg_1867	similar chromatogram in higher mz	$[M-2H]^{2-}$	$[M-2H]^{2-}$	4.434	4.434	697.2957	720.3015	1.000
MSDial	351	351	153	153	neg_1864	neg_1867	Pearson correlation	$[M-2H]^{2-}$	$[M-2H]^{2-}$	4.436	4.434	715.2867	720.3015	0.908
MSDial	351	351	153	153	neg_1842	neg_1867	Pearson correlation	$[M-2H]^{2-}$	$[M-2H]^{2-}$	4.434	4.434	697.2957	720.3015	0.964
MSDial	351	351	431	431	pos_3101	pos_3135	Pearson correlation	$[M+H]^+$	$[M+H]^+$	4.430	4.428	450.2675	455.2241	0.999
Adducts/Neutral losses	354	354	435	43	pos_3211	neg_1348	pol / adduct	$[M+NH_4]^+$	$[M-H]^-$	3.486	3.485	468.2438	449.2019	0.978
Adducts/Neutral losses	354	354	435	43	pos_3242	neg_1348	pol / adduct	$[M+Na]^+$	$[M-H]^-$	3.486	3.485	473.1985	449.2019	0.959
MSDial	354	354	435	435	pos_1546	pos_3211	found in higher mz's MSMS	$[M+NH_4]^+$	$[M+NH_4]^+$	3.486	3.486	289.1633	468.2438	1.000
MSDial	354	354	435	435	pos_1546	pos_3211	Pearson correlation	$[M+NH_4]^+$	$[M+NH_4]^+$	3.486	3.486	289.1633	468.2438	0.965
MSDial	354	354	435	435	pos_1546	pos_3242	Pearson correlation	$[M+NH_4]^+$	$[M-H]^-$	3.486	3.486	289.1633	473.1985	0.936
MSDial	354	354	435	435	pos_3211	pos_3242	Pearson correlation	$[M+NH_4]^+$	$[M+H]^+$	3.486	3.486	468.2438	473.1985	0.974
MSDial	354	354	435	435	pos_1546	pos_3979	Pearson correlation	$[M+NH_4]^+$	$[2M+H]^+$	3.486	3.493	289.1633	643.3672	0.929
MSDial	354	354	435	435	pos_3211	pos_3979	Pearson correlation	$[M+NH_4]^+$	$[2M+H]^+$	3.486	3.493	468.2438	643.3672	0.947
Adducts/Neutral losses	361	361	446	72	pos_3403	neg_1547	pol / adduct	$[M+H]^+$	$[M+Cl]^-$	3.777	3.791	495.3319	529.2924	0.929
MSDial	361	361	446	446	pos_3161	pos_3403	Pearson correlation	$[M+H]^+$	$[M+H]^+$	3.780	3.777	459.3094	495.3319	0.958
MSDial	361	361	446	446	pos_3161	pos_3403	found in higher mz's MSMS	$[M+H]^+$	$[M+H]^+$	3.780	3.777	459.3094	495.3319	1.000
Adducts/Neutral losses	362	362	447	82	pos_3406	neg_1581	pol / adduct	$[M+H]^+$	$[M+FA-H]^-$	5.615	5.617	496.3395	540.3294	0.983
MSDial	364	364	449	449	pos_2103	pos_3432	Pearson correlation	$[M+H]^+$	$[M+2H]^{2+}$	3.307	3.306	339.1069	502.1028	0.906
MSDial	364	364	449	449	pos_3432	pos_835	similar chromatogram in higher mz	$[M+2H]^{2+}$	$[M+H]^+$	3.306	3.313	502.1028	219.1008	1.000
Adducts/Neutral losses	365	365	455	75	pos_3441	neg_1542	pol / adduct	$[M+Na]^+$	$[M+FA-H]^-$	3.589	3.587	503.2972	525.3058	0.953
Adducts/Neutral losses	365	365	455	75	pos_3312	neg_1542	pol / adduct	$[M+H]^+$	$[M+FA-H]^-$	3.587	3.587	481.3145	525.3058	0.975
Adducts/Neutral losses	365	365	455	75	pos_3518	neg_1554	pol / adduct	$[M+Na]^+$	$[M+Cl]^-$	3.580	3.584	519.2903	531.2719	0.911
MSDial	365	365	75	75	neg_1542	neg_1554	Pearson correlation	$[M+FA-H]^-$	$[M-H]^-$	3.587	3.584	525.3058	531.2719	0.971

MSDial	365	365	75	75	neg_1554	neg_1697	Pearson correlation	[M-H]$^-$	[M+K-2H]$^-$	3.584	3.586	531.2719	593.2930	0.920
MSDial	365	365	75	75	neg_1542	neg_1697	Pearson correlation	[M+FA-H]$^-$	[M+K-2H]$^-$	3.587	3.586	525.3058	593.2930	0.952
MSDial	365	365	455	455	pos_2187	pos_3312	Pearson correlation	[M+H]$^+$	[M+H]$^+$	3.588	3.587	347.2214	481.3145	0.969
MSDial	365	365	455	455	pos_3312	pos_3441	pol / adduct	[M+H]$^+$	[M+Na]$^+$	3.587	3.589	481.3145	503.2972	1.000
Adducts/Neutral losses	365	365	455	455	pos_3518	pos_3441	neutral loss	O	O	3.580	3.589	519.2903	503.2972	0.910
MSDial	365	365	455	455	pos_2187	pos_3441	Pearson correlation	[M+H]$^+$	[M+Na]$^+$	3.588	3.589	347.2214	503.2972	0.954
MSDial	365	365	455	455	pos_3312	pos_3441	Pearson correlation	[M+H]$^+$	[M+Na]$^+$	3.587	3.589	481.3145	503.2972	0.957
MSDial	365	365	455	455	pos_2187	pos_3518	Pearson correlation	[M+H]$^+$	[M+K]$^+$	3.588	3.580	347.2214	519.2903	0.948
MSDial	365	365	455	455	pos_3441	pos_3518	Pearson correlation	[M+Na]$^+$	[M+K]$^+$	3.589	3.580	503.2972	519.2903	0.910
MSDial	365	365	455	455	pos_3312	pos_3518	Pearson correlation	[M+H]$^+$	[M+K]$^+$	3.587	3.580	481.3145	519.2903	0.948
MSDial	365	365	455	455	pos_3441	pos_3719	Pearson correlation	[M+Na]$^+$	[M+H]$^+$	3.589	3.588	503.2972	565.2661	0.924
MSDial	366	366	101	101	neg_1427	neg_1500	Pearson correlation	[M+K-2H]$^-$	[M-H]-	5.147	5.144	479.2404	505.2563	0.903
Adducts/Neutral losses	366	366	459	101	pos_3572	neg_1500	pol / adduct	[M+Na]$^+$	[M-H]-	5.149	5.144	529.2526	505.2563	0.956
Adducts/Neutral losses	366	366	450	101	pos_3351	neg_1555	pol / adduct	[M+H]$^+$	[M+FA-H]$^-$	5.154	5.154	487.2885	531.2800	0.960
MSDial	366	366	101	101	neg_1187	neg_1555	similar chromatogram in higher mz	[M-H]$^-$	[M+FA-H]$^-$	5.142	5.154	397.2254	531.2800	1.000
MSDial	366	366	101	101	neg_1427	neg_1555	similar chromatogram in higher mz	[M+K-2H]$^-$	[M+FA-H]$^-$	5.147	5.154	479.2404	531.2800	1.000
MSDial	366	366	101	101	neg_1500	neg_1555	similar chromatogram in higher mz	[M-H]$^-$	[M+FA-H]$^-$	5.144	5.154	505.2563	531.2800	1.000
Adducts/Neutral losses	366	366	450	101	pos_3447	neg_1555	pol / adduct	[M+NH$_4$]$^+$	[M+FA-H]$^-$	5.151	5.154	504.3163	531.2800	0.984
MSDial	366	366	101	101	neg_1427	neg_1664	similar chromatogram in higher mz	[M+K-2H]$^-$	[M-H]$^-$	5.147	5.146	479.2404	573.2432	1.000
MSDial	366	366	101	101	neg_1500	neg_1664	similar chromatogram in higher mz	[M-H]$^-$	[M-H]$^-$	5.144	5.146	505.2563	573.2432	1.000
MSDial	366	366	101	101	neg_1500	neg_1664	Pearson correlation	[M-H]$^-$	[M-H]$^-$	5.144	5.146	505.2563	573.2432	0.950
MSDial	366	366	101	101	neg_1187	neg_1664	similar chromatogram in higher mz	[M-H]$^-$	[M-H]$^-$	5.142	5.146	397.2254	573.2432	1.000
MSDial	366	366	101	101	neg_1555	neg_1702	Pearson correlation	[M+FA-H]$^-$	[M-H]$^-$	5.154	5.157	531.2800	599.2676	0.979

Adducts/Neutral losses	366	366	450	450	pos_3447	pos_3351	neutral loss	H_3N	H_3N	5.151	5.154	504.3163	487.2885	0.965
MSDial	366	366	450	450	pos_3351	pos_3447	Pearson correlation	$[M+H]^+$	$[M+NH_4]^+$	5.154	5.151	487.2885	504.3163	0.965
MSDial	366	366	450	450	pos_3351	pos_3447	pol / adduct	$[M+H]^+$	$[M+NH_4]^+$	5.154	5.151	487.2885	504.3163	1.000
Adducts/Neutral losses	368	368	451	84	pos_3472	neg_1588	pol / adduct	$[M+H]^+$	$[M+Cl]^-$	3.501	3.507	509.1277	543.0903	0.883
Adducts/Neutral losses	370	370	453	69	pos_3503	neg_1520	pol / adduct	$[M+H]^+$	$[M-H]^-$	2.212	2.213	516.2292	514.2143	0.862
Adducts/Neutral losses	372	372	429	177	pos_3123	neg_1282	pol / adduct	$[M+Na]^+$	$[M-H]^-$	3.713	3.715	453.1722	429.1755	0.891
Adducts/Neutral losses	372	372	457	177	pos_3462	neg_1495	pol / adduct	$[M+H]^+$	$[M-H]^-$	3.713	3.712	507.1125	505.0978	0.949
Adducts/Neutral losses	372	372	463	177	pos_3598	neg_1526	pol / adduct	$[M+NH_4]^+$	$[M-H]^-$	3.619	3.623	536.2324	517.1914	0.917
Adducts/Neutral losses	372	372	472	177	pos_3649	neg_1537	pol / adduct	$[M+Na]^+$	$[M-H]^-$	3.676	3.666	545.0880	521.0924	0.761
MSDial	372	372	177	177	neg_1282	neg_1537	Pearson correlation	$[M-H]^-$	$[M-H]^-$	3.715	3.666	429.1755	521.0924	0.902
MSDial	372	372	177	177	neg_1495	neg_1537	Pearson correlation	$[M-H]^-$	$[M-H]^-$	3.712	3.666	505.0978	521.0924	0.949
Adducts/Neutral losses	372	372	457	177	pos_3540	neg_1537	pol / adduct	$[M+H]^+$	$[M-H]^-$	3.666	3.666	523.1078	521.0924	0.933
MSDial	372	372	177	177	neg_1282	neg_1565	Pearson correlation	$[M-H]^-$	$[M-H]^-$	3.715	3.763	429.1755	535.1082	0.954
Adducts/Neutral losses	372	372	479	177	pos_3700	neg_1565	pol / adduct	$[M+Na]^+$	$[M-H]^-$	3.768	3.763	559.1035	535.1082	0.697
Adducts/Neutral losses	372	372	177	177	neg_1687	neg_1587	neutral loss	CH_2O_2	CH_2O_2	3.680	3.681	589.0803	543.0743	0.962
Adducts/Neutral losses	372	372	177	177	neg_1662	neg_1587	neutral loss	CH_2O	CH_2O	3.726	3.681	573.0817	543.0743	0.756
Adducts/Neutral losses	372	372	457	177	pos_3540	neg_1587	pol / adduct	$[M+H]^+$	$[M+Na-2H]^-$	3.666	3.681	523.1078	543.0743	0.880
MSDial	372	372	177	177	neg_1537	neg_1587	Pearson correlation	$[M-H]^-$	$[M+Na-2H]^-$	3.666	3.681	521.0924	543.0743	0.946
MSDial	372	372	177	177	neg_1537	neg_1587	pol / adduct	$[M-H]^-$	$[M+Na-2H]^-$	3.666	3.681	521.0924	543.0743	1.000

Adducts/Neutral losses	372	372	472	177	pos_3649	neg_1587	pol / adduct	$[M+Na]^{+}$	$[M+Na-2H]^{-}$	3.676	3.681	545.0880	543.0743	0.792
MSDial	372	372	177	177	neg_1537	neg_1626	found in higher mz's MSMS	$[M-H]^{-}$	$[M+Cl]^{-}$	3.666	3.672	521.0924	557.0691	1.000
MSDial	372	372	177	177	neg_1537	neg_1626	pol / adduct	$[M-H]^{-}$	$[M+Cl]^{-}$	3.666	3.672	521.0924	557.0691	1.000
MSDial	372	372	177	177	neg_1587	neg_1626	Pearson correlation	$[M+Na-2H]^{-}$	$[M+Cl]^{-}$	3.681	3.672	543.0743	557.0691	0.941
Adducts/Neutral losses	372	372	472	177	pos_3649	neg_1626	pol / adduct	$[M+Na]^{+}$	$[M+Cl]^{-}$	3.676	3.672	545.0880	557.0691	0.900
Adducts/Neutral losses	372	372	457	177	pos_3540	neg_1626	pol / adduct	[M+H]+	$[M+Cl]^{-}$	3.666	3.672	523.1078	557.0691	0.948
MSDial	372	372	177	177	neg_1537	neg_1626	Pearson correlation	$[M-H]^{-}$	$[M+Cl]^{-}$	3.666	3.672	521.0924	557.0691	0.937
MSDial	372	372	177	177	neg_1565	neg_1656	pol / adduct	$[M-H]^{-}$	$[M+Cl]^{-}$	3.763	3.762	535.1082	571.0848	1.000
Adducts/Neutral losses	372	372	479	177	pos_3700	neg_1656	pol / adduct	$[M+Na]^{+}$	$[M+Cl]^{-}$	3.768	3.762	559.1035	571.0848	0.854
MSDial	372	372	177	177	neg_1565	neg_1656	Pearson correlation	$[M-H]^{-}$	$[M+Cl]^{-}$	3.763	3.762	535.1082	571.0848	0.916
MSDial	372	372	177	177	neg_1565	neg_1662	pol / adduct	$[M-H]^{-}$	$[M+K-2H]^{-}$	3.763	3.726	535.1082	573.0817	1.000
Adducts/Neutral losses	372	372	177	177	neg_1704	neg_1662	neutral loss	CH_2O	CH_2O	3.766	3.726	603.0956	573.0817	0.769
MSDial	372	372	177	177	neg_1537	neg_1682	Pearson correlation	$[M-H]^{-}$	$[M-H]^{-}$	3.666	3.675	521.0924	584.0884	0.933
MSDial	372	372	177	177	neg_1626	neg_1682	Pearson correlation	$[M+Cl]^{-}$	$[M-H]^{-}$	3.672	3.675	557.0691	584.0884	0.960
MSDial	372	372	177	177	neg_1587	neg_1682	Pearson correlation	$[M+Na-2H]^{-}$	$[M-H]^{-}$	3.681	3.675	543.0743	584.0884	0.949
MSDial	372	372	177	177	neg_1537	neg_1682	found in higher mz's MSMS	$[M-H]^{-}$	$[M-H]^{-}$	3.666	3.675	521.0924	584.0884	1.000
MSDial	372	372	177	177	neg_1587	neg_1687	Pearson correlation	$[M+Na-2H]^{-}$	$[M-H]^{-}$	3.681	3.680	543.0743	589.0803	0.962
Adducts/Neutral losses	372	372	177	177	neg_1760	neg_1687	neutral loss	CO_2	CO_2	3.673	3.680	633.0698	589.0803	0.882
MSDial	372	372	177	177	neg_1626	neg_1687	Pearson correlation	$[M+Cl]^{-}$	$[M-H]^{-}$	3.672	3.680	557.0691	589.0803	0.929
MSDial	372	372	177	177	neg_1682	neg_1687	Pearson correlation	$[M-H]^{-}$	$[M-H]^{-}$	3.675	3.680	584.0884	589.0803	0.906
MSDial	372	372	177	177	neg_1537	neg_1687	Pearson correlation	$[M-H]^{-}$	$[M-H]^{-}$	3.666	3.680	521.0924	589.0803	0.908
MSDial	372	372	177	177	neg_1587	neg_1687	found in higher mz's MSMS	$[M+Na-2H]^{-}$	$[M-H]^{-}$	3.681	3.680	543.0743	589.0803	1.000

MSDial	372	372	177	177	neg_1537	neg_1707	Pearson correlation	[M-H]$^-$	[M+Na-2H]$^-$	3.666	3.680	521.0924	605.0444	0.912
MSDial	372	372	177	177	neg_1587	neg_1707	Pearson correlation	[M+Na-2H]$^-$	[M+Na-2H]$^-$	3.681	3.680	543.0743	605.0444	0.964
MSDial	372	372	177	177	neg_1682	neg_1707	Pearson correlation	[M-H]$^-$	[M+Na-2H]$^-$	3.675	3.680	584.0884	605.0444	0.917
MSDial	372	372	177	177	neg_1626	neg_1707	Pearson correlation	[M+Cl]-	[M+Na-2H]$^-$	3.672	3.680	557.0691	605.0444	0.930
MSDial	372	372	177	177	neg_1687	neg_1707	Pearson correlation	[M-H]$^-$	[M+Na-2H]$^-$	3.680	3.680	589.0803	605.0444	0.945
Adducts/Neutral losses	372	372	177	177	neg_1732	neg_1707	neutral loss	CH_2	CH_2	3.663	3.680	619.0588	605.0444	0.832
MSDial	372	372	177	177	neg_1682	neg_1717	Pearson correlation	[M-H]$^-$	[M+Na-2H]$^-$	3.675	3.665	584.0884	611.0872	0.936
MSDial	372	372	177	177	neg_1526	neg_1717	Pearson correlation	[M-H]$^-$	[M+Na-2H]$^-$	3.623	3.665	517.1914	611.0872	0.908
MSDial	372	372	177	177	neg_1537	neg_1717	Pearson correlation	[M-H]$^-$	[M+Na-2H]$^-$	3.666	3.665	521.0924	611.0872	0.911
MSDial	372	372	177	177	neg_1626	neg_1717	Pearson correlation	[M+Cl]$^-$	[M+Na-2H]$^-$	3.672	3.665	557.0691	611.0872	0.935
MSDial	372	372	177	177	neg_1537	neg_1717	found in higher mz's MSMS	[M-H]$^-$	[M+Na-2H]$^-$	3.666	3.665	521.0924	611.0872	1.000
MSDial	372	372	177	177	neg_1687	neg_1717	pol / adduct	[M-H]$^-$	[M+Na-2H]$^-$	3.680	3.665	589.0803	611.0872	1.000
MSDial	372	372	177	177	neg_1717	neg_1732	Pearson correlation	[M+Na-2H]$^-$	[M+Cl]$^-$	3.665	3.663	611.0872	619.0588	0.906
Adducts/Neutral losses	372	372	177	177	neg_1760	neg_1732	neutral loss	CH_2	CH_2	3.673	3.663	633.0698	619.0588	0.889
MSDial	372	372	177	177	neg_1707	neg_1732	pol / adduct	[M+Na-2H]$^-$	[M+Cl]$^-$	3.680	3.663	605.0444	619.0588	1.000
MSDial	372	372	177	177	neg_1537	neg_1732	found in higher mz's MSMS	[M-H]$^-$	[M+Cl]$^-$	3.666	3.663	521.0924	619.0588	1.000
MSDial	372	372	177	177	neg_1565	neg_1741	Pearson correlation	[M-H]$^-$	[M+Na-2H]$^-$	3.763	3.763	535.1082	625.1033	0.907
MSDial	372	372	177	177	neg_1704	neg_1741	pol / adduct	[M-H]$^-$	[M+Na-2H]$^-$	3.766	3.763	603.0956	625.1033	1.000
MSDial	372	372	177	177	neg_1656	neg_1741	Pearson correlation	[M+Cl]$^-$	[M+Na-2H]$^-$	3.762	3.763	571.0848	625.1033	0.960
MSDial	372	372	177	177	neg_1587	neg_1760	found in higher mz's MSMS	[M+Na-2H]$^-$	[M-H]$^-$	3.681	3.673	543.0743	633.0698	1.000
MSDial	372	372	177	177	neg_1626	neg_1760	Pearson correlation	[M+Cl]-	[M-H]$^-$	3.672	3.673	557.0691	633.0698	0.909
MSDial	372	372	177	177	neg_1670	neg_1760	Pearson correlation	[M-H]$^-$	[M-H]$^-$	3.693	3.673	579.0517	633.0698	0.902
MSDial	372	372	177	177	neg_1537	neg_1943	found in higher mz's MSMS	[M-H]$^-$	[M-H]$^-$	3.666	3.663	521.0924	859.1560	1.000
MSDial	372	372	177	177	neg_1717	neg_1943	Pearson correlation	[M+Na-2H]$^-$	[M-H]$^-$	3.665	3.663	611.0872	859.1560	0.906

Adducts/Neutral losses	372	372	365	177	pos_2162	neg_948	pol / adduct	$[M+H]^+$	$[M-H]^-$	3.674	3.677	344.1479	342.1338	0.976
MSDial	372	372	177	177	neg_1943	neg_948	similar chromatogram in higher mz	$[M-H]^-$	$[M-H]^-$	3.663	3.677	859.1560	342.1338	1.000
MSDial	372	372	177	177	neg_1587	neg_967	found in higher mz's MSMS	$[M+Na-2H]^-$	$[M-H]^-$	3.681	3.667	543.0743	345.0583	1.000
MSDial	372	372	177	177	neg_1626	neg_967	Pearson correlation	$[M+Cl]^-$	$[M-H]^-$	3.672	3.667	557.0691	345.0583	0.938
MSDial	372	372	177	177	neg_1626	neg_967	found in higher mz's MSMS	$[M+Cl]^-$	$[M-H]^-$	3.672	3.667	557.0691	345.0583	1.000
MSDial	372	372	177	177	neg_1707	neg_967	found in higher mz's MSMS	$[M+Na-2H]^-$	$[M-H]^-$	3.680	3.667	605.0444	345.0583	1.000
MSDial	372	372	177	177	neg_1537	neg_967	found in higher mz's MSMS	$[M-H]^-$	$[M-H]^-$	3.666	3.667	521.0924	345.0583	1.000
MSDial	372	372	177	177	neg_1717	neg_967	Pearson correlation	$[M+Na-2H]^-$	$[M-H]^-$	3.665	3.667	611.0872	345.0583	0.948
MSDial	372	372	177	177	neg_1760	neg_967	found in higher mz's MSMS	$[M-H]^-$	$[M-H]^-$	3.673	3.667	633.0698	345.0583	1.000
MSDial	372	372	177	177	neg_1682	neg_967	found in higher mz's MSMS	$[M-H]^-$	$[M-H]^-$	3.675	3.667	584.0884	345.0583	1.000
Adducts/Neutral losses	372	372	457	177	pos_2179	neg_967	pol / adduct	$[M+H]^+$	$[M-H]^-$	3.658	3.667	347.0718	345.0583	0.920
MSDial	372	372	177	177	neg_1495	neg_967	Pearson correlation	$[M-H]^-$	$[M-H]^-$	3.712	3.667	505.0978	345.0583	0.923
MSDial	372	372	177	177	neg_1943	neg_967	found in higher mz's MSMS	$[M-H]^-$	$[M-H]^-$	3.663	3.667	859.1560	345.0583	1.000
MSDial	372	372	177	177	neg_948	neg_967	similar chromatogram in higher mz	$[M-H]^-$	$[M-H]^-$	3.677	3.667	342.1338	345.0583	1.000
MSDial	372	372	177	177	neg_1717	neg_967	found in higher mz's MSMS	$[M+Na-2H]^-$	$[M-H]^-$	3.665	3.667	611.0872	345.0583	1.000
MSDial	372	372	177	177	neg_1526	neg_967	Pearson correlation	[M-H]-	$[M-H]^-$	3.623	3.667	517.1914	345.0583	0.917
MSDial	372	372	177	177	neg_1587	neg_967	Pearson correlation	$[M+Na-2H]^-$	$[M-H]^-$	3.681	3.667	543.0743	345.0583	0.917
MSDial	372	372	177	177	neg_1687	neg_967	found in higher mz's MSMS	$[M-H]^-$	$[M-H]^-$	3.680	3.667	589.0803	345.0583	1.000
MSDial	372	372	177	177	neg_1682	neg_967	Pearson correlation	$[M-H]^-$	$[M-H]^-$	3.675	3.667	584.0884	345.0583	0.941
MSDial	372	372	177	177	neg_1537	neg_967	Pearson correlation	$[M-H]^-$	$[M-H]^-$	3.666	3.667	521.0924	345.0583	0.979
Adducts/Neutral losses	372	372	457	457	pos_3540	pos_3462	neutral loss	O	O	3.666	3.713	523.1078	507.1125	0.934
MSDial	372	372	457	457	pos_3462	pos_3540	Pearson correlation	$[M+H]^+$	$[M+H]^+$	3.713	3.666	507.1125	523.1078	0.934
MSDial	372	372	457	457	pos_2179	pos_3540	Pearson correlation	$[M+H]^+$	$[M+H]^+$	3.658	3.666	347.0718	523.1078	0.944

Adducts/Neutral losses	378	378	466	96	pos_3608	neg_1676	pol / adduct	$[M+H]^+$	$[M+FA-H]^-$	7.746	7.732	537.5332	581.5245	0.317
Adducts/Neutral losses	382	382	471	92	pos_3647	neg_1624	pol / adduct	$[M+Na]^+$	$[M+Cl]^-$	5.640	5.644	544.3372	556.3165	0.955
Adducts/Neutral losses	382	382	471	92	pos_3538	neg_1624	pol / adduct	$[M+H]^+$	$[M+Cl]^-$	5.638	5.644	522.3540	556.3165	0.961
MSDial	382	382	92	92	neg_1624	neg_1648	pol / adduct	$[M+Cl]^-$	$[M+FA-H]^-$	5.644	5.642	556.3165	566.3452	1.000
MSDial	382	382	92	92	neg_1624	neg_1648	Pearson correlation	$[M+Cl]^-$	$[M+FA-H]^-$	5.644	5.642	556.3165	566.3452	0.982
Adducts/Neutral losses	382	382	471	92	pos_3647	neg_1648	pol / adduct	$[M+Na]^+$	$[M+FA-H]^-$	5.640	5.642	544.3372	566.3452	0.977
Adducts/Neutral losses	382	382	471	92	pos_3538	neg_1648	pol / adduct	$[M+H]^+$	$[M+FA-H]^-$	5.638	5.642	522.3540	566.3452	0.990
MSDial	382	382	92	92	neg_1624	neg_1763	Pearson correlation	[M+Cl]-	$[M-H]^-$	5.644	5.639	556.3165	634.3329	0.980
MSDial	382	382	92	92	neg_1648	neg_1763	Pearson correlation	$[M+FA-H]^-$	$[M-H]^-$	5.642	5.639	566.3452	634.3329	0.983
MSDial	382	382	471	471	pos_3538	pos_3647	Pearson correlation	$[M+H]^+$	$[M+Na]^+$	5.638	5.640	522.3540	544.3372	0.985
MSDial	382	382	471	471	pos_3538	pos_3647	pol / adduct	$[M+H]^+$	$[M+Na]^+$	5.638	5.640	522.3540	544.3372	1.000
MSDial	386	386	104	104	neg_1586	neg_1609	Pearson correlation	$[M-H]^-$	$[M-H_2O-H]^-$	5.354	5.388	542.2490	552.2853	0.921
Adducts/Neutral losses	386	386	476	104	pos_3513	neg_1609	pol / adduct	$[M+H]^+$	[M+Cl]-	5.386	5.388	518.3240	552.2853	0.959
Adducts/Neutral losses	386	386	476	102	pos_3676	neg_1631	pol / adduct	$[M+H]^+$	$[M+FA-H]^-$	5.366	5.371	553.2750	559.3113	0.843
Adducts/Neutral losses	386	386	102	102	neg_1706	neg_1631	neutral loss	CO_2	CO_2	5.372	5.371	603.3011	559.3113	0.976
MSDial	386	386	102	102	neg_1631	neg_1706	Pearson correlation	$[M+FA-H]^-$	$[M+Cl]^-$	5.371	5.372	559.3113	603.3011	0.976
MSDial	386	386	104	104	neg_1609	neg_1710	Pearson correlation	$[M-H_2O-H]^-$	$[M+Cl]^-$	5.388	5.391	552.2853	606.3036	0.935
MSDial	386	386	104	104	neg_1710	neg_1735	Pearson correlation	$[M+Cl]^-$	$[M-H]^-$	5.391	5.393	606.3036	620.2719	0.958
MSDial	386	386	104	104	neg_1609	neg_1735	Pearson correlation	$[M-H_2O-H]^-$	$[M-H]^-$	5.388	5.393	552.2853	620.2719	0.910
MSDial	386	386	104	104	neg_1710	neg_1755	Pearson correlation	$[M+Cl]^-$	$[M-H]^-$	5.391	5.390	606.3036	630.3012	0.947
MSDial	386	386	104	104	neg_1667	neg_1755	similar chromatogram in higher mz	$[M+Cl]^-$	$[M-H]^-$	5.399	5.390	576.3295	630.3012	1.000

MSDial	386	386	104	104	neg_1609	neg_1755	Pearson correlation	$[M-H_2O-H]^-$	$[M-H]^-$	5.388	5.390	552.2853	630.3012	0.985
MSDial	386	386	104	104	neg_1735	neg_1755	Pearson correlation	$[M-H]^-$	$[M-H]^-$	5.393	5.390	620.2719	630.3012	0.924
MSDial	386	386	476	476	pos_3513	pos_3676	found in higher mz's MSMS	$[M+H]^+$	$[M+K]^+$	5.386	5.366	518.3240	553.2750	1.000
MSDial	391	391	484	484	pos_2026	pos_3785	similar chromatogram in higher mz	$[M+H]^+$	$[M+H]^+$	5.417	5.431	332.3149	588.3663	1.000
MSDial	391	391	484	484	pos_2026	pos_921	Pearson correlation	$[M+H]^+$	$[M+H]^+$	5.417	5.462	332.3149	228.2316	0.939
Adducts/Neutral losses	395	395	490	194	pos_3863	neg_1719	pol / adduct	$[M+H]^+$	$[M-H]^-$	0.920	0.928	613.1561	611.1430	0.979
MSDial	395	395	194	194	neg_1719	neg_306	found in higher mz's MSMS	$[M-H]^-$	$[M-H]^-$	0.928	0.921	611.1430	191.0192	1.000
Adducts/Neutral losses	395	395	424	194	pos_304	neg_306	pol / adduct	$[M+H]^+$	$[M+FA-H]^-$	0.914	0.921	147.0285	191.0192	0.990
MSDial	395	395	490	490	pos_1734	pos_3863	Pearson correlation	$[M+H]^+$	$[2M+H]^+$	0.942	0.920	307.0830	613.1561	0.974
Adducts/Neutral losses	398	398	493	99	pos_3884	neg_1694	pol / adduct	$[M+Na]^+$	$[M-H]^-$	5.978	5.980	615.2570	591.2600	0.928
Adducts/Neutral losses	399	399	494	283	pos_3885	neg_1724	pol / adduct	$[M+H]^+$	$[M-H]^-$	4.532	4.531	615.2780	613.2656	0.998
MSDial	399	399	283	283	neg_1724	neg_453	similar chromatogram in higher mz	$[M-H]^-$	$[M-H]^-$	4.531	4.526	613.2656	229.1439	1.000
MSDial	399	399	283	283	neg_1163	neg_898	Pearson correlation	$[M-H]^-$	$[M-H]^-$	4.503	4.504	392.2283	329.2327	0.937
MSDial	399	399	283	283	neg_453	neg_898	found in higher mz's MSMS	$[M-H]^-$	$[M-H]^-$	4.526	4.504	229.1439	329.2327	1.000
Adducts/Neutral losses	401	401	496	98	pos_3798	neg_1688	pol / adduct	$[M+H]^+$	$[M-H]^-$	3.445	3.446	592.2017	590.1866	0.944
Adducts/Neutral losses	401	401	496	496	pos_3903	pos_3798	neutral loss	CH_2O	CH_2O	3.443	3.445	622.2118	592.2017	0.916
MSDial	401	401	496	496	pos_3798	pos_3903	Pearson correlation	$[M+H]^+$	$[M+H]^+$	3.445	3.443	592.2017	622.2118	0.916
Adducts/Neutral losses	402	402	497	103	pos_3911	neg_1709	pol / adduct	$[M+NH_4]^+$	$[M-H]^-$	3.320	3.322	624.2263	605.1867	0.858
MSDial	405	405	501	501	pos_3901	pos_3975	Pearson correlation	$[M+H]^+$	$[M+Na]^+$	6.025	6.024	621.2708	643.2510	0.963
MSDial	405	405	501	501	pos_3901	pos_3975	pol / adduct	$[M+H]^+$	$[M+Na]^+$	6.025	6.024	621.2708	643.2510	1.000
Adducts/Neutral losses	408	408	504	120	pos_3995	neg_1778	pol / adduct	$[M+H]^+$	$[M-H]^-$	4.047	4.048	649.2855	647.2712	0.984

Adducts/Neutral losses	409	409	505	125	pos_4033	neg_1791	pol / adduct	$[M+H]^+$	$[M-H]^-$	4.097	4.087	659.2694	657.2554	0.948
Adducts/Neutral losses	410	410	436	58	pos_3232	neg_1437	pol / adduct	$[M+Na]^+$	$[M+Cl]^-$	4.231	4.235	471.2182	483.1992	0.905
Adducts/Neutral losses	410	410	436	58	pos_3201	neg_1437	pol / adduct	$[M+NH_4]^+$	$[M+Cl]^-$	4.238	4.235	466.2634	483.1992	0.910
Adducts/Neutral losses	410	410	436	58	pos_3232	neg_1462	pol / adduct	$[M+Na]^+$	$[M+FA-H]^-$	4.231	4.236	471.2182	493.2280	0.902
Adducts/Neutral losses	410	410	436	58	pos_3201	neg_1462	pol / adduct	$[[M+NH_4]^+$	$[M+FA-H]^-$	4.238	4.236	466.2634	493.2280	0.919
MSDial	410	410	58	58	neg_1437	neg_1462	pol / adduct	$[M+Cl]^-$	$[M+FA-H]^-$	4.235	4.236	483.1992	493.2280	1.000
MSDial	410	410	58	58	neg_1437	neg_1462	Pearson correlation	$[M+Cl]^-$	$[M+FA-H]^-$	4.235	4.236	483.1992	493.2280	0.931
Adducts/Neutral losses	410	410	506	118	pos_4048	neg_1771	pol / adduct	$[M+H]^+$	$[M-H_2O-H]^-$	4.231	4.259	663.3016	643.2763	0.683
MSDial	410	410	58	58	neg_1462	neg_1797	similar chromatogram in higher mz	$[M+FA-H]^-$	$[M-H]^-$	4.236	4.229	493.2280	661.2869	1.000
Adducts/Neutral losses	410	410	506	58	pos_4048	neg_1797	pol / adduct	$[M+H]^+$	$[M-H]^-$	4.231	4.229	663.3016	661.2869	0.996
MSDial	410	410	436	436	pos_3201	pos_3232	pol / adduct	$[M+NH_4]^+$	$[M+Na]^+$	4.238	4.231	466.2634	471.2182	1.000
Adducts/Neutral losses	412	412	509	150	pos_4064	neg_1860	pol / adduct	$[M+H]^+$	$[M+FA-H]^-$	2.941	2.935	668.2553	712.2446	0.975
MSDial	413	413	510	510	pos_2509	pos_4068	similar chromatogram in higher mz	$[M+H]^+$	[M+H]+	2.956	2.955	377.1443	671.1808	1.000
Adducts/Neutral losses	415	415	512	132	pos_4088	neg_1813	pol / adduct	$[M+H]^+$	$[M-H]^-$	3.663	3.669	677.2790	675.2658	0.944
Adducts/Neutral losses	416	416	517	134	pos_4096	neg_1815	pol / adduct	$[M+H]^+$	$[M-H]^-$	4.074	4.076	679.2977	677.2809	0.967
Adducts/Neutral losses	417	417	518	135	pos_4103	neg_1822	pol / adduct	$[M+H]^+$	$[M-H]^-$	4.293	4.295	683.1598	681.1455	0.862
Adducts/Neutral losses	418	418	519	137	pos_4110	neg_1824	pol / adduct	$[M+H]^+$	$[M-H]^-$	3.176	3.171	685.1582	683.1447	0.855
Adducts/Neutral losses	420	420	521	130	pos_4119	neg_1803	pol / adduct	$[M+Na]^+$	$[M-H]^-$	4.327	4.359	691.3661	667.3684	0.742

Adducts/Neutral losses	421	421	522	159	pos_4067	neg_1848	pol / adduct	[M+H]$^+$	[M+Cl]$^-$	3.479	3.477	671.1805	705.1429	0.935
Adducts/Neutral losses	421	421	522	159	pos_4121	neg_1848	pol / adduct	[M+Na]$^+$	[M+Cl]$^-$	3.480	3.477	693.1625	705.1429	0.884
MSDial	421	421	159	159	neg_1848	neg_1892	Pearson correlation	[M+Cl]$^-$	[M+Na-2H]$^-$	3.477	3.476	705.1429	759.1606	0.938
MSDial	421	421	522	522	pos_4067	pos_4121	pol / adduct	[M+H]$^+$	[M+Na]$^+$	3.479	3.480	671.1805	693.1625	1.000
Adducts/Neutral losses	422	422	529	155	pos_4153	neg_1871	pol / adduct	[M+Na]$^+$	[M+FA-H]$^-$	5.392	5.396	701.3704	723.3797	0.964
Adducts/Neutral losses	422	422	529	155	pos_4136	neg_1871	pol / adduct	[M+NH$_4$]$^+$	[M+FA-H]$^-$	5.392	5.396	696.4151	723.3797	0.984
MSDial	422	422	529	529	pos_4136	pos_4153	Pearson correlation	[M+H]$^+$	[M+H]$^+$	5.392	5.392	696.4151	701.3704	0.968
Adducts/Neutral losses	423	423	524	141	pos_4138	neg_1838	pol / adduct	[M+H]$^+$	[M-H]$^-$	4.409	4.399	697.1394	695.1240	0.948
Adducts/Neutral losses	424	424	525	209	pos_4141	neg_1819	pol / adduct	[M+NH$_4$]$^+$	[M-H]$^-$	3.458	3.456	698.2835	679.2440	0.932
Adducts/Neutral losses	424	424	209	209	neg_1932	neg_1915	neutral loss	CH_2O	CH_2O	3.460	3.431	831.1978	801.1872	0.938
Adducts/Neutral losses	424	424	540	209	pos_4299	neg_1915	pol / adduct	[M+H]$^+$	[M-H]$^-$	3.430	3.431	803.2029	801.1872	0.831
Adducts/Neutral losses	424	424	551	209	pos_4349	neg_1932	pol / adduct	[M+Na]$^+$	[M-H]$^-$	3.465	3.460	855.1959	831.1978	0.908
Adducts/Neutral losses	424	424	551	209	pos_4332	neg_1932	pol / adduct	[M+H]$^+$	[M-H]$^-$	3.463	3.460	833.2134	831.1978	0.941
MSDial	424	424	209	209	neg_1915	neg_1932	Pearson correlation	[M-H]$^-$	[M-H]$^-$	3.431	3.460	801.1872	831.1978	0.938
Adducts/Neutral losses	424	424	551	209	pos_4349	neg_1944	pol / adduct	[M+Na]$^+$	[M+Cl]$^-$	3.465	3.462	855.1959	867.1741	0.811
Adducts/Neutral losses	424	424	551	209	pos_4332	neg_1944	pol / adduct	[M+H]$^+$	[M+Cl]$^-$	3.463	3.462	833.2134	867.1741	0.844
MSDial	424	424	209	209	neg_1932	neg_1944	pol / adduct	[M-H]$^-$	[M+Cl]$^-$	3.460	3.462	831.1978	867.1741	1.000
MSDial	424	424	209	209	neg_1932	neg_1944	Pearson correlation	[M-H]$^-$	[M+Cl]$^-$	3.460	3.462	831.1978	867.1741	0.939
MSDial	424	424	209	209	neg_1819	neg_446	similar chromatogram in higher mz	[M+K-2H]$^-$	[M-H]$^-$	3.456	3.459	679.2440	227.0920	1.000

MSDial	424	424	209	209	neg_1932	neg_446	similar chromatogram in higher mz	$[M-H]^{-}$	$[M-H]^{-}$	3.460	3.459	831.1978	227.0920	1.000
MSDial	424	424	209	209	neg_446	neg_914	similar chromatogram in higher mz	$[M-H]^{-}$	$[M-H]^{-}$	3.459	3.460	227.0920	333.1450	1.000
Adducts/Neutral losses	424	424	357	209	pos_2065	neg_914	pol / adduct	$[M+H]^{+}$	$[M-H]^{-}$	3.469	3.460	335.1585	333.1450	0.868
MSDial	424	424	551	551	pos_4332	pos_4349	Pearson correlation	$[M+H]^{+}$	$[M+H]^{+}$	3.463	3.465	833.2134	855.1959	0.952
MSDial	424	424	551	551	pos_2984	pos_4349	Pearson correlation	$[M+NH_4]^{2+}$	$[M+H]^{+}$	3.464	3.465	436.0828	855.1959	0.917
Adducts/Neutral losses	426	426	527	143	pos_4146	neg_1841	pol / adduct	$[M+H]^{+}$	$[M-H]^{-}$	4.138	4.137	699.1550	697.1391	0.951
Adducts/Neutral losses	427	427	528	152	pos_4095	neg_1863	pol / adduct	$[M+H]^{+}$	$[M+Cl]^{-}$	3.695	3.698	679.2974	713.2582	0.980
Adducts/Neutral losses	427	427	528	152	pos_4151	neg_1863	pol / adduct	$[M+Na]^{+}$	$[M+Cl]^{-}$	3.695	3.698	701.2773	713.2582	0.985
MSDial	427	427	528	528	pos_4095	pos_4151	pol / adduct	$[M+H]^{+}$	$[M+Na]^{+}$	3.695	3.695	679.2974	701.2773	1.000
MSDial	427	427	528	528	pos_4095	pos_4151	Pearson correlation	$[M+H]^{+}$	$[M+Na]^{+}$	3.695	3.695	679.2974	701.2773	0.991
Adducts/Neutral losses	432	432	535	164	pos_4264	neg_1900	pol / adduct	$[M+H]^{+}$	$[M-H]^{-}$	3.234	3.237	773.2125	771.1976	0.913
Adducts/Neutral losses	433	433	536	165	pos_4281	neg_1904	pol / adduct	$[M+H]^{+}$	$[M-H]^{-}$	3.334	3.340	787.1905	785.1768	0.754
Adducts/Neutral losses	434	434	537	173	pos_4282	neg_1930	pol / adduct	$[M+H]^{+}$	$[M+Cl]^{-}$	3.096	3.100	789.2046	823.1691	0.757
Adducts/Neutral losses	434	434	543	173	pos_4309	neg_1930	pol / adduct	$[M+Na]^{+}$	$[M+Cl]^{-}$	3.103	3.100	811.1863	823.1691	0.797
MSDial	436	436	539	539	pos_3947	pos_4286	Pearson correlation	$[M+H]^{+}$	$[M+2H]^{2+}$	3.016	3.017	633.7146	791.6435	0.925
Adducts/Neutral losses	439	439	547	171	pos_4318	neg_1923	pol / adduct	$[M+H]^{+}$	$[M-H]^{-}$	2.966	2.966	819.2163	817.2028	0.850
Adducts/Neutral losses	441	441	544	180	pos_4328	neg_1941	pol / adduct	$[M+NH_4]^{+}$	$[M+FA-H]^{-}$	4.436	4.414	828.4330	855.4000	0.764
Adducts/Neutral losses	441	441	544	180	pos_4310	neg_1941	pol / adduct	$[M+H]^{+}$	$[M+FA-H]^{-}$	4.436	4.414	811.4109	855.4000	0.684
Adducts/Neutral losses	441	441	544	544	pos_4328	pos_4310	neutral loss	H_3N	H_3N	4.436	4.436	828.4330	811.4109	0.807

MSDial	441	441	544	544	pos_3509	pos_4310	Pearson correlation	$[M+CH_3OH+H]^+$	$[M+H]^+$	4.437	4.436	517.3512	811.4109	0.911
MSDial	441	441	544	544	pos_4310	pos_4328	pol / adduct	$[M+H]^+$	$[M+NH_4]^+$	4.436	4.436	811.4109	828.4330	1.000
MSDial	442	442	549	549	pos_4305	pos_4331	pol / adduct	$[M+H]^+$	$[M+Na]^+$	4.060	4.059	807.3427	829.3270	1.000
MSDial	442	442	549	549	pos_4305	pos_4331	Pearson correlation	$[M+H]^+$	$[M+Na]^+$	4.060	4.059	807.3427	829.3270	0.942
Adducts/Neutral losses	444	444	425	177	pos_3078	neg_1334	pol / adduct	$[M+H]^+$	$[M-H]^-$	3.639	3.621	447.1281	445.1130	0.906
Adducts/Neutral losses	444	444	546	177	pos_4314	neg_1922	pol / adduct	$[M+H]^+$	$[M-H]^-$	3.613	3.608	817.2178	815.2022	0.840
Adducts/Neutral losses	444	444	177	177	neg_1937	neg_1922	neutral loss	CH_2O	CH_2O	3.629	3.608	845.2125	815.2022	0.922
Adducts/Neutral losses	444	444	552	177	pos_4341	neg_1937	pol / adduct	$[M+H]^+$	$[M-H]^-$	3.631	3.629	847.2287	845.2125	0.871
MSDial	444	444	177	177	neg_1334	neg_1937	similar chromatogram in higher mz	$[M-H]^-$	$[M-H]^-$	3.621	3.629	445.1130	845.2125	1.000
MSDial	444	444	177	177	neg_1922	neg_1937	Pearson correlation	$[M-H]^-$	$[M-H]^-$	3.608	3.629	815.2022	845.2125	0.922
Adducts/Neutral losses	444	444	552	177	pos_4355	neg_1937	pol / adduct	$[M+Na]^+$	$[M-H]^-$	3.631	3.629	869.2065	845.2125	0.873
MSDial	444	372	177	177	neg_1937	neg_967	found in higher mz's MSMS	$[M-H]^-$	$[M-H]^-$	3.629	3.667	845.2125	345.0583	1.000
MSDial	444	444	552	552	pos_4341	pos_4355	pol / adduct	$[M+H]^+$	$[M+Na]^+$	3.631	3.631	847.2287	869.2065	1.000
MSDial	444	444	552	552	pos_4341	pos_4355	Pearson correlation	$[M+H]^+$	$[M+Na]^+$	3.631	3.631	847.2287	869.2065	0.938
MSDial	445	445	428	428	pos_311	pos_438	Pearson correlation	$[M+H]^+$	$[M+H]^+$	11.721	11.696	149.0120	165.0607	0.986
MSDial	445	445	428	428	pos_311	pos_656	Pearson correlation	$[M+H]^+$	$[M+CH_3OH+H]^+$	11.721	11.727	149.0120	195.0174	0.985
MSDial	445	445	428	428	pos_438	pos_656	Pearson correlation	$[M+H]^+$	$[M+CH_3OH+H]^+$	11.696	11.727	165.0607	195.0174	0.977
Adducts/Neutral losses	446	446	553	247	pos_444	neg_216	pol / adduct	$[M+H]^+$	$[M-H]^-$	1.971	1.977	166.0857	164.0713	0.961
Adducts/Neutral losses	446	446	553	247	pos_721	neg_216	pol / adduct	[M+K]+	$[M-H]^-$	1.962	1.977	204.0415	164.0713	0.736
Adducts/Neutral losses	446	446	553	247	pos_598	neg_216	pol / adduct	$[M+Na]^+$	$[M-H]^-$	1.973	1.977	188.0677	164.0713	0.804
MSDial	446	446	247	247	neg_216	neg_718	Pearson correlation	$[M-H]^-$	$[M+Na-2H]^-$	1.977	1.974	164.0713	294.0290	0.943

MSDial	446	446	553	553	pos_2522	pos_444	Pearson correlation	$[M+H]^+$	$[M+H]^+$	1.973	1.971	379.1670	166.0857	0.902
MSDial	446	446	553	553	pos_2522	pos_444	found in higher mz's MSMS	$[M+H]^+$	$[M+H]^+$	1.973	1.971	379.1670	166.0857	1.000
MSDial	446	446	553	553	pos_2522	pos_598	Pearson correlation	$[M+H]^+$	$[M+H]^+$	1.973	1.973	379.1670	188.0677	0.907
MSDial	446	446	553	553	pos_444	pos_721	pol / adduct	$[M+H]^+$	[M+K]+	1.971	1.962	166.0857	204.0415	1.000
MSDial	448	448	555	555	pos_2548	pos_457	Pearson correlation	$[M+H]^+$	$[M+H]^+$	1.793	1.793	381.1831	168.1014	0.911
MSDial	448	448	555	555	pos_2863	pos_457	found in higher mz's MSMS	$[M+CH_3OH+H]^+$	$[M+H]^+$	1.794	1.793	420.2225	168.1014	1.000
MSDial	448	448	555	555	pos_2548	pos_457	found in higher mz's MSMS	$[M+H]^+$	$[M+H]^+$	1.793	1.793	381.1831	168.1014	1.000
Adducts/Neutral losses	455	455	562	562	pos_1318	pos_1132	neutral loss	H_3N	H_3N	5.192	5.193	268.2268	251.2003	0.836
Adducts/Neutral losses	455	455	562	562	pos_1654	pos_1304	neutral loss	CH_5N	CH_5N	5.111	5.109	298.2129	267.1717	0.898
Adducts/Neutral losses	455	455	562	562	pos_2267	pos_1318	neutral loss	malonyl-H_2O	malonyl-H_2O	5.147	5.192	354.2265	268.2268	0.859
MSDial	455	455	562	562	pos_1132	pos_1370	Pearson correlation	$[M+H]^+$	$[M+H]^+$	5.193	5.192	251.2003	273.1826	0.974
MSDial	455	455	562	562	pos_1132	pos_1370	pol / adduct	$[M+H]^+$	$[M+H]^+$	5.193	5.192	251.2003	273.1826	1.000
MSDial	455	455	562	562	pos_1318	pos_1370	pol / adduct	$[M+NH_4]^+$	$[M+H]^+$	5.192	5.192	268.2268	273.1826	1.000
MSDial	455	455	562	562	pos_1213	pos_1432	Pearson correlation	[M+K]+	$[M+H]^+$	5.101	5.141	259.1894	279.1588	0.966
MSDial	455	455	562	562	pos_1304	pos_1545	Pearson correlation	[M+Na]+	$[M+Na]^+$	5.109	5.105	267.1717	289.1528	0.978
MSDial	455	455	562	562	pos_1304	pos_1545	pol / adduct	[M+Na]+	$[M+Na]^+$	5.109	5.105	267.1717	289.1528	1.000
MSDial	455	455	562	562	pos_1304	pos_1654	found in higher mz's MSMS	[M+Na]+	$[M+NH_4]^+$	5.109	5.111	267.1717	298.2129	1.000
MSDial	455	455	562	562	pos_1213	pos_1674	Pearson correlation	[M+K]+	$[M+Na]^+$	5.101	5.141	259.1894	301.1402	0.955
MSDial	455	455	562	562	pos_1432	pos_1674	Pearson correlation	$[M+H]^+$	$[M+Na]^+$	5.141	5.141	279.1588	301.1402	0.989
MSDial	455	455	562	562	pos_1432	pos_1674	pol / adduct	$[M+H]^+$	$[M+Na]^+$	5.141	5.141	279.1588	301.1402	1.000
MSDial	455	455	562	562	pos_1432	pos_1858	pol / adduct	$[M+H]^+$	[M+K]+	5.141	5.141	279.1588	317.1139	1.000
MSDial	455	467	562	576	pos_1132	pos_1991	Pearson correlation	$[M+H]^+$	$[M+CH_3OH+H]^+$	5.193	5.210	251.2003	329.2948	0.920
MSDial	455	455	562	562	pos_1674	pos_2139	found in higher mz's MSMS	$[M+Na]^+$	$[M+NH_4]^+$	5.141	5.129	301.1402	342.2633	1.000
MSDial	455	455	562	562	pos_1213	pos_2267	Pearson correlation	[M+K]+	$[M+CH_3OH+H]^+$	5.101	5.147	259.1894	354.2265	0.905

MSDial	455	455	562	562	pos_1674	pos_2267	Pearson correlation	$[M+Na]^+$	$[M+CH_3OH+H]^+$	5.141	5.147	301.1402	354.2265	0.938
MSDial	455	455	562	562	pos_1370	pos_2267	Pearson correlation	$[M+H]^+$	$[M+CH_3OH+H]^+$	5.192	5.147	273.1826	354.2265	0.955
MSDial	455	455	562	562	pos_1132	pos_2267	Pearson correlation	$[M+H]^+$	$[M+CH_3OH+H]^+$	5.193	5.147	251.2003	354.2265	0.939
MSDial	455	455	562	562	pos_1432	pos_2267	Pearson correlation	$[M+H]^+$	$[M+CH_3OH+H]^+$	5.141	5.147	279.1588	354.2265	0.946
Adducts/Neutral losses	455	455	562	562	pos_658	pos_315	neutral loss	C_2H_6O	C_2H_6O	5.143	5.141	195.0648	149.0232	0.953
MSDial	455	455	562	562	pos_2267	pos_315	Pearson correlation	$[M+CH_3OH+H]^+$	$[M+H]^+$	5.147	5.141	354.2265	149.0232	0.947
Adducts/Neutral losses	455	455	562	562	pos_424	pos_315	neutral loss	CH_2	CH_2	5.142	5.141	163.0387	149.0232	0.990
MSDial	455	455	562	562	pos_2267	pos_315	found in higher mz's MSMS	$[M+CH_3OH+H]^+$	$[M+H]^+$	5.147	5.141	354.2265	149.0232	1.000
Adducts/Neutral losses	455	455	562	562	pos_549	pos_315	neutral loss	CH_3OH	CH_3OH	5.141	5.141	181.0492	149.0232	0.990
MSDial	455	455	562	562	pos_1432	pos_315	found in higher mz's MSMS	$[M+H]^+$	$[M+H]^+$	5.141	5.141	279.1588	149.0232	1.000
MSDial	455	455	562	562	pos_1213	pos_315	Pearson correlation	$[M+K]^+$	$[M+H]^+$	5.101	5.141	259.1894	149.0232	0.955
MSDial	455	455	562	562	pos_1432	pos_315	Pearson correlation	$[M+H]^+$	$[M+H]^+$	5.141	5.141	279.1588	149.0232	0.992
MSDial	455	455	562	562	pos_1654	pos_315	found in higher mz's MSMS	$[M+NH_4]^+$	$[M+H]^+$	5.111	5.141	298.2129	149.0232	1.000
Adducts/Neutral losses	455	455	562	562	pos_549	pos_315	neutral loss	CH_4O	CH_4O	5.141	5.141	181.0492	149.0232	0.990
MSDial	455	455	562	562	pos_1674	pos_315	Pearson correlation	$[M+Na]^+$	$[M+H]^+$	5.141	5.141	301.1402	149.0232	0.980
MSDial	455	455	562	562	pos_1432	pos_3751	found in higher mz's MSMS	$[M+H]^+$	$[M+NH_4]^+$	5.141	5.142	279.1588	574.3350	1.000
MSDial	455	455	562	562	pos_315	pos_3751	found in higher mz's MSMS	$[M+H]^+$	$[M+NH_4]^+$	5.141	5.142	149.0232	574.3350	1.000
MSDial	455	455	562	562	pos_1213	pos_3763	Pearson correlation	$[M+K]^+$	$[M+Na]^+$	5.101	5.142	259.1894	579.2915	0.936
MSDial	455	455	562	562	pos_315	pos_3763	Pearson correlation	$[M+H]^+$	$[M+Na]^+$	5.141	5.142	149.0232	579.2915	0.947
MSDial	455	455	562	562	pos_1370	pos_3763	Pearson correlation	$[M+H]^+$	$[M+Na]^+$	5.192	5.142	273.1826	579.2915	0.945
MSDial	455	455	562	562	pos_1674	pos_3763	found in higher mz's MSMS	$[M+Na]^+$	$[M+Na]^+$	5.141	5.142	301.1402	579.2915	1.000
MSDial	455	455	562	562	pos_3751	pos_3763	pol / adduct	$[M+NH_4]^+$	$[M+Na]^+$	5.142	5.142	574.3350	579.2915	1.000
MSDial	455	455	562	562	pos_315	pos_3763	found in higher mz's MSMS	$[M+H]^+$	$[M+Na]^+$	5.141	5.142	149.0232	579.2915	1.000

MSDial	455	455	562	562	pos_1432	pos_3763	Pearson correlation	$[M+H]^+$	$[M+Na]^+$	5.141	5.142	279.1588	579.2915	0.962
MSDial	455	455	562	562	pos_2267	pos_3763	Pearson correlation	$[M+CH_3OH+H]^+$	$[M+Na]^+$	5.147	5.142	354.2265	579.2915	0.916
MSDial	455	455	562	562	pos_1674	pos_3763	Pearson correlation	$[M+Na]^+$	$[M+Na]^+$	5.141	5.142	301.1402	579.2915	0.983
MSDial	455	455	562	562	pos_2267	pos_424	Pearson correlation	$[M+CH_3OH+H]^+$	$[M+H]^+$	5.147	5.142	354.2265	163.0387	0.940
MSDial	455	455	562	562	pos_1370	pos_424	Pearson correlation	$[M+H]^+$	$[M+H]^+$	5.192	5.142	273.1826	163.0387	0.953
Adducts/Neutral losses	455	455	562	562	pos_658	pos_424	neutral loss	CH_3OH	CH_3OH	5.143	5.142	195.0648	163.0387	0.972
Adducts/Neutral losses	455	455	562	562	pos_658	pos_424	neutral loss	CH_4O	CH_4O	5.143	5.142	195.0648	163.0387	0.972
MSDial	455	455	562	562	pos_1213	pos_424	Pearson correlation	$[M+K]^+$	$[M+H]^+$	5.101	5.142	259.1894	163.0387	0.964
MSDial	455	455	562	562	pos_3763	pos_424	Pearson correlation	$[M+Na]^+$	$[M+H]^+$	5.142	5.142	579.2915	163.0387	0.968
MSDial	455	455	562	562	pos_1432	pos_424	Pearson correlation	$[M+H]^+$	$[M+H]^+$	5.141	5.142	279.1588	163.0387	0.996
Adducts/Neutral losses	455	455	562	562	pos_731	pos_424	neutral loss	C_3H_6	C_3H_6	5.143	5.142	205.0855	163.0387	0.996
MSDial	455	455	562	562	pos_1674	pos_424	Pearson correlation	$[M+Na]^+$	$[M+H]^+$	5.141	5.142	301.1402	163.0387	0.993
MSDial	455	455	562	562	pos_315	pos_424	Pearson correlation	$[M+H]^+$	$[M+H]^+$	5.141	5.142	149.0232	163.0387	0.990
Adducts/Neutral losses	455	455	562	562	pos_549	pos_424	neutral loss	H_2O	H_2O	5.141	5.142	181.0492	163.0387	0.997
MSDial	455	455	562	562	pos_315	pos_549	Pearson correlation	$[M+H]^+$	$[M+CH_3OH+H]^+$	5.141	5.141	149.0232	181.0492	0.990
MSDial	455	455	562	562	pos_1674	pos_549	Pearson correlation	$[M+Na]^+$	$[M+CH_3OH+H]^+$	5.141	5.141	301.1402	181.0492	0.992
MSDial	455	455	562	562	pos_3763	pos_549	Pearson correlation	$[M+Na]^+$	$[M+CH_3OH+H]^+$	5.142	5.141	579.2915	181.0492	0.970
Adducts/Neutral losses	455	455	562	562	pos_658	pos_549	neutral loss	CH_2	CH_2	5.143	5.141	195.0648	181.0492	0.973
MSDial	455	455	562	562	pos_1432	pos_549	Pearson correlation	$[M+H]^+$	$[M+CH_3OH+H]^+$	5.141	5.141	279.1588	181.0492	0.998
MSDial	455	455	562	562	pos_315	pos_549	pol / adduct	$[M+H]^+$	$[M+CH_3OH+H]^+$	5.141	5.141	149.0232	181.0492	1.000
MSDial	455	455	562	562	pos_424	pos_549	Pearson correlation	$[M+H]^+$	$[M+CH_3OH+H]^+$	5.142	5.141	163.0387	181.0492	0.997
MSDial	455	455	562	562	pos_2267	pos_549	Pearson correlation	$[M+CH_3OH+H]^+$	$[M+CH_3OH+H]^+$	5.147	5.141	354.2265	181.0492	0.952
MSDial	455	455	562	562	pos_315	pos_549	found in higher mz's MSMS	$[M+H]^+$	$[M+CH_3OH+H]^+$	5.141	5.141	149.0232	181.0492	1.000

MSDial	455	455	562	562	pos_1213	pos_549	Pearson correlation	[M+K]$^+$	[M+CH$_3$OH+H]$^+$	5.101	5.141	259.1894	181.0492	0.966
MSDial	455	455	562	562	pos_424	pos_549	found in higher mz's MSMS	[M+H]$^+$	[M+CH$_3$OH+H]$^+$	5.142	5.141	163.0387	181.0492	1.000
MSDial	455	455	562	562	pos_549	pos_658	Pearson correlation	[M+CH$_3$OH+H]$^+$	[M+CH$_3$OH+H]$^+$	5.141	5.143	181.0492	195.0648	0.973
MSDial	455	455	562	562	pos_424	pos_658	pol / adduct	[M+H]$^+$	[M+CH$_3$OH+H]$^+$	5.142	5.143	163.0387	195.0648	1.000
MSDial	455	455	562	562	pos_1132	pos_658	Pearson correlation	[M+H]$^+$	[M+CH$_3$OH+H]$^+$	5.193	5.143	251.2003	195.0648	0.954
MSDial	455	455	562	562	pos_2267	pos_658	Pearson correlation	[M+CH$_3$OH+H]$^+$	[M+CH$_3$OH+H]$^+$	5.147	5.143	354.2265	195.0648	0.932
MSDial	455	455	562	562	pos_1432	pos_658	Pearson correlation	[M+H]$^+$	[M+CH$_3$OH+H]$^+$	5.141	5.143	279.1588	195.0648	0.967
MSDial	455	455	562	562	pos_424	pos_658	Pearson correlation	[M+H]$^+$	[M+CH$_3$OH+H]$^+$	5.142	5.143	163.0387	195.0648	0.972
MSDial	455	455	562	562	pos_3763	pos_658	Pearson correlation	[M+Na]$^+$	[M+CH$_3$OH+H]$^+$	5.142	5.143	579.2915	195.0648	0.926
MSDial	455	455	562	562	pos_1674	pos_658	Pearson correlation	[M+Na]$^+$	[M+CH$_3$OH+H]$^+$	5.141	5.143	301.1402	195.0648	0.963
MSDial	455	455	562	562	pos_424	pos_658	found in higher mz's MSMS	[M+H]$^+$	[M+CH$_3$OH+H]$^+$	5.142	5.143	163.0387	195.0648	1.000
MSDial	455	455	562	562	pos_1370	pos_658	Pearson correlation	[M+H]$^+$	[M+CH$_3$OH+H]$^+$	5.192	5.143	273.1826	195.0648	0.946
MSDial	455	455	562	562	pos_315	pos_658	Pearson correlation	[M+H]$^+$	[M+CH$_3$OH+H]$^+$	5.141	5.143	149.0232	195.0648	0.953
MSDial	455	455	562	562	pos_1213	pos_658	Pearson correlation	[M+K]$^+$	[M+CH$_3$OH+H]$^+$	5.101	5.143	259.1894	195.0648	0.942
MSDial	455	455	562	562	pos_1370	pos_731	Pearson correlation	[M+H]$^+$	[M+H-H$_2$O]$^+$	5.192	5.143	273.1826	205.0855	0.943
MSDial	455	455	562	562	pos_1213	pos_731	Pearson correlation	[M+K]$^+$	[M+H-H$_2$O]$^+$	5.101	5.143	259.1894	205.0855	0.962
MSDial	455	455	562	562	pos_2267	pos_731	Pearson correlation	[M+CH$_3$OH+H]$^+$	[M+H-H$_2$O]$^+$	5.147	5.143	354.2265	205.0855	0.936
MSDial	455	455	562	562	pos_3763	pos_731	Pearson correlation	[M+Na]$^+$	[M+H-H$_2$O]$^+$	5.142	5.143	579.2915	205.0855	0.958
MSDial	455	455	562	562	pos_1654	pos_731	found in higher mz's MSMS	[M+NH$_4$]$^+$	[M+H-H$_2$O]$^+$	5.111	5.143	298.2129	205.0855	1.000
MSDial	455	455	562	562	pos_1674	pos_731	Pearson correlation	[M+Na]$^+$	[M+H-H$_2$O]$^+$	5.141	5.143	301.1402	205.0855	0.989
MSDial	455	455	562	562	pos_658	pos_731	Pearson correlation	[M+CH$_3$OH+H]$^+$	[M+H-H$_2$O]$^+$	5.143	5.143	195.0648	205.0855	0.966
MSDial	455	455	562	562	pos_315	pos_731	found in higher mz's MSMS	[M+H]$^+$	[M+H-H$_2$O]$^+$	5.141	5.143	149.0232	205.0855	1.000
MSDial	455	455	562	562	pos_1132	pos_731	Pearson correlation	[M+H]$^+$	[M+H-H$_2$O]$^+$	5.193	5.143	251.2003	205.0855	0.958
MSDial	455	455	562	562	pos_1432	pos_731	Pearson correlation	[M+H]$^+$	[M+H-H$_2$O]$^+$	5.141	5.143	279.1588	205.0855	0.996
MSDial	455	455	562	562	pos_3751	pos_731	found in higher mz's MSMS	[M+NH$_4$]$^+$	[M+H-H$_2$O]$^+$	5.142	5.143	574.3350	205.0855	1.000

MSDial	455	455	562	562	pos_1432	pos_731	found in higher mz's MSMS	$[M+H]^+$	$[M+H-H_2O]^+$	5.141	5.143	279.1588	205.0855	1.000
MSDial	455	455	562	562	pos_424	pos_731	Pearson correlation	$[M+H]^+$	$[M+H-H_2O]^+$	5.142	5.143	163.0387	205.0855	0.996
MSDial	455	455	562	562	pos_315	pos_731	Pearson correlation	$[M+H]^+$	$[M+H-H_2O]^+$	5.141	5.143	149.0232	205.0855	0.994
MSDial	455	455	562	562	pos_549	pos_731	Pearson correlation	$[M+CH_3OH+H]^+$	$[M+H-H_2O]^+$	5.141	5.143	181.0492	205.0855	0.993
Adducts/Neutral losses	456	456	563	189	pos_558	neg_257	pol / adduct	$[M+H]^+$	$[M-H]^-$	1.200	1.224	182.0808	180.0662	0.978
MSDial	456	456	189	189	neg_161	neg_257	similar chromatogram in higher mz	$[M-H]^-$	$[M-H]^-$	1.206	1.224	147.0296	180.0662	1.000
MSDial	456	456	189	189	neg_257	neg_533	found in higher mz's MSMS	$[M-H]^-$	$[M-H]^-$	1.224	1.238	180.0662	248.0534	1.000
MSDial	456	456	563	563	pos_2448	pos_558	found in higher mz's MSMS	$[M+H]^+$	$[M+H]^+$	1.160	1.200	371.1439	182.0808	1.000
MSDial	457	457	564	564	pos_218	pos_2559	found in higher mz's MSMS	$[M+H]^+$	$[M+H]^+$	1.478	1.466	132.1016	382.2068	1.000
MSDial	457	457	564	564	pos_218	pos_2640	found in higher mz's MSMS	$[M+H]^+$	$[M+NH_4]^+$	1.478	1.475	132.1016	391.1373	1.000
MSDial	457	457	564	564	pos_218	pos_56	found in higher mz's MSMS	$[M+H]^+$	$[M+H]^+$	1.478	1.467	132.1016	86.0962	1.000
MSDial	457	457	564	564	pos_218	pos_56	Pearson correlation	$[M+H]^+$	$[M+H]^+$	1.478	1.467	132.1016	86.0962	0.997
MSDial	460	460	566	566	pos_1494	pos_586	similar chromatogram in higher mz	$[M+H]^+$	$[M+H]^+$	4.711	4.707	285.0751	185.1532	1.000
MSDial	460	460	566	566	pos_586	pos_781	Pearson correlation	$[M+H]^+$	$[M+H]^+$	4.707	4.675	185.1532	211.1692	0.970
Adducts/Neutral losses	461	461	567	181	pos_204	neg_107	pol / adduct	$[M+H]^+$	$[M-H]^-$	1.041	1.029	130.0496	128.0350	0.981
MSDial	461	461	181	181	neg_107	neg_199	Pearson correlation	$[M-H]^-$	$[M-H]^-$	1.029	1.020	128.0350	161.0087	0.917
MSDial	461	461	181	181	neg_199	neg_21	Pearson correlation	$[M-H]^-$	$[M-H]^-$	1.020	1.019	161.0087	71.0137	0.982
MSDial	461	461	567	567	pos_204	pos_618	found in higher mz's MSMS	$[M+H]^+$	$[M+H]^+$	1.041	1.109	130.0496	190.0705	1.000
Adducts/Neutral losses	462	462	568	90	pos_619	neg_232	pol / adduct	$[M+NH_4]^+$	$[M-H]^-$	3.067	3.059	190.1071	171.0659	0.930
Adducts/Neutral losses	463	463	569	193	pos_620	neg_288	pol / adduct	$[M+H]^+$	$[M-H]^-$	1.874	1.880	190.1072	188.0923	0.818
MSDial	464	464	263	263	neg_1026	neg_1092	Pearson correlation	$[M-H]^-$	$[M-H]^-$	2.200	2.161	359.0974	375.0924	0.933
Adducts/Neutral losses	464	464	570	263	pos_2248	neg_1092	pol / adduct	$[M+Na]^+$	$[M+FA-H]^-$	2.161	2.161	353.0833	375.0924	0.755

Adducts/Neutral losses	464	464	570	263	pos_2195	neg_1092	pol / adduct	[M+NH4]+	[M+FA-H]-	2.161	2.161	348.1285	375.0924	0.838
Adducts/Neutral losses	464	464	570	263	pos_2570	neg_1115	pol / adduct	[M+H]+	[M-H]-	2.159	2.155	384.1149	382.0995	0.946
Adducts/Neutral losses	464	464	570	263	pos_634	neg_378	pol / adduct	[M+H-H2O]+	[M-H]-	2.163	2.164	192.0650	208.0610	0.904
MSDial	464	464	263	263	neg_1115	neg_378	Pearson correlation	[M-H]-	[M-H]-	2.155	2.164	382.0995	208.0610	0.975
Adducts/Neutral losses	464	464	570	263	pos_2195	neg_894	pol / adduct	[M+NH4]+	[M-H]-	2.161	2.162	348.1285	329.0873	0.871
Adducts/Neutral losses	464	464	263	263	neg_1026	neg_894	neutral loss	CH2O	CH2O	2.200	2.162	359.0974	329.0873	0.883
Adducts/Neutral losses	464	464	570	263	pos_2248	neg_894	pol / adduct	[M+Na]+	[M-H]-	2.161	2.162	353.0833	329.0873	0.776
MSDial	464	464	263	263	neg_1115	neg_894	Pearson correlation	[M-H]-	[M-H]-	2.155	2.162	382.0995	329.0873	0.904
MSDial	464	464	263	263	neg_1092	neg_894	Pearson correlation	[M-H]-	[M-H]-	2.161	2.162	375.0924	329.0873	0.948
Adducts/Neutral losses	464	464	263	263	neg_1092	neg_894	neutral loss	CH2O2	CH2O2	2.161	2.162	375.0924	329.0873	0.948
MSDial	464	464	570	570	pos_2195	pos_2248	pol / adduct	[M+NH4]+	[M+Na]+	2.161	2.161	348.1285	353.0833	1.000
MSDial	464	464	570	570	pos_2195	pos_2248	Pearson correlation	[M+NH4]+	[M+Na]+	2.161	2.161	348.1285	353.0833	0.918
MSDial	464	464	570	570	pos_2195	pos_2570	Pearson correlation	[M+NH4]+	[M+H]+	2.161	2.159	348.1285	384.1149	0.938
MSDial	464	464	570	570	pos_2570	pos_634	similar chromatogram in higher mz	[M+H]+	[M+H]+	2.159	2.163	384.1149	192.0650	1.000
MSDial	464	464	570	570	pos_2570	pos_634	Pearson correlation	[M+H]+	[M+H]+	2.159	2.163	384.1149	192.0650	0.901
MSDial	464	464	570	570	pos_2195	pos_634	Pearson correlation	[M+NH4]+	[M+H]+	2.161	2.163	348.1285	192.0650	0.931
Adducts/Neutral losses	465	465	572	250	pos_2756	neg_1204	pol / adduct	[M+H]+	[M-H]-	0.769	0.762	407.0414	405.0274	0.623
Adducts/Neutral losses	465	465	572	250	pos_643	neg_304	pol / adduct	[M+H]+	[M-H]-	0.764	0.755	193.0338	191.0192	0.745
Adducts/Neutral losses	465	465	572	250	pos_805	neg_304	pol / adduct	[M+Na]+	[M-H]-	0.770	0.755	215.0155	191.0192	0.827
MSDial	465	465	250	250	neg_1204	neg_304	found in higher mz's MSMS	[M+Na-2H]-	[M-H]-	0.762	0.755	405.0274	191.0192	1.000

MSDial	465	465	250	250	neg_304	neg_73	found in higher mz's MSMS	[M-H]$^-$	[M-H]$^-$	0.755	0.754	191.0192	111.0085	1.000
MSDial	465	465	250	250	neg_304	neg_73	Pearson correlation	[M-H]$^-$	[M-H]$^-$	0.755	0.754	191.0192	111.0085	0.982
MSDial	465	465	250	250	neg_1204	neg_73	found in higher mz's MSMS	[M+Na-2H]$^-$	[M-H]$^-$	0.762	0.754	405.0274	111.0085	1.000
MSDial	465	465	572	572	pos_2756	pos_643	Pearson correlation	[M+H]$^+$	[M+H]$^+$	0.769	0.764	407.0414	193.0338	0.902
MSDial	465	465	572	572	pos_1772	pos_643	Pearson correlation	[M+H]$^+$	[M+H]$^+$	0.763	0.764	310.1127	193.0338	0.947
MSDial	465	465	572	572	pos_643	pos_805	Pearson correlation	[M+H]$^+$	[M+H]$^+$	0.764	0.770	193.0338	215.0155	0.913
MSDial	467	467	576	576	pos_115	pos_1521	found in higher mz's MSMS	[M+H-H_2O]$^+$	[M+H]$^+$	5.265	5.265	111.1166	287.2214	1.000
Adducts/Neutral losses	467	467	576	576	pos_1703	pos_1521	neutral loss	H_3N	H_3N	5.265	5.265	304.2471	287.2214	0.990
MSDial	467	467	576	576	pos_115	pos_1521	Pearson correlation	[M+H-H_2O]$^+$	[M+H]$^+$	5.265	5.265	111.1166	287.2214	0.969
MSDial	467	467	576	576	pos_115	pos_1703	Pearson correlation	[M+H-H_2O]$^+$	[M+NH_4]$^+$	5.265	5.265	111.1166	304.2471	0.972
MSDial	467	467	576	576	pos_1521	pos_1703	Pearson correlation	[M+H]$^+$	[M+NH_4]$^+$	5.265	5.265	287.2214	304.2471	0.990
MSDial	467	467	576	576	pos_115	pos_1703	found in higher mz's MSMS	[M+H-H_2O]$^+$	[M+NH_4]$^+$	5.265	5.265	111.1166	304.2471	1.000
MSDial	467	467	576	576	pos_1521	pos_1762	Pearson correlation	[M+H]$^+$	[M+Na]$^+$	5.265	5.263	287.2214	309.2030	0.955
MSDial	467	467	576	576	pos_1703	pos_1762	Pearson correlation	[M+NH_4]$^+$	[M+Na]$^+$	5.265	5.263	304.2471	309.2030	0.964
MSDial	467	467	576	576	pos_1703	pos_1762	pol / adduct	[M+NH_4]$^+$	[M+Na]$^+$	5.265	5.263	304.2471	309.2030	1.000
MSDial	467	467	576	576	pos_1521	pos_1762	pol / adduct	[M+H]$^+$	[M+Na]$^+$	5.265	5.263	287.2214	309.2030	1.000
MSDial	467	467	576	576	pos_115	pos_1762	Pearson correlation	[M+H-H_2O]$^+$	[M+Na]$^+$	5.265	5.263	111.1166	309.2030	0.966
MSDial	467	467	576	576	pos_1703	pos_1871	Pearson correlation	[M+NH_4]$^+$	[M+H]$^+$	5.265	5.266	304.2471	318.2997	0.960
MSDial	467	467	576	576	pos_1521	pos_1871	Pearson correlation	[M+H]$^+$	[M+H]$^+$	5.265	5.266	287.2214	318.2997	0.958
MSDial	467	467	576	576	pos_1762	pos_1871	Pearson correlation	[M+Na]$^+$	[M+H]$^+$	5.263	5.266	309.2030	318.2997	0.949
MSDial	467	467	576	576	pos_115	pos_1871	Pearson correlation	[M+H-H_2O]$^+$	[M+H]$^+$	5.265	5.266	111.1166	318.2997	0.947
MSDial	467	467	576	576	pos_1991	pos_2418	Pearson correlation	[M+CH_3OH+H]$^+$	[M+H]$^+$	5.210	5.257	329.2948	369.1242	0.903
MSDial	467	467	576	576	pos_1521	pos_2418	Pearson correlation	[M+H]$^+$	[M+H]$^+$	5.265	5.257	287.2214	369.1242	0.933
MSDial	467	467	576	576	pos_1703	pos_2418	Pearson correlation	[M+NH_4]$^+$	[M+H]$^+$	5.265	5.257	304.2471	369.1242	0.924
MSDial	467	467	576	576	pos_1762	pos_2418	Pearson correlation	[M+Na]$^+$	[M+H]$^+$	5.263	5.257	309.2030	369.1242	0.914

MSDial	467	467	576	576	pos_1871	pos_2418	Pearson correlation	$[M+H]^+$	$[M+H]^+$	5.266	5.257	318.2997	369.1242	0.925
MSDial	467	467	576	576	pos_115	pos_689	similar chromatogram in higher mz	$[M+H-H_2O]^+$	$[M+H]^+$	5.265	5.264	111.1166	199.1692	1.000
MSDial	467	467	576	576	pos_2418	pos_689	Pearson correlation	$[M+H]^+$	$[M+H]^+$	5.257	5.264	369.1242	199.1692	0.923
MSDial	467	467	576	576	pos_1762	pos_689	Pearson correlation	$[M+Na]^+$	$[M+H]^+$	5.263	5.264	309.2030	199.1692	0.977
MSDial	467	467	576	576	pos_115	pos_689	found in higher mz's MSMS	$[M+H-H_2O]^+$	$[M+H]^+$	5.265	5.264	111.1166	199.1692	1.000
MSDial	467	467	576	576	pos_1703	pos_689	Pearson correlation	$[M+NH_4]^+$	$[M+H]^+$	5.265	5.264	304.2471	199.1692	0.984
MSDial	467	467	576	576	pos_1871	pos_689	Pearson correlation	$[M+H]^+$	$[M+H]^+$	5.266	5.264	318.2997	199.1692	0.958
MSDial	467	467	576	576	pos_1521	pos_689	Pearson correlation	$[M+H]^+$	$[M+H]^+$	5.265	5.264	287.2214	199.1692	0.972
MSDial	467	467	576	576	pos_1871	pos_689	similar chromatogram in higher mz	$[M+H]^+$	$[M+H]^+$	5.266	5.264	318.2997	199.1692	1.000
MSDial	467	467	576	576	pos_115	pos_689	Pearson correlation	$[M+H-H_2O]^+$	$[M+H]^+$	5.265	5.264	111.1166	199.1692	0.959
MSDial	472	472	483	483	pos_1072	pos_755	Pearson correlation	$[M+Na]^+$	$[M+NH_4]^+$	4.340	4.322	245.0782	208.1902	0.932
MSDial	472	472	483	483	pos_1455	pos_755	similar chromatogram in higher mz	$[M+Na]^+$	$[M+NH_4]^+$	4.315	4.322	281.0797	208.1902	1.000
Adducts/Neutral losses	474	474	585	274	pos_85	neg_61	pol / adduct	$[M+H]^+$	[M-H]-	1.134	1.135	101.0232	99.0086	0.965
Adducts/Neutral losses	474	474	274	274	neg_86	neg_61	neutral loss	H_2O	H_2O	1.137	1.135	117.0191	99.0086	0.994
MSDial	474	474	274	274	neg_61	neg_86	found in higher mz's MSMS	$[M-H_2O-H]^-$	$[M-H]^-$	1.135	1.137	99.0086	117.0191	1.000
Adducts/Neutral losses	474	474	585	274	pos_265	neg_86	pol / adduct	$[M+Na]^+$	$[M-H]^-$	1.128	1.137	141.0155	117.0191	0.962
MSDial	474	474	274	274	neg_61	neg_86	Pearson correlation	$[M-H_2O-H]^-$	$[M-H]^-$	1.135	1.137	99.0086	117.0191	0.994
MSDial	474	474	585	585	pos_265	pos_85	Pearson correlation	$[M+Na]^+$	$[M+H-H_2O]^+$	1.128	1.134	141.0155	101.0232	0.979
Adducts/Neutral losses	476	476	377	170	pos_4330	neg_1920	pol / adduct	$[M+NH_4]^+$	$[M-H]^-$	4.913	4.913	828.4357	809.3953	0.812
MSDial	476	476	170	170	neg_1920	neg_599	similar chromatogram in higher mz	$[M-H]^-$	$[M-H]^-$	4.913	4.906	809.3953	266.1026	1.000
MSDial	476	476	377	377	pos_1014	pos_1076	Pearson correlation	$[M+H]^+$	$[M+H]^+$	5.055	5.008	239.1429	245.1738	0.931
MSDial	476	455	377	562	pos_1014	pos_1213	Pearson correlation	$[M+H]^+$	$[M+K]^+$	5.055	5.101	239.1429	259.1894	0.901
MSDial	476	476	377	377	pos_1562	pos_1964	Pearson correlation	$[M+H]^+$	$[M+H]^+$	4.925	4.960	290.2684	327.0774	0.948

MSDial	476	476	377	377	pos_1076	pos_1964	Pearson correlation	$[M+H]^+$	$[M+H]^+$	5.008	4.960	245.1738	327.0774	0.910
Adducts/Neutral losses	476	476	377	377	pos_348	pos_233	neutral loss	H_2O	H_2O	4.883	4.882	153.1271	135.1166	0.972
MSDial	476	476	377	377	pos_1562	pos_233	Pearson correlation	$[M+H]^+$	$[M+H-H_2O]^+$	4.925	4.882	290.2684	135.1166	0.945
MSDial	476	476	377	377	pos_233	pos_348	pol / adduct	$[M+H-H_2O]^+$	$[M+H]^+$	4.882	4.883	135.1166	153.1271	1.000
MSDial	476	476	377	377	pos_1562	pos_348	Pearson correlation	$[M+H]^+$	$[M+H]^+$	4.925	4.883	290.2684	153.1271	0.959
MSDial	476	476	377	377	pos_1098	pos_348	Pearson correlation	$[M+H]^+$	$[M+H]^+$	4.919	4.883	247.1665	153.1271	0.901
MSDial	476	476	377	377	pos_233	pos_348	Pearson correlation	$[M+H-H_2O]^+$	$[M+H]^+$	4.882	4.883	135.1166	153.1271	0.972
Adducts/Neutral losses	476	476	377	377	pos_1098	pos_348	neutral loss	C_6H_6O	C_6H_6O	4.919	4.883	247.1665	153.1271	0.901
MSDial	476	476	377	377	pos_233	pos_4330	found in higher mz's MSMS	$[M+H-H_2O]^+$	$[M+H]^+$	4.882	4.913	135.1166	828.4357	1.000
Adducts/Neutral losses	476	476	377	377	pos_870	pos_630	neutral loss	CH_3OH	CH_3OH	4.885	4.883	223.2047	191.1788	0.952
MSDial	476	476	377	377	pos_233	pos_630	Pearson correlation	$[M+H-H_2O]^+$	$[M+H]^+$	4.882	4.883	135.1166	191.1788	0.934
MSDial	476	476	377	377	pos_233	pos_630	found in higher mz's MSMS	$[M+H-H_2O]^+$	$[M+H]^+$	4.882	4.883	135.1166	191.1788	1.000
MSDial	476	476	377	377	pos_1562	pos_630	Pearson correlation	$[M+H]^+$	$[M+H]^+$	4.925	4.883	290.2684	191.1788	0.955
Adducts/Neutral losses	476	476	377	377	pos_870	pos_630	neutral loss	CH_4O	CH_4O	4.885	4.883	223.2047	191.1788	0.952
MSDial	476	476	377	377	pos_348	pos_630	Pearson correlation	$[M+H]^+$	$[M+H]^+$	4.883	4.883	153.1271	191.1788	0.950
MSDial	476	476	377	377	pos_233	pos_870	Pearson correlation	$[M+H-H_2O]^+$	$[M+CH_3OH+H]^+$	4.882	4.885	135.1166	223.2047	0.951
MSDial	476	476	377	377	pos_1562	pos_870	Pearson correlation	$[M+H]^+$	$[M+CH_3OH+H]^+$	4.925	4.885	290.2684	223.2047	0.956
MSDial	476	476	377	377	pos_630	pos_870	pol / adduct	$[M+H]^+$	$[M+CH_3OH+H]^+$	4.883	4.885	191.1788	223.2047	1.000
MSDial	476	476	377	377	pos_233	pos_870	found in higher mz's MSMS	$[M+H-H_2O]^+$	$[M+CH_3OH+H]^+$	4.882	4.885	135.1166	223.2047	1.000
MSDial	476	476	377	377	pos_348	pos_870	Pearson correlation	$[M+H]^+$	$[M+CH_3OH+H]^+$	4.883	4.885	153.1271	223.2047	0.952
MSDial	476	476	377	377	pos_630	pos_870	Pearson correlation	$[M+H]^+$	$[M+CH_3OH+H]^+$	4.883	4.885	191.1788	223.2047	0.952
Adducts/Neutral losses	479	479	54	54	neg_1439	neg_1320	neutral loss	CO_2	CO_2	2.048	2.049	485.1501	441.1603	0.862

Adducts/Neutral losses	479	479	591	54	pos_2815	neg_1320	pol / adduct	$[M+NH_4]^+$	[M+FA-H]$^-$	2.051	2.049	414.1953	441.1603	0.900
MSDial	479	479	54	54	neg_1320	neg_1439	similar chromatogram in higher mz	[M-H]$^-$	[M-H]$^-$	2.049	2.048	441.1603	485.1501	1.000
Adducts/Neutral losses	479	479	591	241	pos_3722	neg_1642	pol / adduct	[M+H]$^+$	[M-H]$^-$	2.103	2.101	566.1541	564.1379	0.886
Adducts/Neutral losses	479	479	584	241	pos_846	neg_411	pol / adduct	[M+H]$^+$	[M-H]$^-$	2.135	2.136	220.1171	218.1029	0.789
MSDial	479	479	241	241	neg_1245	neg_411	similar chromatogram in higher mz	[M-H]$^-$	[M-H]$^-$	2.133	2.136	418.1343	218.1029	1.000
MSDial	479	479	241	241	neg_1642	neg_411	Pearson correlation	[M-H]$^-$	[M-H]$^-$	2.101	2.136	564.1379	218.1029	0.949
Adducts/Neutral losses	479	479	591	207	pos_906	neg_440	pol / adduct	[M+H]$^+$	[M-H]$^-$	2.045	2.042	227.1019	225.0876	0.820
MSDial	479	479	241	241	neg_411	neg_688	found in higher mz's MSMS	[M-H]$^-$	[M-H]$^-$	2.136	2.137	218.1029	286.0902	1.000
Adducts/Neutral losses	479	479	241	241	neg_1245	neg_688	neutral loss	D-ribose-H_2O	D-ribose-H_2O	2.133	2.137	418.1343	286.0902	0.669
MSDial	479	479	241	241	neg_411	neg_688	Pearson correlation	[M-H]$^-$	[M-H]$^-$	2.136	2.137	218.1029	286.0902	0.955
MSDial	479	479	241	241	neg_1642	neg_688	Pearson correlation	[M-H]$^-$	[M-H]$^-$	2.101	2.137	564.1379	286.0902	0.908
MSDial	479	479	241	241	neg_688	neg_788	pol / adduct	[M-H]$^-$	[M+Na-2H]$^-$	2.137	2.139	286.0902	308.0980	1.000
MSDial	479	479	591	591	pos_3722	pos_858	similar chromatogram in higher mz	[M+H]$^+$	[M+H]$^+$	2.103	2.088	566.1541	222.0792	1.000
MSDial	479	479	591	591	pos_1055	pos_858	similar chromatogram in higher mz	[M+H]$^+$	[M+H]$^+$	2.085	2.088	243.1808	222.0792	1.000
MSDial	479	479	591	591	pos_2815	pos_906	similar chromatogram in higher mz	[M+H]$^+$	$[M+NH_4]^+$	2.051	2.045	414.1953	227.1019	1.000
MSDial	479	479	591	591	pos_858	pos_906	Pearson correlation	[M+H]$^+$	$[M+NH_4]^+$	2.088	2.045	222.0792	227.1019	0.910
Adducts/Neutral losses	480	480	592	116	pos_3977	neg_1769	pol / adduct	[M+H]$^+$	[M-H]$^-$	4.135	4.129	643.2747	641.2607	0.600
Adducts/Neutral losses	480	480	592	211	pos_788	neg_450	pol / adduct	$[M+H-H_2O]^+$	[M-H]$^-$	4.178	4.178	212.1644	228.1599	0.935
Adducts/Neutral losses	480	480	592	211	pos_1139	neg_450	pol / adduct	[M+Na]$^+$	[M-H]$^-$	4.180	4.178	252.1564	228.1599	0.935
Adducts/Neutral losses	480	480	592	592	pos_4204	pos_3977	neutral loss	Malonyl-H_2O)	malonyl-H_2O	4.156	4.135	729.2748	643.2747	0.757
MSDial	480	480	592	592	pos_1139	pos_788	pol / adduct	[M+Na]$^+$	$[M+H-H_2O]^+$	4.180	4.178	252.1564	212.1644	1.000

MSDial	480	480	592	592	pos_1139	pos_788	Pearson correlation	$[M+Na]^+$	$[M+H-H_2O]^+$	4.180	4.178	252.1564	212.1644	0.945
MSDial	480	480	592	592	pos_2660	pos_788	similar chromatogram in higher mz	$[M+H]^+$	$[M+H-H_2O]^+$	4.172	4.178	394.2427	212.1644	1.000
MSDial	480	480	592	592	pos_4204	pos_788	similar chromatogram in higher mz	$[M+H]^+$	$[M+H-H_2O]^+$	4.156	4.178	729.2748	212.1644	1.000
MSDial	480	480	592	592	pos_3977	pos_908	found in higher mz's MSMS	$[M+H]^+$	$[M+Na]^+$	4.135	4.148	643.2747	227.1245	1.000
MSDial	480	480	592	592	pos_4204	pos_908	found in higher mz's MSMS	$[M+H]^+$	$[M+Na]^+$	4.156	4.148	729.2748	227.1245	1.000

Table S3. Pathways (KEGG) connected to the metabolite profiles determined by HILIC- and RPLC-HRMS in the differently-processed spinach samples. .

Pathway name[a]	LC column	Total hits[b]	Significant hits[c]	$-\log_{10}$ (p)[d]	LC column	Total hits[b]	Significant hits[c]	$-\log_{10}$ (p)[d]
C –untreated samples								
Valine, leucine and isoleucine degradation	-	-	-	-	RPLC	12	4	3.470
Alanine, aspartate and glutamate metabolism	-	-	-	-	RPLC	13	4	3.456
TAP-washed samples								
Phenylalanine, tyrosine and tryptophan biosyn.	-	-	-	-	RPLC	21	5	3.442
Aminoacyl-tRNA biosynthesis	HILIC	58	7	3.157	RPLC	24	5	3.448
Phenylalanine metabolism	-	-	-	-	RPLC	11	2	3.161
PAW-treated samples								
Butanoate metabolism	-	-	-	-	RPLC	8	6	3.289
Cysteine and methionine metabolism	-	-	-	-	RPLC	21	9	3.285
C5-branched dibasic acid metabolism	HILIC	22	6	2.673	RPLC	5	4	3.284
Propanoate metabolism	-	-	-	-	RPLC	9	5	3.277
Aminoacyl-tRNA biosynthesis	-	-	-	-	RPLC	24	9	3.538
Alanine, aspartate and glutamate metabolism	-	-	-	-	RPLC	13	6	3.272
Cyanoamino acid metabolism	-	-	-	-	RPLC	23	8	3.250
Ascorbate and aldarate metabolism	HILIC	27	9	2.749	-	-	-	-
Starch and sucrose metabolism	HILIC	14	6	2.747	-	-	-	-
Galactose metabolism	HILIC	25	8	2.741	-	-	-	-
Glycerolipid metabolism	HILIC	14	3	2.315	-	-	-	-

[a]Pathways: Metabolic pathways (KEGG, http://www.genome.jp/kegg/) as identified by the *mummichog* algorithm [9] in Metaboanalyst 5.0 [10]. [b]Total hits: the total number of empirical metabolite hits in the sample allocatable to a specific pathway. [c]Significant hits: The number of hits considered as significant based on the p-value cutoff used on the input data for the *mummichog* algorithm. [d]$-\log_{10}$ (p): Gamma-adjusted p-values describing the probabilities of correctly identifying the respective metabolite pathways. Displayed as $-\log_{10}$ in **Figure 4**.
